# Study on the Fluorescent Activity of *N*^2^-Indolyl-1,2,3-triazole

**DOI:** 10.3390/molecules22091380

**Published:** 2017-09-05

**Authors:** You-Can Zhang, Rui Jin, Luo-Yuan Li, Zili Chen, Li-Min Fu

**Affiliations:** Department of Chemistry, Renmin University of China, Beijing 100872, China; 2015101964@ruc.edu.cn (Y.-C.Z.); jinrui87@ruc.edu.cn (R.J.); luoyuanli1209@126.com (L.-Y.L.)

**Keywords:** *N*^2^-indolyl-1,2,3-triazoles (NITs), fluorescent activity, blue-emissive fluorophore, *N*^2^-aryl-1,2,3-triazoles (NATs)

## Abstract

A new type of blue emitter, *N*^2^-Indolyl-1,2,3-triazoles (NITs), with the λ_max_ ranging from 420–480 nm and the Stokes shift from 89–143 nm, were synthesized through the coupling reaction of indoles with triazole derivatives. The influence of different substitution patterns on the optical properties (efficiency, excitation, and emission wavelengths) of the NITs was investigated. In addition, one palladium complex were synthesized by using NITs as the ligands, which, however, exhibited no fluorescent activity, but did show the enhanced co-planarity. Lastly, two bio-active molecule derivatives were explored for the potential use of these novel dyes in related chemical and biological applications.

## 1. Introduction

Photoactive molecules have been the object of many studies in chemistry, biology, and material research [1,2,3,4,5]. A great number of fluorescent compounds emitting different colors (such as red, green, etc.) has been developed over the past years for their utilization in the biological target imaging [6,7,8,9,10,11], effective photosensors [12,13,14,15,16,17], and novel photoactive materials [18,19,20,21,22,23]. However, the corresponding blue-emissive fluorophores, which exhibit blue fluorescence in high quantum efficiency coupled with high stability, are more difficult to obtain [24,25,26,27,28], due to the large energy gaps that exist between the blue emitter’s highest occupied molecular orbitals (HOMOs) and its lowest unoccupied molecular orbitals (LUMOs) [29,30].

This optical activity and photostability dilemma is a great challenge for the development of efficient, stable blue-light-emitting small molecular fluorophores. For example, biphenyl-type molecules and similar structures usually have lower photoactivity, because of the poor conjugation and the ortho-substituted group’s steric repulsions [31,32,33]. On the other hand, extended aromatic systems, in which at least one double bond exists between the aromatic groups, have high quantum efficient blue emission. However, the photolabile double bonds limit their potential applications [34,35,36,37,38].

**Scheme 1:**
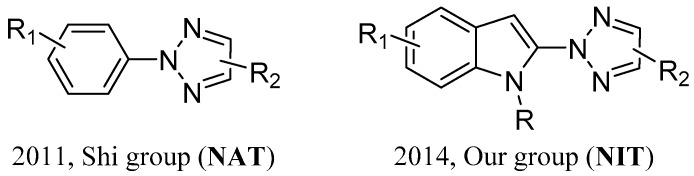
The structure of *N*-2-Aryl-1,2,3-triazoles (NATs) & *N*-2-Indolyl-1,2,3-triazoles (NITs).

In 2011, Shi and his coworkers reported *N^2^*-Aryl-1,2,3-triazoles (NATs, Scheme 1) as a new type of blue-light emitting fluorophore, featured by its tunable optical emission (λ_max_ range from 350–400 nm and Stokes shift from 38–93 nm), with a moderate to good quantum efficiency and a relatively higher stability [39,40]. It was proposed that the blue fluorescence of NAT resulted from a planar intramolecular charge transfer (PICT) mechanism.

**Scheme 2:**
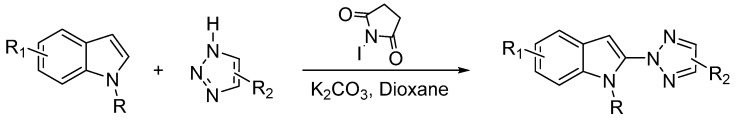
Method for the preparation of various substituted *N^2^*-Indolyl-1,2,3-triazole (NITs).

Recently, we developed a similar molecular structure, *N^2^*-Indolyl-1,2,3-triazole (NITs) through the *N^2^*-selective coupling of 1,2,3-triazoles with indoles via iodo-mediation, which are also good blue emitters, with the λ_max_ ranging from 420–480 nm and the Stokes shift from 89–143 nm [41,42]. Starting from this structure, we synthesized a new single-fluorophore-based fluorescent probe for the dual-channel detection of Ag^+^ and Hg^2+^ ions [43]. As an extension of the previous research, a series of NITs with different substitution patterns were prepared using the method shown in Scheme 2, and the optical properties of these new blue emitters were investigated in this paper. In addition, one palladium complexes were synthesized by using NITs as ligands, which, however, exhibited no fluorescent activity, but did present enhanced co-planarity.

## 2. Results and Discussions

The structures of the NITs allowed modification on three different positions: the indole nitrogen atom (R group), the triazole moiety (R_1_ group), and the indole ring (R_2_ group) (Scheme 2). To evaluate these new fluorophores, we synthesized compounds **3a**–**y** and explored the influence of different substitution patterns on the optical properties (efficiency, excitation, and emission wavelengths) of the NITs. 

Influence of the indole *N*-position: At first, the effect of *N-*substituted R group was investigated. It was proposed that the fluorescence activity of NATs the was greatly affected by the co-planarity between the phenyl ring and the triazole group (Scheme 1) [39,40]. We considered that R group in NITs, which was located nearby the rotating *C-N* bond between the indole ring and the triazole group, would affect the co-planarity of NITs, and thus influence their optical properties.

**Figure 1:**
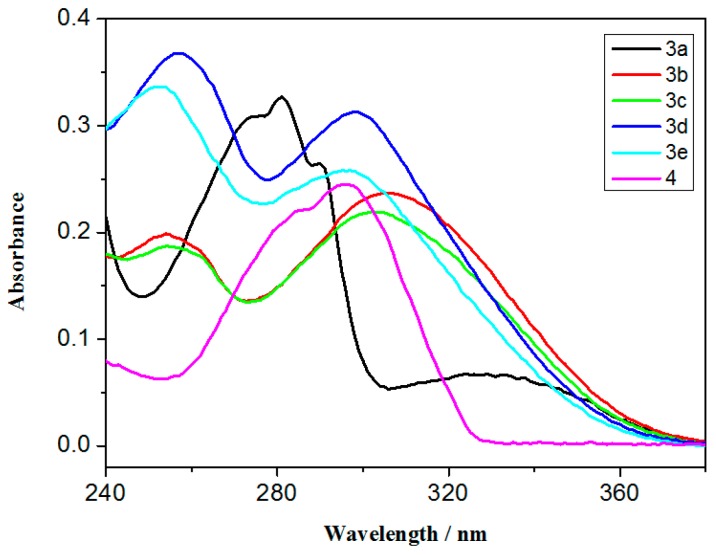
The absorption spectra of compounds **3**(**a**–**e**), **4**.

**Figure 2:**
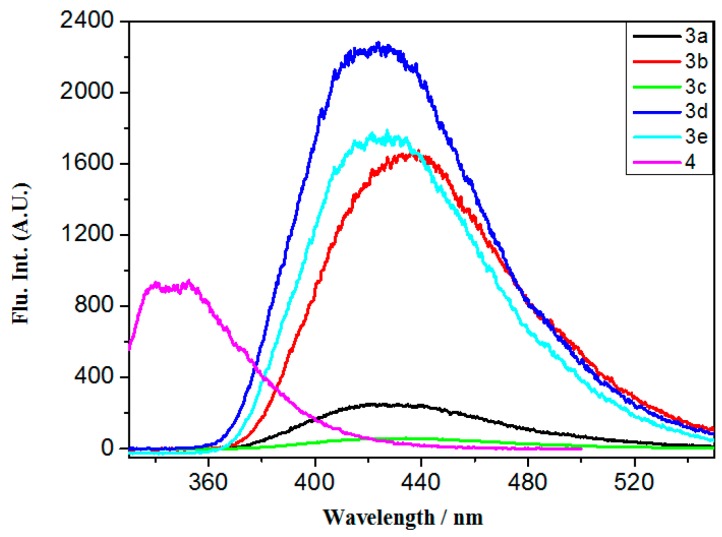
The emission spectra of compounds **3**(**a**–**e**), **4**.

The absorption and emission behavior of compounds **3a**–**e** are summarized in Table 1, Figure 1 and Figure 2. A NAT compound, 2,4-diphenyl-2*H*-1,2,3-triazole (compound **4**), was tested as the comparison [39,40], because of its structural similarity with NIT molecules and its synthetic convenience. As shown in Table 1, compound **4** absorbs UV light at 292 nm and emits blue fluorescence at around 345 nm. It was found that the UV absorbance of NITs **3a**–**e** ranged from 298 nm to 325 nm. The peaks of their fluorescence emission had an obvious red-shift compared with **4**, which might result from indole group’s stronger electro-donor ability compared to the *N*-phenyl group of NATs, as well as the larger conjugated system. In addition, all NIT compounds’ Stokes shifts are larger than that of NAT molecule **4**.

As shown in Table 1, the gradual blue shift of the UV absorbance of **3a**–**e** correlated well with the bulkiness of the corresponding NIT molecule’s indole *N-*substitution. Compared with indole *N-*alkyl or phenyl substituted **3b**–**e** (Table 1, entries 3–6), *N-*unsubstituted compound **3a** gave a relative weak fluorescence emission (centered at 429 nm), possibly because of the intense solvation effect of the bare *N-*H bond in dichloromethane (DCM) [44,45]. The highly steric hindered compounds **3d**–**e** with a bulky *N-*phenyl group gave similar strong emissions as the much less hindered compounds **3b** and **3c**, which suggested the small influence of the bulkiness on the indole nitrogen position for the light emission. This effect was also confirmed by the slight red-shift of the emission of **3b**–**c** compared with the fluorescence of **3a**. Moreover, the gradual increase in the Stokes shift of **3b**–**e** (from 117–127 nm) indicated a much larger difference between the electronic structure of **3b**–**e** in the ground state and those in the excited state [46]. Notably, the quantum yields of **3b**–**e** were very high; in particular, the quantum yield of **3e** reached up to 0.99.

**Figure 3:**
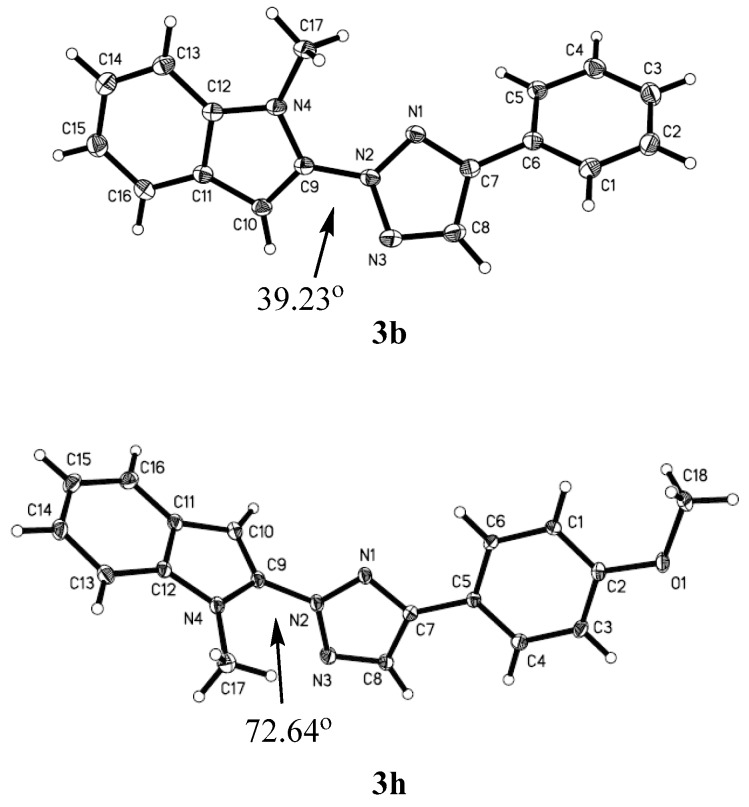
X-ray crystal structures of **3b** and **3h**.

Compared with NAT molecule **4**, NIT compounds **3a**–**e** presented large, quite interesting Stokes shifts; their electronic structure in the excited state should be conspicuously varied from those in the ground state. In order to determine the NIT molecule’s co-planarity in the ground state, the X-ray crystal structure of compounds **3b** and **3h** were studied. As shown in Figure 3, the crystal structure of both **3b** and **3h** exhibited a very large dihedral angle between the indole ring and the triazole ring (see Supplementary Materials). **3h** in particular exhibited a dihedral angle of up to 72.64°. These compounds’ real dihedral angles might be different when dissolved in a solution. However, considering that an intramolecular charge transfer emitter always tends to take a more planar-like configuration at its excited state [47,48], the wide dihedral angles shown in Figure 3 partly verified the large Stokes shift exhibited by NIT molecules in Table 1.

**Figure 4:**
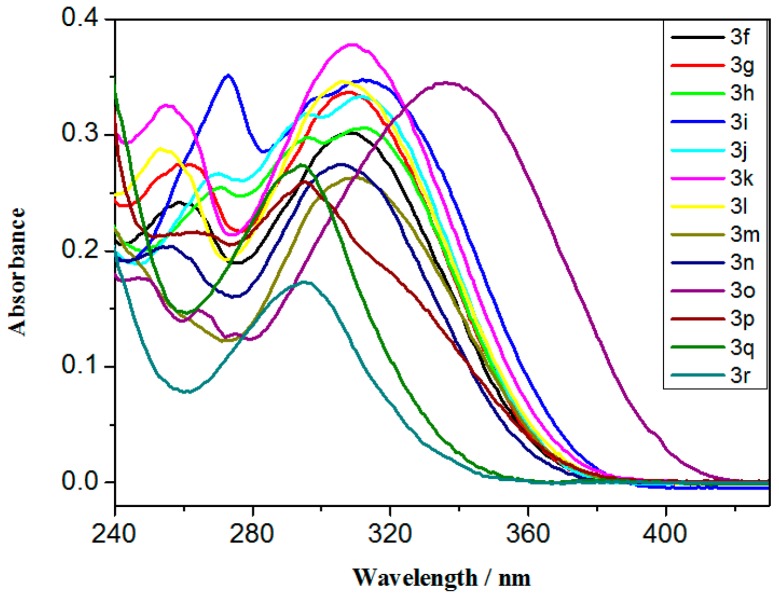
The absorption spectra of compounds **3**(**f**–**r**).

**Figure 5:**
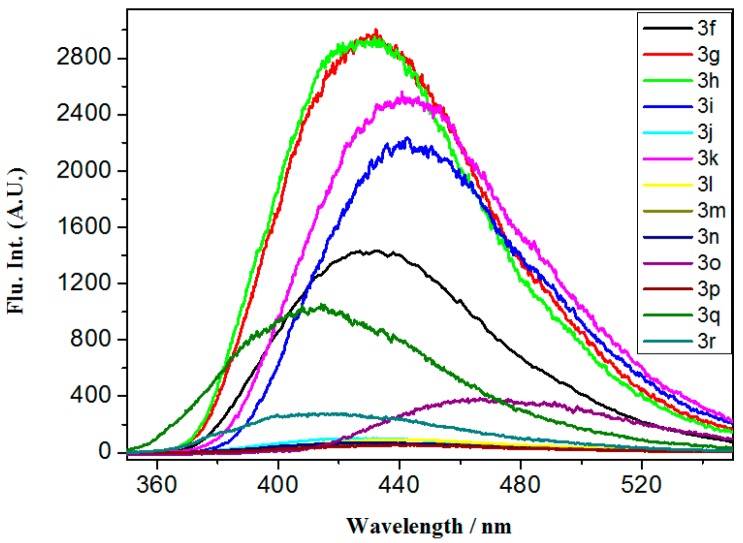
The emission spectra of compounds **3**(**f**–**r**).

The effect of triazole substituent R_1_ group (Scheme 2) was then explored. As shown in Table 2, various triazole substitution patterns, such as phenyl (**3f**–**m** and **3o**–**p**), thienyl (**3n**), and alkyl (**3q**) groups were studied. It was found their UV absorbance ranged from 294 nm to 340 nm (Figure 4), while their fluorescence emission varied from 414 nm to 467 nm (Figure 5). The substituents’ electronegativity would affect the optical properties of the NITs. For example, a slight (10–20 nm) red shift of the fluorescence emission peak and the large Stokes shift of **3l** was due to its strong donor-acceptor ability [49,50,51]. The emission peaks of **3i** and **3o** showed obvious red shifts, because of the presence of an additional ethynyl group (**3i**) and a planar benzotriazole group (**3o**). Nevertheless, compared with **3f**–**p**, the UV absorbance and fluorescence emission of alkyl-substituted **3q** and unsubstituted **3r** have obvious blue shifts, due to the absence of a conjugated phenyl or thienyl group [52,53,54]. The slight red shift of the emission and large Stokes shift of **3m** might have resulted from an intramolecular hydrogen bond between the triazole group and the neighboring hydroxyl group. These NIT molecules’ quantum yields ranged between 0.14–0.84. **3o** and **3p** have relative small quantum yields, while **3k** and **3r** have much larger quantum yields, though their detailed affecting factors are still not clear.

**Figure 6:**
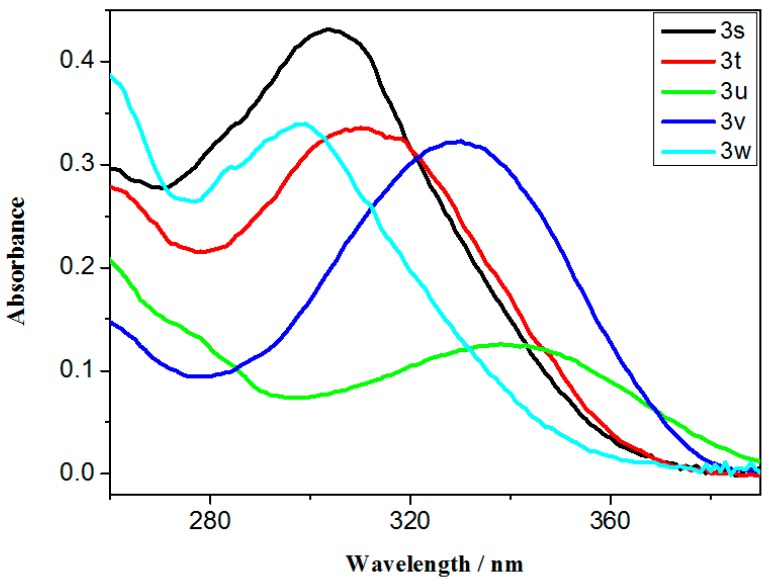
The absorption spectra of compounds **3**(**s**–**w**).

**Figure 7:**
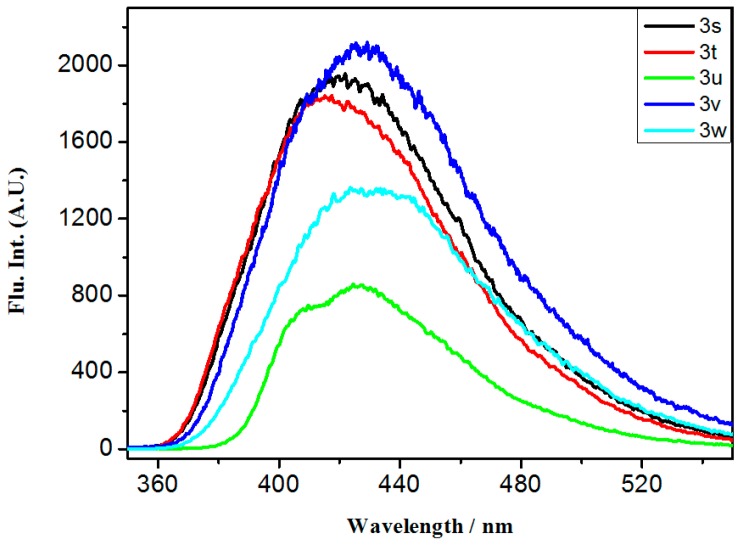
The emission spectra of compounds **3**(**f**–**r**).

We then explored the effect of the indole ring substituents. As compared with the fluoro group in **3s**, the TsO-group and AcNH-group’s low electronegativity as well as their conjugated lone pair improved the donor-acceptor ability of **3t** and especially **3u**, thus leading to the red shift of their UV absorbance and fluorescence emission (Figure 6 and Figure 7). The small Stokes shift of **3u** might be due to its better co-planarity in the ground state. As shown in Table 3, **3v** has a little red shift in its UV absorbance and fluorescence emission compared to **3a**, while its Stokes shift is lower than that of **3a**. An additional *N-*benzyl group would reduce the co-planarity of **3w** in the ground state, and then improve its Stokes shift.

**Scheme 3:**
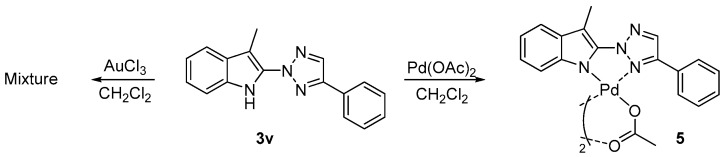
The reaction of **3w** with AuCl_3_ and Pd(OAc)_2_.

**Figure 8:**
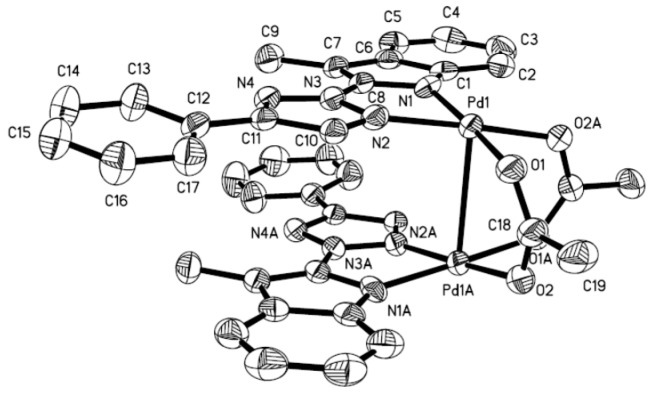
X-ray crystal structure of **5**.

We wondered if the metal complexation of the triazole group and the indole nitrogen atom would improve NIT molecules’ co-planarity and affect their optical properties. As shown in Scheme 3, the reactions of NIT molecule **3v** with metal salts AuCl_3_ and Pd(OAc)_2_ were tested, in which no desired Au(III) metal complex was obtained. However, the reaction of **3v** with 1 equivalent of Pd(OAc)_2_ in CH_2_Cl_2_ at room temperature (r.t.) gave a white solid **5** in 45% yield (Scheme 3). As confirmed by its X-ray crystal structure shown in Figure 8, the palladium complex was determined to be a metal complex dimer bridged by two acetates, rather like an open book. In complex **5**, it was found that the indole ring, the triazole ring, and the palladium atom almost located in a co-facial arrangement. The geometry around Pd(II) is square planar, composed of two nitrogen atoms from the indole ring and triazole ring, and two oxygen atoms from two bridging acetates. The two NIT ligands are close to parallel in an offset face-to-face stacking mode by a quite small dihedral angle of 0.54°, conforming to an approximate π–π interaction. The resulting Pd1···Pd2 separation of 2.849(2) Å is significantly shorter than the sum of the van der Waals radii of palladium (the typical value of which is 1.6 Å), which reveals a Pd···Pd interaction [55]. Previous studies on double-carboxylato-bridged dinuclear organometallic Pd(II) complexes have shown the possibility of *cis*-*trans* isomerism depending on the nature of the ligands [56,57,58]. It has been found that only the *trans* isomer of complex **5** crystallizes in the asymmetric unit.

However, complex **5** show no fluorescence under UV irradiation, possibly due to the fluorescence quenching by Pd^2+^ *d-d* transition.

**Figure 9:**
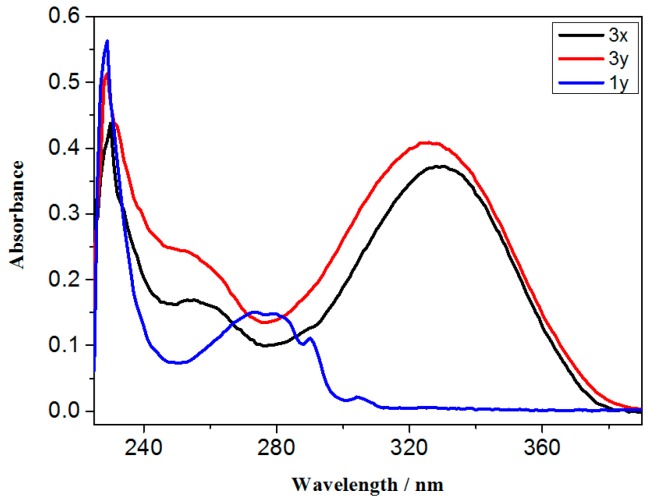
The absorption spectra of compounds **3x**, **3y**, **1y**.

**Figure 10:**
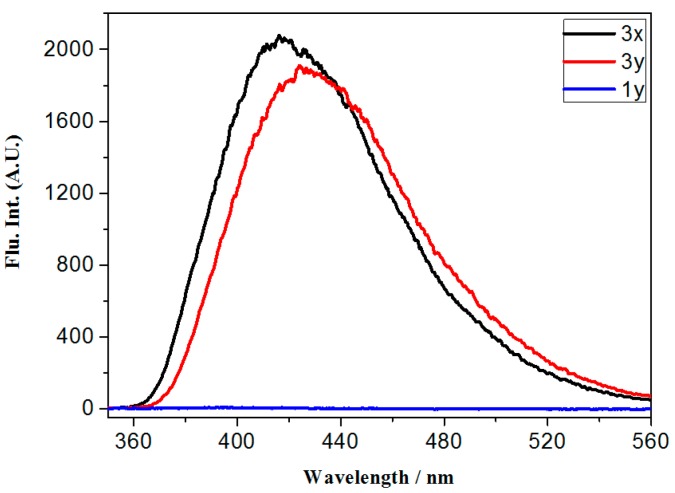
The emission spectra of compounds **3x**, **3y**, **1y**.

Finally, the optical properties of two bio-active molecule derivatives **3x** and **3y** were also explored, in which compound **3x** was obtained from the reaction of naturally occurring plant auxin indole-3-acetic acid methyl ester with *N*-1,2,3-phenyl triazole, and **3y** was derived from amino acid tryptophan. As shown in Table 4, compounds **3x** and **3y** absorb strongly at 326–328 nm (Figure 9) and emit vivid blue fluorescence (centered at 418 nm and 430 nm, Figure 10). In sharp contrast, the intrinsic tryptophan units have negligible absorption in the region with a wavelength longer than 320 nm and consequently no fluorescence under 330-nm excitation, which might enable a reliable detection of target biomolecules tagged with the abovementioned fluorescent tryptophan-triazole conjugate **3y** without the interference from tryptophan components. Considering the unique UV/blue emission of compound **3y** and its analogs, it is a potential new kind of UV fluorescence probe for various chemical and biological studies.

## 3. Experimental Section

General procedure for coupling reaction (condition 1) for the synthesis of **3a**–**3u**: To a suspension of *N*-iodo succinimide (0.3 mmol) and K_2_CO_3_ (0.5 mmol) in dry dioxane (1 mL), was added dropwise a solution of **1a** (0.2 mmol) and **2a** (0.1 mmol) in dioxane (1 mL) in 5 min. 30 min later, the reaction mixture was diluted with 20 mL EtOAc, and was then washed with saturated aqueous Na_2_S_2_O_3_ (5 mL), brine (10 mL) and water (10 mL). The organic phase was dried over anhydrous Na_2_SO_4_, filtered and concentrated in vacuo, purification of the crude product through flash chromatography (petroleum/EtOAc = 50/1) afforded **3a** as a white solid in 60% yield.

2-(4-Phenyl-2*H*-1,2,3-triazol-2-yl)-1*H*-indole **3a** [41]. Obtained as a white solid in 60% yield; m.p. 128–130 °C; ^1^H-NMR (400 MHz, CDCl_3_) δ 9.07 (br, 1H), 8.07 (s, 1H), 7.89 (d, *J* = 7.1 Hz, 2H), 7.66 (d, *J* = 7.8 Hz, 1H), 7.49 (t, *J* = 7.4 Hz, 2H), 7.42 (dd, *J* = 7.7, 2.8 Hz, 2H), 7.28–7.22 (m, 1H), 7.21–7.15 (m, 1H), 6.90 (d, *J* = 1.4 Hz, 1H); ^13^C-NMR (101 MHz, CDCl_3_) δ 149.05, 134.80, 133.54, 132.82, 129.44, 129.16, 129.06, 127.89, 126.22, 122.86, 121.04, 120.98, 111.20, 90.31.

1-Methyl-2-(4-phenyl-2*H*-1,2,3-triazol-2-yl)-1*H*-indole **3b** [41]. Obtained as a white solid in 64% yield; m.p. 90–91 °C; ^1^H-NMR (400 MHz, CDCl_3_) δ 8.11 (s, 1H), 7.88 (d, *J* = 7.1 Hz, 2H), 7.66 (d, *J* = 7.9 Hz, 1H), 7.46 (t, *J* = 7.4 Hz, 2H), 7.38 (dd, *J* = 12.9, 7.8 Hz, 2H), 7.30 (t, *J* = 7.1 Hz, 1H), 7.18 (t, *J* = 6.9 Hz, 1H), 6.84 (s, 1H), 3.83 (s, 3H); ^13^C-NMR (101 MHz, CDCl_3_) δ 149.08, 135.92, 135.44, 133.02, 129.61, 129.17, 129.10, 126.27, 122.96, 121.41, 120.71, 120.30, 109.83, 96.27, 30.56.

1-Benzyl-2-(4-phenyl-2*H*-1,2,3-triazol-2-yl)-1*H*-indole **3c** [41]. Obtained as a white solid in 72% yield; m.p. 91–92 °C; ^1^H-NMR (400 MHz, CDCl_3_) δ 8.01 (s, 1H), 7.77 (d, *J* = 7.2 Hz, 2H), 7.67 (d, *J* = 7.6 Hz, 1H), 7.39 (t, *J* = 7.3 Hz, 2H), 7.34 (d, *J* = 7.1 Hz, 1H), 7.28 (d, *J* = 8.1 Hz, 1H), 7.22 (d, *J* = 6.9 Hz, 1H), 7.20–7.11 (m, 4H), 7.04 (d, *J* = 7.0 Hz, 2H), 6.92 (s, 1H), 5.57 (s, 2H); ^13^C-NMR (101 MHz, CDCl_3_) δ 149.31, 137.45, 135.77, 135.39, 133.09, 129.60, 129.18, 129.10, 128.70, 127.44, 126.60, 126.44, 126.29, 123.30, 121.57, 121.06, 110.62, 96.96, 47.71.

1-Phenyl-2-(4-phenyl-2*H*-1,2,3-triazol-2-yl)-1*H*-indole **3d**. Obtained as a white solid in 57% yield; m.p. 91–93 °C; ^1^H-NMR (400 MHz, CDCl_3_) δ 8.02 (s, 1H), 7.92–7.78 (m, 3H), 7.43 (t, *J* = 20.5 Hz, 11H), 7.15 (s, 1H); ^13^C-NMR (101 MHz, CDCl_3_) δ 149.06, 136.91, 136.60, 135.28, 133.03, 129.70, 129.39, 129.08, 128.07, 127.56, 126.27, 123.87, 121.67, 121.63, 111.11, 98.86; IR (neat) 3061, 3028, 1607, 1534, 1461, 1356, 1055, 946, 768, 723, 692, 483 cm^−1^; HRMS(ESI) *m*/*z* calcd. for C_22_H_16_N_4_, [M + H]^+^ 337.1448, found 337.1444.

1-(4-Methoxyphenyl)-2-(4-phenyl-2*H*-1,2,3-triazol-2-yl)-1*H*-indole **3e**. Obtained as a white solid in 51% yield; m.p. 94–96 °C; ^1^H-NMR (400 MHz, CDCl_3_) δ 7.94 (s, 1H), 7.76–7.71 (m, 3H), 7.45–7.39 (m, 2H), 7.37 (d, *J* = 7.2 Hz, 1H), 7.27–7.22 (m, 5H), 6.97 (s, 1H), 6.91 (d, *J* = 8.9 Hz, 2H), 3.81 (s, 3H); ^13^C-NMR (101 MHz, CDCl_3_) δ 159.12, 148.87, 136.81, 135.28, 132.84, 129.51, 129.22, 128.95, 128.79, 126.15, 125.94, 123.56, 121.45, 121.25, 114.38, 110.97, 98.30, 55.56; IR (neat): 3118, 3035, 2924, 1589, 1496, 1454, 1328, 1091, 975, 858, 767, 688, 505cm^−1^; HRMS (ESI) calcd. for C_23_H_18_N_4_O [M + H]^+^: 367.1553; Found: 367.1541.

1-Methyl-2-(4-*p*-tolyl-2*H*-1,2,3-triazol-2-yl)-1*H*-indole **3f**. Obtained as a white solid in 59% yield; m.p. 94–96 °C; ^1^H-NMR (400 MHz, CDCl_3_) δ 8.09 (s, 1H), 7.78 (d, *J* = 8.1 Hz, 2H), 7.66 (d, *J* = 7.9 Hz, 1H), 7.38 (d, *J* = 8.2 Hz, 1H), 7.34–7.25 (m, 3H), 7.19 (t, *J* = 7.0 Hz, 1H), 6.83 (s, 1H), 3.87 (s, 3H), 2.40 (s, 3H); ^13^C-NMR (101 MHz, CDCl_3_) δ 149.21, 139.17, 135.89, 135.48, 132.85, 129.76, 126.76, 126.15, 122.88, 121.36, 120.65, 109.79, 96.18, 30.87, 21.44; IR (neat) 3056, 3032, 2931, 1559, 1467, 1331, 1145, 966, 772, 701, 536, 458cm^−1^; HRMS(ESI) *m*/*z* calcd. for C_18_H_16_N_4_, [M + H]^+^ 289.1448, found 289.1439.

2-(4-(4-Butylphenyl)-2*H*-1,2,3-triazol-2-yl)-1-methyl-1*H*-indole **3g**. Obtained as a white solid in 52% yield; m.p. 87–89 °C; ^1^H-NMR (400 MHz, CDCl_3_) δ 8.07 (s, 1H), 7.78 (d, *J* = 8.1 Hz, 2H), 7.65 (d, *J* = 7.9 Hz, 1H), 7.35 (d, *J* = 8.1 Hz, 1H), 7.27 (t, *J* = 7.8 Hz, 3H), 7.17 (t, *J* = 7.4 Hz, 1H), 6.83 (s, 1H), 3.85 (s, 3H), 2.69–2.60 (m, 2H), 1.69–1.56 (m, 2H), 1.43–1.30 (m, 2H), 0.93 (t, *J* = 7.3 Hz, 3H); ^13^C-NMR (101 MHz, CDCl_3_) δ 149.48, 144.22, 135.94, 135.54, 132.88, 129.15, 127.13, 126.20, 122.91, 121.38, 120.68, 109.81, 96.18, 35.58, 33.59, 30.89, 22.42, 14.06; IR (neat) 3059, 3028, 1594, 1523, 1336, 988, 956, 845, 747, 526, 412 cm^−1^; HRMS(ESI) *m*/*z* calcd. for C_21_H_22_N_4_, [M + H]^+^ 331.1917, found 331.1909.

2-(4-(4-Methoxyphenyl)-2*H*-1,2,3-triazol-2-yl)-1-methyl-1*H*-indole **3h**. Obtained as a white solid in 60% yield; m.p. 92–93 °C; ^1^H-NMR (400 MHz, CDCl_3_) δ 8.05 (s, 1H), 7.82 (d, *J* = 8.8 Hz, 2H), 7.66 (d, *J* = 7.9 Hz, 1H), 7.38 (d, *J* = 8.2 Hz, 1H), 7.31 (t, *J* = 7.6 Hz, 1H), 7.18 (t, *J* = 7.4 Hz, 1H), 6.99 (d, *J* = 8.8 Hz, 2H), 6.83 (s, 1H), 3.87 (s, 3H), 3.84 (s, 3H); ^13^C-NMR (101 MHz, CDCl_3_) δ 160.26, 149.09, 135.86, 135.49, 132.51, 127.57, 126.12, 122.82, 122.18, 121.30, 120.60, 114.44, 109.50, 95.96, 55.37, 30.82; IR (neat) 3061, 2986, 1628, 1495, 1441, 1276, 1063, 987, 823, 715, 681, 512cm^−1^; HRMS(ESI) *m*/*z* calcd. for C_18_H_16_N_4_O, [M + H]^+^ 305.1397, found 305.1387.

1-Benzyl-2-(4-(4-ethynylphenyl)-2*H*-1,2,3-triazol-2-yl)-1*H*-indole **3i**. Obtained as a white solid in 53% yield; m.p. 108–110 °C; ^1^H-NMR (400 MHz, CDCl_3_) δ 8.06 (s, 1H), 7.75 (d, *J* = 8.0 Hz, 2H), 7.69 (d, *J* = 7.7 Hz, 1H), 7.55 (d, *J* = 8.0 Hz, 2H), 7.32 (d, *J* = 8.1 Hz, 1H), 7.26–7.15 (m, 5H), 7.05 (d, *J* = 7.2 Hz, 2H), 6.93 (s, 1H), 5.60 (s, 2H), 3.15 (s, 1H); ^13^C-NMR (101 MHz, CDCl_3_) δ 148.36, 137.30, 135.74, 135.14, 133.11, 132.76, 129.83, 128.62, 127.38, 126.45, 126.30, 126.02, 123.31, 122.74, 121.51, 121.01, 110.51, 97.00, 83.26, 78.53, 47.67; IR (neat) 3356, 3069, 3027, 2109, 1612, 1574, 1452, 1326, 956, 811, 707, 652, 485 cm^−1^; HRMS(ESI) *m*/*z* calcd. for C_25_H_18_N_4_, [M + H]^+^ 375.1604, found 375.1593.

2-(4-(3-Methoxyphenyl)-2*H*-1,2,3-triazol-2-yl)-1-methyl-1*H*-indole **3j**. Obtained as a white solid in 58% yield; m.p. 102–104 °C; ^1^H-NMR (400 MHz, CDCl_3_) δ 8.05 (s, 1H), 7.82 (d, *J* = 8.3 Hz, 2H), 7.66 (d, *J* = 7.8 Hz, 1H), 7.37 (d, *J* = 8.1 Hz, 1H), 7.30 (t, *J* = 7.5 Hz, 1H), 7.18 (t, *J* = 7.3 Hz, 1H), 6.99 (d, *J* = 8.3 Hz, 2H), 6.83 (s, 1H), 3.87 (s, 3H), 3.84 (s, 3H); ^13^C-NMR (101 MHz, CDCl_3_) δ 160.33, 149.09, 135.85, 135.48, 132.51, 127.56, 126.11, 122.82, 122.18, 121.30, 120.60, 114.20, 109.73, 96.08, 55.37, 30.70; IR (neat) 3059, 2923, 1619, 1486, 1442, 1253, 1072, 986, 836 cm^−1^; HRMS(ESI) *m*/*z* calcd. for C_18_H_16_N_4_O, [M + H]^+^ 305.1397, found 305.1390.

2-(4-(3-Chlorophenyl)-2*H*-1,2,3-triazol-2-yl)-1-methyl-1*H*-indole **3k**. Obtained as a white solid in 65% yield; m.p. 88–90 °C; ^1^H-NMR (400 MHz, CDCl_3_) δ 8.04 (s, 1H), 7.86 (s, 1H), 7.69 (td, *J* = 4.0, 1.5 Hz, 1H), 7.64 (d, *J* = 7.9 Hz, 1H), 7.36–7.26 (m, 4H), 7.17 (t, *J* = 6.7 Hz, 1H), 6.82 (s, 1H), 3.82 (s, 3H); ^13^C-NMR (101 MHz, CDCl_3_) δ 147.79, 135.83, 135.11, 134.93, 132.89, 131.24, 130.21, 128.96, 126.14, 125.93, 124.17, 122.94, 121.30, 120.66, 109.73, 96.19, 30.81; IR (neat) 3062, 3016, 1703, 1561, 1467, 1352, 1266, 1056, 956, 796, 737, 628 cm^−1^; HRMS(ESI) *m*/*z* calcd. for C_17_H_13_ClN_4_, [M + H]^+^ 309.0902, found 309.0893.

2-(4-(3-Fluorophenyl)-2*H*-1,2,3-triazol-2-yl)-1-methyl-1*H*-indole **3l**. Obtained as a white solid in 62% yield; m.p. 80–82 °C; ^1^H-NMR (400 MHz, CDCl_3_) δ 8.11 (s, 1H), 7.66 (t, *J* = 7.7 Hz, 2H), 7.61 (dd, *J* = 9.6, 2.0 Hz, 1H), 7.46–7.36 (m, 2H), 7.32 (t, *J* = 7.6 Hz, 1H), 7.19 (t, *J* = 7.4 Hz, 1H), 7.09 (td, *J* = 8.4, 2.5 Hz, 1H), 6.85 (s, 1H), 3.88 (s, 3H); ^13^C-NMR (101 MHz, CDCl_3_) δ 164.45, 162.00, 148.14, 135.92, 135.21, 133.03, 131.70 (d, *J* = 8.4 Hz), 130.67 (d, *J* = 8.3 Hz), 126.03, 123.03, 121.85 (d, *J* = 2.4 Hz), 121.40, 120.73, 116.96 (d, *J* = 21.3 Hz), 113.16 (d, *J* = 23.0 Hz), 109.79, 96.35, 30.87; IR (neat) 3059, 3036, 1601, 1553, 1492, 1352, 1231, 1142, 956, 843, 737, 628, 503 cm^−1^; HRMS(ESI) *m*/*z* calcd. for C_17_H_13_FN_4_, [M + H]^+^ 293.1197, found 293.1191.

2-(2-(1-Methyl-1*H*-indol-2-yl)-2*H*-1,2,3-triazol-4-yl)phenol **3m**. Obtained as a white solid in 47% yield; m.p. 132–135 °C; ^1^H-NMR (400 MHz, CDCl_3_) δ 9.46 (s, 1H), 8.25 (s, 1H), 7.69 (d, *J* = 7.6 Hz, 2H), 7.41 (d, *J* = 8.2 Hz, 1H), 7.38–7.30 (m, 2H), 7.22 (t, *J* = 7.4 Hz, 1H), 7.11 (d, *J* = 8.2 Hz, 1H), 7.02 (t, *J* = 7.5 Hz, 1H), 6.85 (s, 1H), 3.86 (s, 3H); ^1^H-NMR (400 MHz, CDCl_3_): δ 9.47 (s, 1H), 8.26 (s, 1H), 7.69 (d, *J* = 8.0 Hz, 2H), 7.42 (d, *J* = 8.2 Hz, 1H), 7.38–7.33 (m, 2H), 7.23 (t, *J* = 7.4 Hz, 1H), 7.11 (d, *J* = 8.3 Hz, 1H), 7.03 (t, *J* = 7.4 Hz, 1H) 6.85 (s, 1H), 3.87 (s, 3H); ^13^C-NMR (101 MHz, CDCl_3_) δ 155.42, 154.18, 135.86, 132.78, 130.95, 126.66, 123.34, 121.43, 120.92, 120.08, 117.63, 109.86, 96.80, 88.55, 86.71, 30.82; IR (neat) 3108, 3049, 2986, 1663, 1564, 1474, 1366, 996, 757, 727, 691 cm^−1^; HRMS(ESI) *m*/*z* calcd. for C_17_H_14_N_4_O, [M + H]^+^ 291.1240, found 291.1236.

1-Methyl-2-(4-(thiophen-3-yl)-2*H*-1,2,3-triazol-2-yl)-1*H*-indole **3n**. Obtained as a white solid in 51% yield; m.p. 92–94 °C; ^1^H-NMR (400 MHz, CDCl_3_) δ 7.97 (s, 1H), 7.70 (d, *J* = 2.0 Hz, 1H), 7.64 (d, *J* = 7.9 Hz, 1H), 7.51 (d, *J* = 4.8 Hz, 1H), 7.39–7.36 (m, 1H), 7.34 (d, *J* = 8.2 Hz, 1H), 7.28 (t, *J* = 7.5 Hz, 1H), 7.17 (t, *J* = 7.3 Hz, 1H), 6.82 (s, 1H), 3.82 (s, 3H); ^13^C-NMR (101 MHz, CDCl_3_) δ 145.31, 135.82, 135.26, 133.09, 130.85, 126.73, 126.03, 125.91, 122.87, 122.61, 121.30, 120.61, 109.72, 96.24, 30.44; IR (neat) 3033, 2932, 1586, 1454, 1336, 1189, 965, 873, 769, 715; HRMS(ESI) *m*/*z* calcd. for C_15_H_12_N_4_S, [M + H]^+^ 281.0855, found 281.0847.

2-(1-Methyl-1*H*-indol-2-yl)-2*H*-benzo[*d*][1,2,3]triazole **3o**. Obtained as a white solid in 54% yield; m.p. 137–139 °C; ^1^H-NMR (400 MHz, CDCl_3_) δ 7.95 (dd, *J* = 6.6, 3.1 Hz, 2H), 7.70 (d, *J* = 7.9 Hz, 1H), 7.50–7.38 (m, 3H), 7.34 (t, *J* = 7.5 Hz, 1H), 7.21 (t, *J* = 7.1 Hz, 1H), 7.06 (s, 1H), 4.00 (s, 3H); ^13^C-NMR (101 MHz, CDCl_3_) δ 144.93, 136.51, 135.80, 127.48, 126.06, 123.41, 121.59, 120.89, 118.31, 109.79, 97.65, 31.44; IR (neat) 3052, 3028, 1568, 1452, 1346, 1271, 1059, 956, 771, 732, 692 cm^−1^; HRMS(ESI) *m*/*z* calcd. for C_15_H_12_N_4_, [M + H]^+^ 249.1135, found 249.1124.

1-Benzyl-2-(4-bromo-5-phenyl-2*H*-1,2,3-triazol-2-yl)-1*H*-indole **3p**. Obtained as a white solid in 53% yield; m.p. 90–91 °C; ^1^H-NMR (400 MHz, CDCl_3_) δ 7.92 (d, *J* = 6.5 Hz, 2H), 7.70 (d, *J* = 7.8 Hz, 1H), 7.50–7.40 (m, 3H), 7.34 (d, *J* = 8.1 Hz, 1H), 7.28 (d, *J* = 7.0 Hz, 1H), 7.24–7.17 (m, 4H), 7.05 (d, *J* = 6.6 Hz, 2H), 6.93 (s, 1H), 5.61 (s, 2H); ^13^C-NMR (101 MHz, CDCl_3_) δ 146.99, 137.07, 135.75, 134.54, 129.39, 128.71, 128.64, 128.32, 127.50, 127.45, 126.77, 126.51, 126.11, 123.52, 121.62, 121.08, 110.51, 97.33, 47.65; IR (neat) 3059, 3030, 2924, 1558, 1454, 1328, 1161, 1010, 956, 727, 694cm^−1^; HRMS (ESI) Calcd. for C_23_H_18_BrN_4_ [M + H]^+^: 429.07094; Found: 429.06996.

1-Benzyl-2-(4-butyl-2*H*-1,2,3-triazol-2-yl)-1*H*-indole **3q** [41]. Obtained as a colorless oil in 60% yield; ^1^H-NMR (400 MHz, CDCl_3_) δ 7.59 (d, *J* = 7.7 Hz, 1H), 7.51 (s, 1H), 7.22 (d, *J* = 8.0 Hz, 1H), 7.18–7.05 (m, 5H), 6.94 (d, *J* = 6.9 Hz, 2H), 6.74 (s, 1H), 5.46 (s, 2H), 2.66 (t, *J* = 7.7 Hz, 2H), 1.83-1.46 (m, 2H), 1.36–1.20 (m, 2H), 0.85 (t, *J* = 7.3 Hz, 3H); ^13^C-NMR (101 MHz, CDCl_3_) δ 150.56, 137.32, 135.46, 134.87, 128.54, 127.28, 126.49, 126.38, 122.96, 121.37, 120.77, 110.44, 96.66, 47.42, 31.09, 25.14, 22.15, 13.75.

1-Benzyl-2-(2*H*-1,2,3-triazol-2-yl)-1*H*-indole **3r** [41]. Obtained as a white solid in 95% yield; m.p. 95–98 °C; ^1^H-NMR (400 MHz, CDCl_3_) δ 7.76 (s, 2H), 7.61 (d, *J* = 7.7 Hz, 1H), 7.22 (d, *J* = 8.2 Hz, 1H), 7.17–7.07 (m, 5H), 6.94 (d, *J* = 6.7 Hz, 2H), 6.80 (s, 1H), 5.45 (s, 2H); ^13^C-NMR (101 MHz, CDCl_3_) δ 137.16, 135.97, 135.51, 135.14, 128.69, 127.37, 126.43, 126.25, 123.24, 121.58, 120.92, 110.43, 97.20, 47.57.

5-Fluoro-2-(4-phenyl-2*H*-1,2,3-triazol-2-yl)-1*H*-indole **3s** [41]. Obtained as a white solid in 78% yield; m.p. 116–119 °C; ^1^H-NMR (400 MHz, CDCl_3_) δ 8.08 (s, 1H), 7.81 (d, *J* = 7.0 Hz, 2H), 7.48–7.38 (m, 3H), 7.33 (dd, *J* = 9.2, 2.4 Hz, 1H), 7.26–7.18 (m, 4H), 7.06 (d, *J* = 6.6 Hz, 2H), 6.98 (td, *J* = 9.1, 2.5 Hz, 1H), 6.89 (s, 1H), 5.61 (s, 2H); ^13^C-NMR (101 MHz, CDCl_3_) δ 158.46(d, *J = *235.9 Hz), 149.40, 137.06, 136.43, 133.17, 132.19, 129.38, 129.19, 129.04, 128.69, 127.50, 126.70, 126.59, 126.45, 126.22, 111.84, 111.53 (d, *J* = 9.5 Hz), 111.39, 106.24(d, *J = *23.6 Hz), 96.72(d, *J = *4.5 Hz), 96.69, 47.85.

1-Benzyl-2-(4-phenyl-2*H*-1,2,3-triazol-2-yl)-1H-indol-5-yl-4-methylbenzenesulfonate **3t** [41]. Obtained as a white solid in 83% yield; m.p. 169–171 °C; ^1^H-NMR (400 MHz, CDCl_3_) δ 8.07 (s, 1H), 7.80 (d, *J* = 6.9 Hz, 2H), 7.70 (d, *J* = 8.3 Hz, 2H), 7.48–7.36 (m, 3H), 7.28 (d, *J* = 8.1 Hz, 2H), 7.25–7.18 (m, 5H), 7.04 (d, *J* = 6.3 Hz, 2H), 6.89 (dd, *J* = 8.9, 2.3 Hz, 1H), 6.84 (s, 1H), 5.58 (s, 2H), 2.43 (s, 3H); ^13^C-NMR (101 MHz, CDCl_3_) δ 149.50, 145.15, 144.25, 136.78, 136.42, 133.98, 133.28, 132.48, 129.71, 129.26, 129.06, 128.70, 128.64, 127.58, 126.50, 126.22, 123.62, 118.06, 114.65, 111.24, 97.07, 47.96, 21.76.

*N*-(1-Benzyl-2-(4-phenyl-2*H*-1,2,3,-triazol-2-yl)-1*H*-indol-5-yl)acetamide **3u** [41]. Obtained as a white solid in 60% yield; m.p. 128–131 °C; ^1^H-NMR (400 MHz, CDCl_3_) δ 8.06 (s, 1H), 7.87 (br, 1H), 7.80 (d, *J* = 7.2 Hz, 2H), 7.51–7.36 (m, 4H), 7.30–7.15 (m, 5H), 7.04 (d, *J* = 6.8 Hz, 2H), 6.87 (s, 1H), 5.58 (s, 2H), 2.17 (s, 3H); ^13^C-NMR (101 MHz, CDCl_3_) δ 168.44, 149.27, 137.20, 135.88, 133.04, 131.50, 129.43, 129.13, 129.02, 128.63, 127.40, 126.46, 126.21, 117.51, 113.23, 110.78, 96.88, 47.77, 24.49.

General procedure for coupling reaction (condition 2) for the synthesis of **3v**–**3y**: To a suspension of **1v** (0.1 mmol), **2a** (0.2 mmol) in dry dioxane (1 mL), was added 0.1 mL CHCl_3_, and then added dropwise a solution of *N*-iodosuccinimide (0.3 mmol) in dioxane (1 mL) in 5 min. 30 min later, the reaction mixture was diluted with 20 mL EtOAc, and was washed with saturated aqueous Na_2_S_2_O_3_ (5 mL), brine (10 mL) and water (10 mL). The organic phase was dried over anhydrous Na_2_SO_4_, filtered and concentrated in vacuo, purification of the crude product through flash chromatography (petroleum/EtOAc = 50/1 as the eluent) afforded **3v** as a white solid.

3-Methyl-2-(4-phenyl-2*H*-1,2,3,-triazol-2-yl)-1*H*-indole **3v** [41]. Obtained as a white solid in 55% yield; m.p. 132–134 °C; ^1^H-NMR (400 MHz, CDCl_3_) δ 8.88 (br, 1H), 8.03 (s, 1H), 7.86 (d, *J* = 5.3 Hz, 2H), 7.60 (d, *J* = 7.8 Hz, 1H), 7.45 (t, *J* = 7.4 Hz, 2H), 7.41–7.35 (m, 1H), 7.31 (d, *J* = 8.0 Hz, 1H), 7.23 (t, *J* = 7.0 Hz, 1H), 7.17 (t, *J* = 7.0 Hz, 1H), 2.66 (s, 3H); ^13^C-NMR (101 MHz, CDCl_3_) δ 148.41, 132.86, 132.15, 130.67, 129.67, 129.14, 129.05, 129.02, 126.17, 123.07, 120.22, 119.38, 110.88, 100.67, 9.02.

1-Benzyl-3-methyl-2-(4-phenyl-2*H*-1,2,3,-triazol-2-yl)-1*H*-indole **3w** [41]. Obtained as a white solid in 75% yield; m.p. 78–80 °C; ^1^H-NMR (400 MHz, CDCl_3_) δ 8.09 (s, 1H), 7.81 (d, *J* = 7.0 Hz, 2H), 7.66 (d, *J* = 7.8 Hz, 1H), 7.43 (t, *J* = 7.3 Hz, 2H), 7.40–7.34 (m, 1H), 7.30–7.23 (m, 2H), 7.21–7.13 (m, 4H), 7.03 (d, *J* = 6.4 Hz, 2H), 5.26 (s, 2H), 2.36 (s, 3H); ^13^C-NMR (101 MHz, CDCl_3_) δ 149.24, 137.35, 134.70, 133.01, 131.66, 129.69, 129.09, 129.07, 128.60, 127.39, 126.90, 126.68, 126.26, 123.67, 120.17, 119.99, 110.33, 107.43, 47.18, 8.39.

Methyl 2-(1-benzyl-2-(4-phenyl-2*H*-1,2,3,-triazol-2-yl)-1*H*-indol-3-yl)acetate **3x** [41]. Obtained as a white solid in 67% yield; m.p. 104–106 °C; ^1^H-NMR (400 MHz, CDCl_3_) δ 8.11 (s, 1H), 7.82 (d, *J* = 7.0 Hz, 2H), 7.69 (d, *J* = 7.8 Hz, 1H), 7.45 (t, *J* = 7.3 Hz, 2H), 7.42–7.37 (m, 1H), 7.33–7.27 (m, 2H), 7.24–7.18 (m, 4H), 7.06 (d, *J* = 6.6 Hz, 2H), 5.42 (s, 2H), 3.91 (s, 2H), 3.63 (s, 3H); ^13^C-NMR (101 MHz, CDCl_3_) δ 171.49, 149.37, 137.06, 134.65, 133.19, 132.61, 129.49, 129.14, 129.03, 128.61, 127.41, 126.62, 126.23, 123.76, 120.82, 119.90, 110.55, 103.39, 52.09, 47.46, 29.79.

(*S*)-Methyl 2-acetamido-3-(2-(4-phenyl-2*H*-1,2,3,-triazol-2-yl)-1*H*-indol-3-yl) propanoate **3y** [41]. Obtained as a yellow solid in 62% yield, ${\left[\alpha \right]}_{D}^{20}$ = +15.1 (*c *= 1.00); m.p. 137–140 °C; ^1^H-NMR (400 MHz, DMSO) δ 12.10 (br, 1H), 8.74 (s, 1H), 8.47 (d, *J = *7.6 Hz, 1H), 8.06 (d, *J* = 7.2 Hz, 2H), 7.64 (d, *J* = 7.9 Hz, 1H), 7.56 (t, *J* = 7.5 Hz, 2H), 7.50–7.42 (m, 2H), 7.20 (t, *J* = 7.5 Hz, 1H), 7.11 (t, *J* = 7.4 Hz, 1H), 4.69 (q, *J* = 7.5 Hz, 1H), 3.64 (dd, *J = *13.9, 7.4 Hz, 1H), 3.44 (s, 3H), 3.37 (dd, *J* = 13.9, 7.5 Hz, 1H), 1.77 (s, 3H); ^13^C-NMR (101 MHz, DMSO) δ 172.82, 169.65, 148.85, 133.76, 133.60, 131.78, 129.63, 129.58, 128.12, 126.49, 125.90, 122.93, 120.23, 119.27, 112.15, 100.02, 53.34, 52.10, 26.63, 22.76.

Synthetic procedure for compound **5**: A solution of **3v** (0.05 mmol) and Pd(OAc)_2_ (0.05 mmol) in dry CH_2_Cl_2_ (1 mL) was stirred at room temperature for 2 h. After filtration, slow evaporation of the resulting solution gave a colorless crystal **5**.

Compound **5**. Obtained as a yellow solid in 45% yield, ^1^H-NMR (400 MHz, CDCl_3_) δ 7.43–7.38 (m, 1H), 7.36 (dt, *J = *7.8, 3.8 Hz, 1H), 7.01–6.94 (m, 1H), 6.72 (d, *J = *8.0 Hz, 1H), 6.61 (ddd, *J = *7.9, 5.2, 2.7 Hz, 1H), 6.57 (s, 1H), 2.38 (s, 1H), 2.30 (s, 1H); ^13^C-NMR (101 MHz, CDCl_3_) δ 193.64, 186.49, 180.98, 173.69, 161.51, 129.30, 128.48, 126.61, 126.02, 121.68, 119.74, 117.96, 111.80, 98.63, 23.79, 7.65.4.

## 4. Conclusions

In conclusion, *N-*2-indolyl-triazoles (NITs) are a novel class of effective UV/blue-light-emitting dyes. Through a comprehensive comparison of the substituted groups on the indole nitrogen atom, the indole group, and the triazole group, a general trend was revealed regarding how to effectively adjust the photoactivity of these compounds. Both emission wavelengths (410–470 nm) and Stokes shifts (89–143 nm) could be adjusted with various substituted functional groups. We hope that further derivatizations of these NIT molecules would gave highly efficient UV-emitting biocompatible amino acid NIT probes, thus supporting the potential use of these novel dyes in related chemical, material, and biological applications.

## Figures and Tables

**Table 1:**
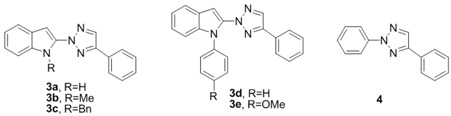
Fluorescence emission behavior of compounds with different Indole *N*-substitutions ^(a)^.

	Absorption (nm)	Emission (λ_max_) (nm)	Φ ^(b)^	Fluorescence Intensity (×10^3^)	Stokes Shift
**4**	292 (0.238)	345	0.39	46.3	53
**3a**	325 (0.246)	429	0.65	28.20	104
**3b**	310 (0.238)	437	0.90	128.77	127
**3c**	315 (0.219)	432	0.83	105.02	117
**3d**	298 (0.312)	422	0.97	136.94	124
**3e**	298 (0.258)	423	0.99	109.48	125

^(a)^ Sample information: 1.0 × 10^−5^ mol L^−1^ in dichloromethane (DCM), excitation at 320 nm with 2.5 nm slit. ^(b)^ Quantum yields (Φ) were determined based on 1.0 × 10^−5^ mol L^−1^ 9,10-Diphenylanthracene in cyclohexane (Ф = 0.95).

**Table 2:**
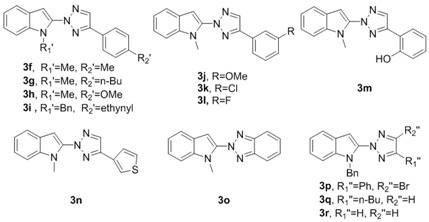
Fluorescence emission behavior of compounds with different triazole-substitutions ^(a)^.

	Absorption (nm)	Emission (λ_max_) (nm)	Φ ^(b)^	Fluorescence Intensity (×10^3^)	Stokes Shift
**3f**	308 (0.302)	427	0.71	134.75	119
**3g**	310 (0.337)	434	0.46	95.14	124
**3h**	315 (0.306)	432	0.44	88.16	117
**3i**	315 (0.347)	443	0.62	144.93	128
**3j**	315 (0.334)	431	0.73	160.55	116
**3k**	310 (0.379)	440	0.80	191.11	130
**3l**	306 (0.346)	447	0.67	142.66	141
**3m**	310 (0.263)	445	0.58	97.74	135
**3n**	305 (0.275)	430	0.69	113.77	125
**3o**	340 (0.346)	467	0.14	29.77	127
**3p**	295 (0.259)	438	0.20	24.8	143
**3q**	295 (0.173)	414	0.55	25.78	119
**3r**	294 (0.274)	415	0.84	60.6	121

^(a)^ Sample information: 1.0 × 10^−5^ mol L^−1^ in DCM, excitation at 320 nm with 2.5 nm slit. ^(b)^ Quantum yields (Φ) were determined based on 1.0 × 10^−5^ mol L^−1^ 9,10-Diphenylanthracene in cyclohexane (Ф = 0.95).

**Table 3:**
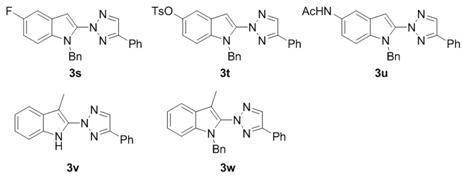
Fluorescence emission behavior of compounds with different indole-substitutions ^(a)^.

	Absorption (nm)	Emission (λmax) (nm)	Φ ^(b)^	Fluorescence Intensity (×10^3^)	Stokes Shift
**3s**	304(0.431)	419	0.76	165.15	115
**3t**	310(0.336)	427	0.73	163.18	117
**3u**	338(0.126)	427	0.85	61.62	89
**3v**	330(0.323)	426	0.69	143.63	96
**3w**	299(0.340)	424	0.86	117.16	125

^(a)^ Sample information: 1.0 × 10^−5^ mol L^−1^ in DCM, excitation at 320 nm with 2.5 nm slit. ^(b)^ Quantum yields (Φ) were determined based on 1.0 × 10^−5^ mol L^−1^ 9,10-Diphenylanthracene in cyclohexane (Ф = 0.95).

**Table 4:**
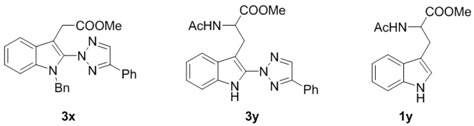
Fluorescence emission behavior of compounds **3x** and **3y** ^(a)^.

	Absorption (nm)	Emission (λmax) (nm)	Φ ^(b)^	Fluorescence Intensity (×10^3^)	Stokes Shift
**3x**	328(0.373)	418	0.72	173.80	90
**3y**	326(0.409)	430	0.60	165.49	104

^(a)^ Sample information: 1.0 × 10^−5^ mol L^−1^ in DCM, excitation at 320 nm with 2.5 nm slit. ^(b)^ Quantum yields (Φ) were determined based on 1.0 × 10^−5^ mol L^−1^ 9,10-Diphenylanthracene in cyclohexane (Ф = 0.95).

## Supplementary Materials

A brief experimental details, and spectral data for all new products and NOESY spectra for compound **3b**, **3h,** and **5** are available online.
