# Supplementary material for: Study on the Fluorescent Activity of N2-Indolyl-1,2,3-triazole
Source: Molecules. 2017 Sep 5;22(9):1380. doi: 10.3390/molecules22091380 (PMC6151483; doi:10.3390/molecules22091380)
Supplement: Supplementary file 1 [file molecules-22-01380-s001.pdf]

Supporting Information for

Study on N^2-Indolyl-1,2,3-triazole's Fluorescent Activity

You-Can Zhang, Rui Jin, Luo-Yuan Li, Zili Chen,* Li-Min Fu*

Contents

General Conditions ……………………………………………………………...S2
General procedure for coupling reaction, condition I………………………….…...S2
General procedure for coupling reaction, condition II……………………….…….S3
Synthetic procedure for compound **5**……………………………………..…..S3
The absorption and emission spectra of indole-triazole conjugates..…………... S3
Characterization Data …………………………………………………….……….S16
^{1}H NMR and ^{13}C NMR spectra…………………………………………………S34

General Conditions

All reactions were run under an inert atmosphere (Ar) with flame-dried glassware using standard techniques for manipulating air-sensitive compounds. All solvents were dried and purified before use by standard procedures. Commercial reagents were used as supplied or purified by standard techniques where necessary. Column chromatography was performed using 200-300 mesh silica with the proper solvent system according to TLC analysis using $KMnO_4$ stain and UV light to visualize the reaction components. Unless otherwise noted, nuclear magnetic resonance spectra were recorded on 400 MHz spectrometer. NMR data were reported as follows: chemical shift, multiplicity (s = singlet, d = doublet, t = triplet, m = multiplet and bs = broad singlet), coupling constant in Hz and integration. Chemical shifts for ^{13}C NMR spectra were recorded in parts per million from tetramethylsilane using the central peak of deuterochloroform (77.05 ppm) as the internal standard. IR spectra were recorded on an FTIR spectrometer (KBr) and reported in reciprocal centimeters (cm^{-1}). HRMS data were obtained using ESI ionization. Mp data were measured with micro melting point apparatus. Electronic absorption spectra were obtained on a CARY50 PROBE UV-visible spectrometer; Photoluminescent spectra were recorded with a LS55 luminescence spectrometer with the excitation and emission slit widths at 2.5 nm.[1] The indoles **1c**,**1d**,**1e**,**1s-1u**,**1w**,**1x**, tryptophan **1y**, triazoles were prepared according to the reported procedures,[2-7] and 2,4-diphenyl-2H-1,2,3-triazole was prepared according to the reported procedures.[8] The indoles 1a,1b were bought from commercial source.

General procedure for coupling reaction, condition 1 for the synthesis of 3a-3u:

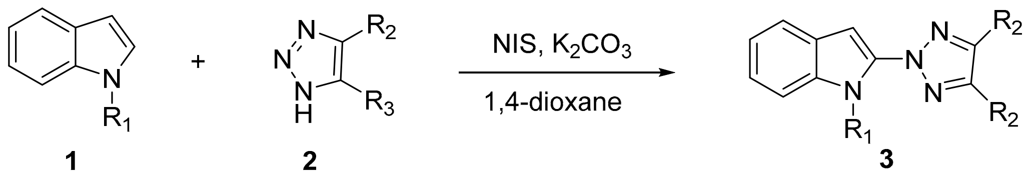

To a suspension of N-iodosuccinimide (0.3 mmol) and K_2CO_3 (0.5 mmol) in dry dioxane (1 mL), was added dropwise a solution of 1 (0.2 mmol) and 2 (0.1 mmol) in dioxane (1 mL) in 5 min. 30 min later, the reaction mixture was diluted with 20 mL EtOAc, and was then washed with saturated aqueous $Na_2S_2O_3$ (5 mL), brine (10 mL) and water (10 mL). The organic phase was dried over anhydrous sodium sulfate, filtered and concentrated in vacuo. Purification of the crude product through flash chromatography (petroleum/EtOAc=50/1 as the eluent) afforded **3**.

General procedure for coupling reaction, condition 2 for the synthesis of 3v-3y:

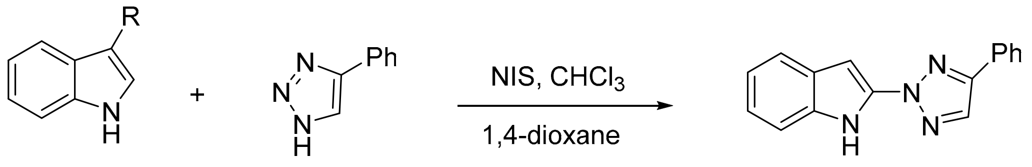

1 2a 3

To a suspension of 1 (0.1 mmol), 2a (0.2 mmol) in dry dioxane (1 mL), was added 0.1 mL $CHCl_3$, and then added dropwise a solution of N-iodosuccinimide (0.3 mmol) in dioxane (1 mL) in 5 min. 30 min later, the reaction mixture was diluted with 20 mL EtOAc, and was then washed with saturated aqueous $Na_2S_2O_3$ (5 mL), brine (10 mL) and water (10 mL). The organic phase was dried over anhydrous sodium sulfate, filtered and concentrated in vacuo. Purification of the crude product through flash chromatography (petroleum/EtOAc=50/1 as the eluent) afforded 3.

Synthetic procedure for compound 5:

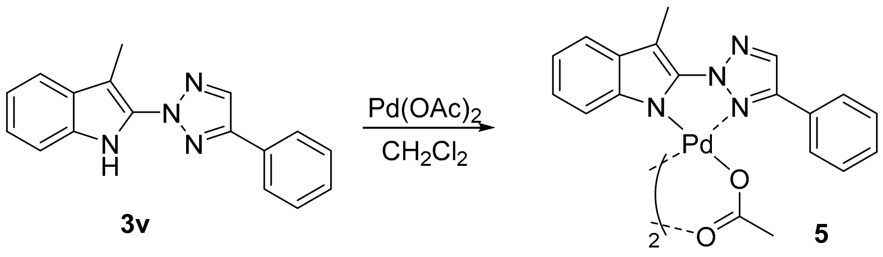

3v 5

To a suspension of 3v (0.05 mmol) and $Pd(OAc)_2$ (0.05 mmol) in dry CH_2Cl_2 (1 mL) at rt gave a white solid 5 in 45% yield.

The absorption and emission spectra of products[1]

For the method to determine the fluorescence quantum yield:

Fluorescence quantum yield was determined in DCM using optically matching solutions of 9,10-Diphenylanthracene (Φ_f= 0.95 in cyclohexane) as standard at an excitation wavelength of 320 nm and the quantum yield was calculated using the following equation:

$$\Phi_f = \Phi_r (A_r F_s / A_s F_r) (n_s^2 / n_r^2)$$

where, As and Ar are the absorbance of the sample and the reference, respectively, at the same excitation wavelength, Fs and Fr are the corresponding relative integrated fluorescence intensities, and n is the refractive index of the solvent.

The absorption and emission spectra of products **3(a-e),4**:

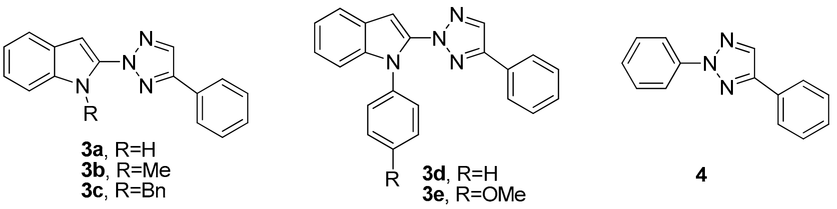

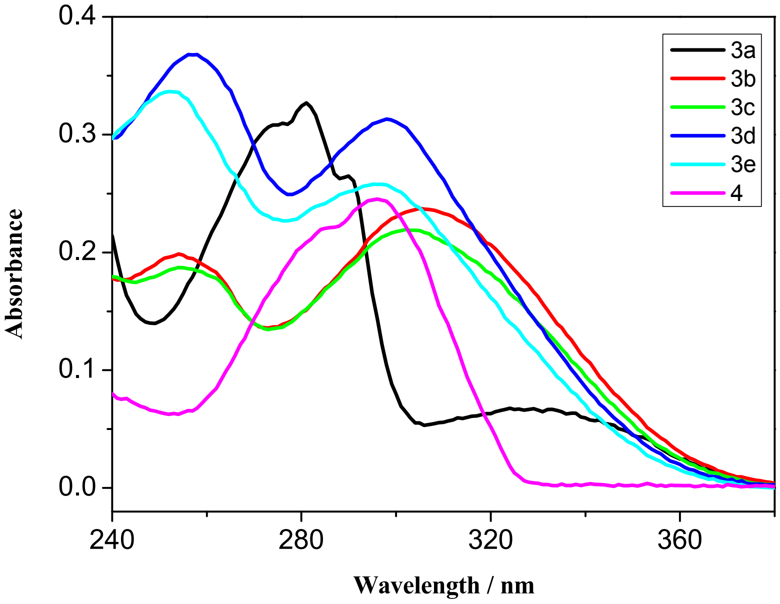

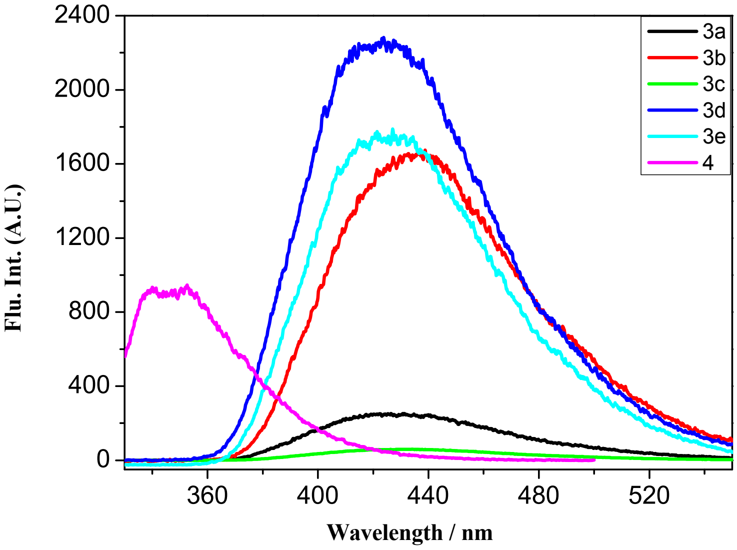

Sample preparation: 1.0 x 10^{-5} mol/L in CH_2Cl_2, with 2.5 nm slit.

The absorption and emission spectra of products **3(f-r)**:

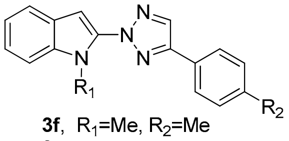

3f, R_1=Me, R_2=Me
3g, R_1=Me, R_2=n-Bu
3h, R_1=Me, R_2=OMe
3i , R_1=Bn, R_2=ethynyl

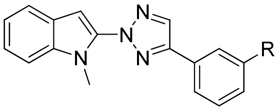

3j, R=OMe
3k, R=Cl
3l, R=F

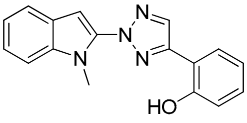

3m

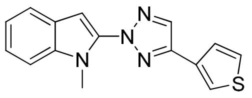

3n

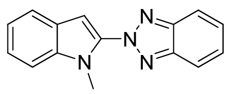

3o

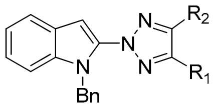

3p, R_1=Ph, R_2=Br
3q, R_1=n-Bu, R_2=H
3r, R_1=H, R_2=H

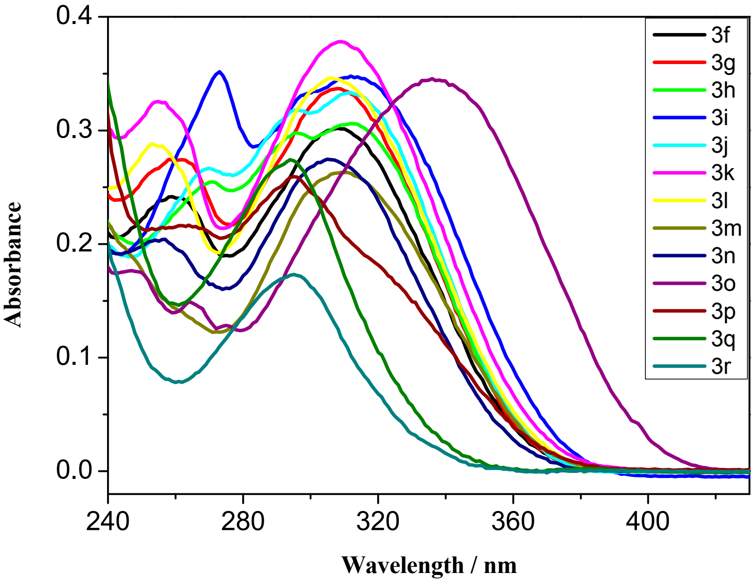

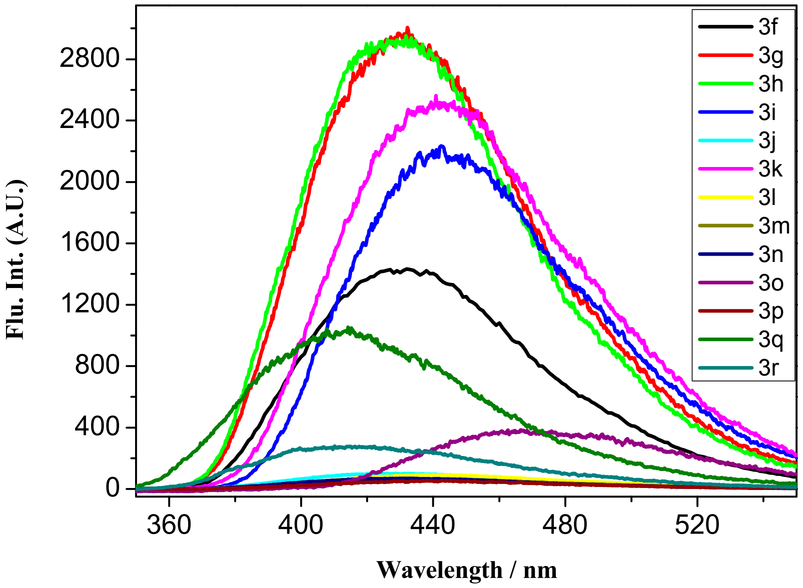

Sample preparation: 1.0 x 10^{-5} mol/L in CH_2Cl_2, with 2.5 nm slit.

The absorption and emission spectra of products **3(s-w)**:

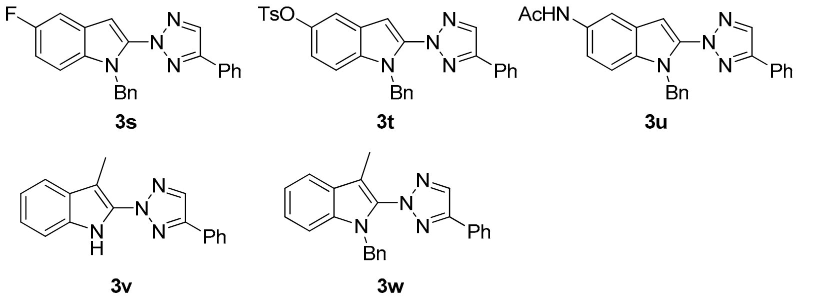

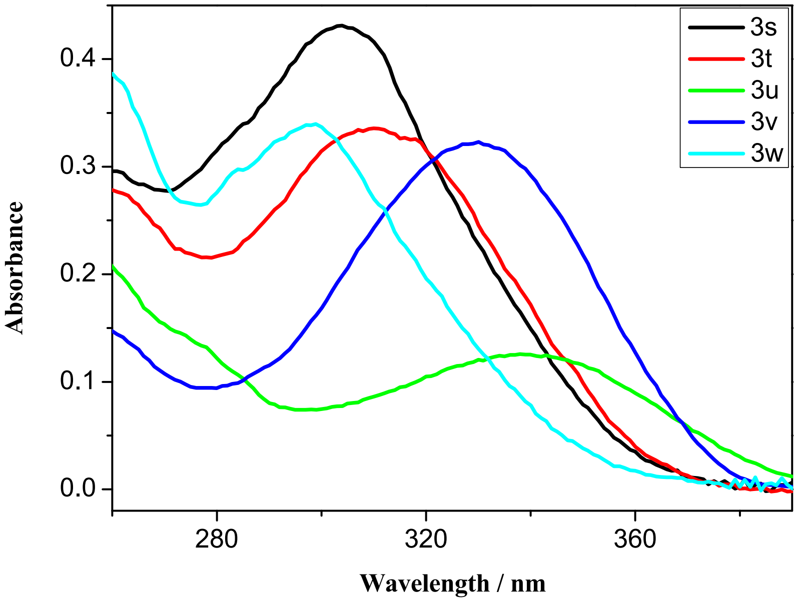

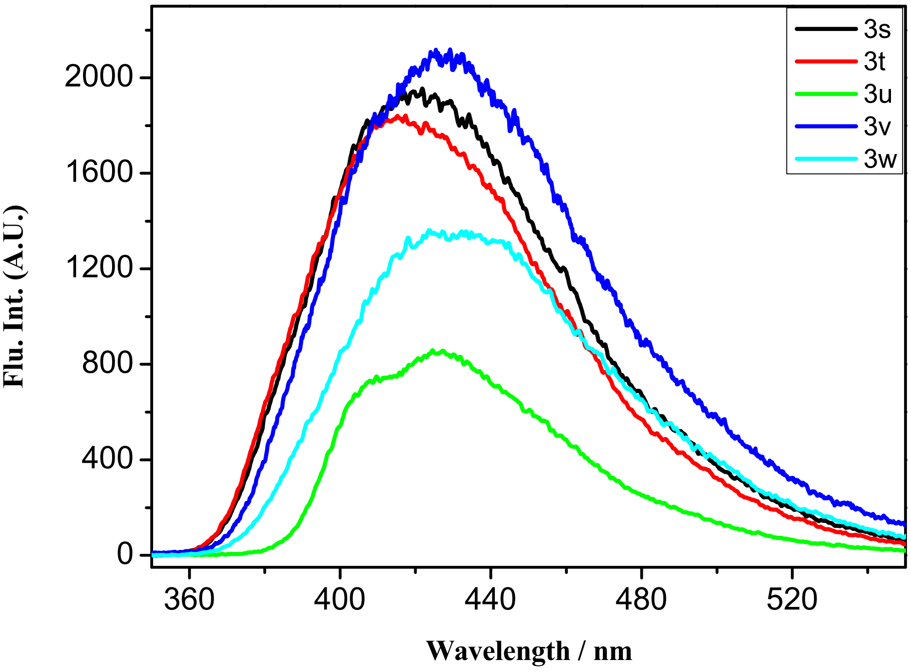

Sample preparation: 1.0 x 10^{-5} mol/L in CH_2Cl_2, with 2.5 nm slit.

The absorption and emission spectra of products **3(x-y)**

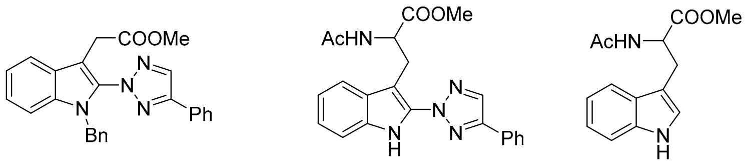

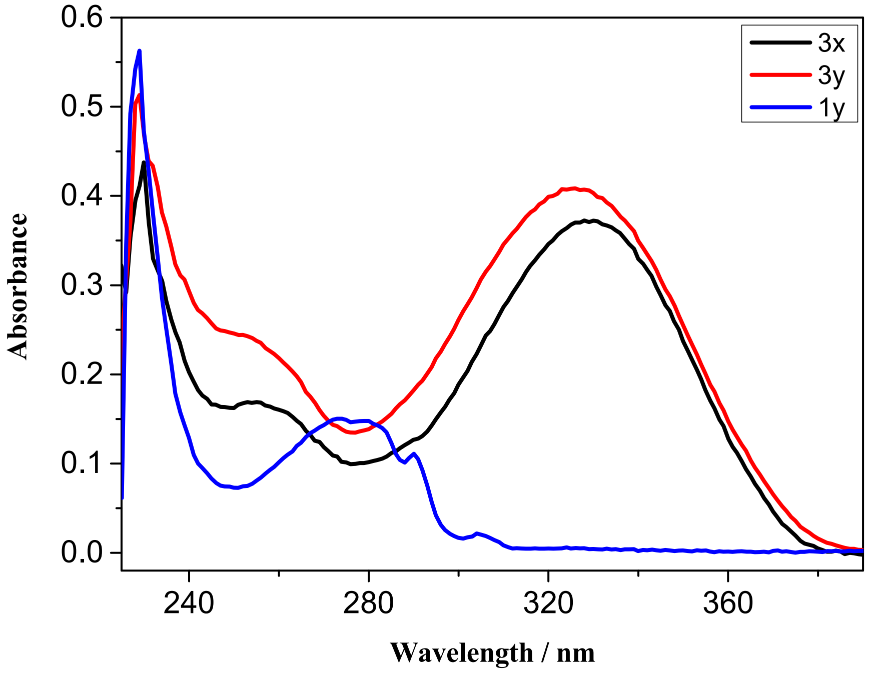

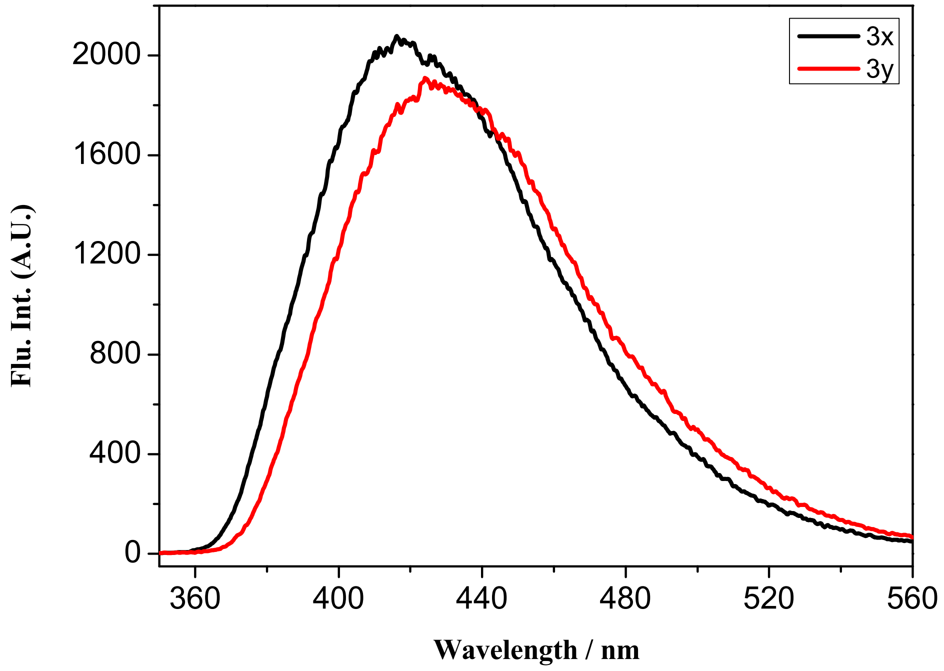

Sample preparation: 1.0 x 10^{-5} mol/L in CH_2Cl_2, with 2.5 nm slit.

Characterization Data:

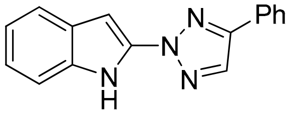

2-(4-phenyl-2H-1,2,3-triazol-2-yl)-1H-indole 3a :

Obtained as a white solid in 60.2% yield; M.p. 128 – 130 °C; ^{1}H NMR (400 MHz, $CDCl_3$) δ 9.07 (br, 1H), 8.07 (s, 1H), 7.89 (d, J = 7.1 Hz, 2H), 7.66 (d, J = 7.8 Hz, 1H), 7.49 (t, J = 7.4 Hz, 2H), 7.42 (dd, J = 7.7, 2.8 Hz, 2H), 7.28 – 7.22 (m, 1H), 7.21 – 7.15 (m, 1H), 6.90 (d, J = 1.4 Hz, 1H); ^{13}C NMR (101 MHz, $CDCl_3$) δ 149.05, 134.80, 133.54, 132.82, 129.44, 129.16, 129.06, 127.89, 126.22, 122.86, 121.04, 120.98, 111.20, 90.31; IR (neat) 3119, 3036, 2928, 1564, 1495, 1336, 1091, 956, 858, 767, 732, 686cm^{-1}; HRMS(ESI) m/z calcd for $C_{16}H_{12}N_4$, $[M+H]^+$ 261.1135, found 261.1126

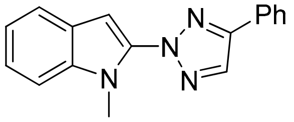

1-methyl-2-(4-phenyl-2H-1,2,3-triazol-2-yl)-1H-indole 3b

Obtained as a white solid in 63.8% yield; M.p. 90 – 91 °C; ^{1}H NMR (400 MHz, $CDCl_3$) δ 8.11 (s, 1H), 7.88 (d, J = 7.1 Hz, 2H), 7.66 (d, J = 7.9 Hz, 1H), 7.46 (t, J = 7.4 Hz, 2H), 7.38 (dd, J = 12.9, 7.8 Hz, 2H), 7.30 (t, J = 7.1 Hz, 1H), 7.18 (t, J = 6.9 Hz, 1H), 6.84 (s, 1H), 3.83 (s, 3H); ^{13}C NMR (101 MHz, $CDCl_3$) δ 149.08, 135.92, 135.44, 133.02, 129.61, 129.17, 129.10, 126.27, 122.96, 121.41, 120.71, 120.30, 109.83, 96.27, 30.56; IR (neat) 3061, 3035, 1587, 1457, 1326, 968, 832, 767, 701, 502cm^{-1}; HRMS(ESI) m/z calcd for $C_{17}H_{14}N_4$, $[M+H]^+$ 275.1291, found 275.1283

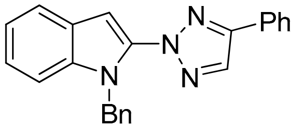

1-Benzyl-2-(4-phenyl-2*H*-1,2,3-triazol-2-yl)-1*H*-indole 3c :

Obtained as a white solid in 71.6% yield; M.p. 91 – 92 °C; ^{1}H NMR (400 MHz, $CDCl_3$) δ 8.01 (s, 1H), 7.77 (d, J = 7.2 Hz, 2H), 7.67 (d, J = 7.6 Hz, 1H), 7.39 (t, J = 7.3 Hz, 2H), 7.34 (d, J = 7.1 Hz, 1H), 7.28 (d, J = 8.1 Hz, 1H), 7.22 (d, J = 6.9 Hz, 1H), 7.20 – 7.11 (m, 4H), 7.04 (d, J = 7.0 Hz, 2H), 6.92 (s, 1H), 5.57 (s, 2H); ^{13}C NMR (101 MHz, $CDCl_3$) δ 149.31, 137.45, 135.77, 135.39, 133.09, 129.60, 129.18, 129.10, 128.70, 127.44, 126.60, 126.44, 126.29, 123.30, 121.57, 121.06, 110.62, 96.96, 47.71; IR (neat) 3059, 3032, 1564, 1454, 1346, 956, 767, 727, 692cm^{-1};HRMS (ESI) Calcd for $C_{23}H_{19}N_4$ $[M+H]^+$: 351.1604; Found: 351.1594.

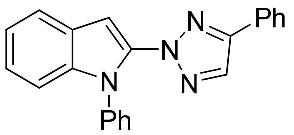

1-phenyl-2-(4-phenyl-2H-1,2,3-triazol-2-yl)-1H-indole 3d:

Obtained as a white solid in 56.8% yield; M.p. 91 – 93 °C; ^{1}H NMR (400 MHz,

$CDCl_3$) δ 8.02 (s, 1H), 7.92 – 7.78 (m, 3H), 7.43 (t, J = 20.5 Hz, 11H), 7.15 (s, 1H); ^{13}C NMR (101 MHz, $CDCl_3$) δ 149.06, 136.91, 136.60, 135.28, 133.03, 129.70, 129.39, 129.08, 128.07, 127.56, 126.27, 123.87, 121.67, 121.63, 111.11, 98.86; IR (neat) 3061, 3028, 1607, 1534, 1461, 1356, 1055, 946, 768, 723, 692, 483cm^{-1}; HRMS(ESI) m/z calcd for $C_{22}H_{16}N_4$, $[M+H]^+$ 337.1448, found 337.1444

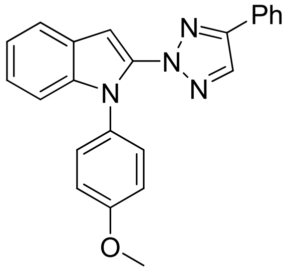

1-(4-methoxyphenyl)-2-(4-phenyl-2H-1,2,3-triazol-2-yl)-1H-indole 3e ：

Obtained as a white solid in 51.2% yield; M.p. 94 – 96 °C; ^1H NMR (400 MHz, $CDCl_3$) δ 7.94 (s, 1H), 7.76 – 7.71 (m, 3H), 7.45 – 7.39 (m, 2H), 7.37 (d, J = 7.2 Hz, 1H), 7.27 – 7.22 (m, 5H), 6.97 (s, 1H), 6.91 (d, J = 8.9 Hz, 2H), 3.81 (s, 3H); ^{13}C NMR (101 MHz, $CDCl_3$) δ 159.12, 148.87, 136.81, 135.28, 132.84, 129.51, 129.22, 128.95, 128.79, 126.15, 125.94, 123.56, 121.45, 121.25, 114.38, 110.97, 98.30, 55.56; IR (neat): 3118, 3035, 2924, 1589, 1496, 1454, 1328, 1091, 975, 858, 767, 688, 505cm^{-1}; HRMS (ESI) Calcd for $C_{23}H_{18}N_4O$ [M+H]+: 367.1553; Found: 367.1541

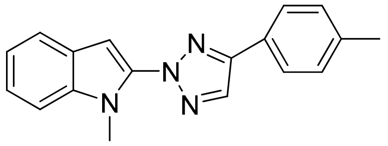

1-methyl-2-(4-p-tolyl-2H-1,2,3-triazol-2-yl)-1H-indole 3f :

Obtained as a white solid in 58.6% yield; M.p. 94 – 96 °C; ^1H NMR (400 MHz, $CDCl_3$) δ 8.09 (s, 1H), 7.78 (d, J = 8.1 Hz, 2H), 7.66 (d, J = 7.9 Hz, 1H), 7.38 (d, J = 8.2 Hz, 1H), 7.34 – 7.25 (m, 3H), 7.19 (t, J = 7.0 Hz, 1H), 6.83 (s, 1H), 3.87 (s, 3H), 2.40 (s, 3H); ^{13}C NMR (101 MHz, $CDCl_3$) δ 149.21, 139.17, 135.89, 135.48, 132.85, 129.76, 126.76, 126.15, 122.88, 121.36, 120.65, 109.79, 96.18, 30.87, 21.44; IR (neat) 3056, 3032, 2931, 1559, 1467, 1331, 1145, 966, 772, 701, 536, 458cm^{-1}; HRMS(ESI) m/z calcd for $C_{18}H_{16}N_4$, $[M+H]^+$ 289.1448, found 289.1439

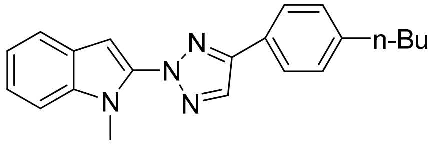

2-(4-(4-butylphenyl)-2H-1,2,3-triazol-2-yl)-1-methyl-1H-indole 3g :

Obtained as a white solid in 51.9% yield; M.p. 87 – 89 °C; ^1H NMR (400 MHz, $CDCl_3$) δ 8.07 (s, 1H), 7.78 (d, J = 8.1 Hz, 2H), 7.65 (d, J = 7.9 Hz, 1H), 7.35 (d, J = 8.1 Hz, 1H), 7.27 (t, J = 7.8 Hz, 3H), 7.17 (t, J = 7.4 Hz, 1H), 6.83 (s, 1H), 3.85 (s, 3H), 2.69 – 2.60 (m, 2H), 1.69 – 1.56 (m, 2H), 1.43 – 1.30 (m, 2H), 0.93 (t, J = 7.3 Hz, 3H); ^{13}C NMR (101 MHz, $CDCl_3$) δ 149.48, 144.22, 135.94, 135.54, 132.88, 129.15, 127.13, 126.20, 122.91, 121.38, 120.68, 109.81, 96.18, 35.58, 33.59, 30.89, 22.42, 14.06; IR (neat) 3059, 3028, 1594, 1523, 1336, 988, 956, 845, 747, 526, 412cm^{-1};

HRMS(ESI) m/z calcd for $C_{21}H_{22}N_4$, $[M+H]^+$ 331.1917, found 331.1909

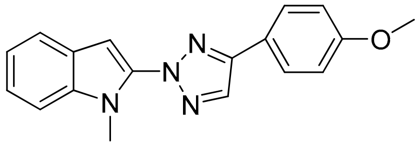

2-(4-(4-methoxyphenyl)-2H-1,2,3-triazol-2-yl)-1-methyl-1H-indole 3h :
Obtained as a white solid in 59.6% yield; M.p. 92 – 93 °C; ^{1}H NMR (400 MHz, $CDCl_3$) δ 8.05 (s, 1H), 7.82 (d, J = 8.8 Hz, 2H), 7.66 (d, J = 7.9 Hz, 1H), 7.38 (d, J = 8.2 Hz, 1H), 7.31 (t, J = 7.6 Hz, 1H), 7.18 (t, J = 7.4 Hz, 1H), 6.99 (d, J = 8.8 Hz, 2H), 6.83 (s, 1H), 3.87 (s, 3H), 3.84 (s, 3H); ^{13}C NMR (101 MHz, $CDCl_3$) δ 160.26, 149.09, 135.86, 135.49, 132.51, 127.57, 126.12, 122.82, 122.18, 121.30, 120.60, 114.44, 109.50, 95.96, 55.37, 30.82; IR (neat) 3061, 2986, 1628, 1495, 1441, 1276, 1063, 987, 823, 715, 681, 512cm^{-1}; HRMS(ESI) m/z calcd for $C_{18}H_{16}N_4O$, $[M+H]^+$ 305.1397, found 305.1387

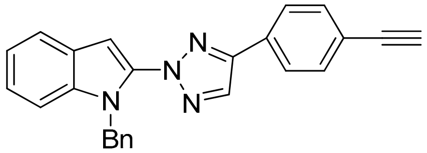

1-benzyl-2-(4-(4-ethynylphenyl)-2H-1,2,3-triazol-2-yl)-1H-indole 3i :
Obtained as a white solid in 52.5% yield; M.p. 108 – 110 °C; ^{1}H NMR (400 MHz, $CDCl_3$) δ 8.06 (s, 1H), 7.75 (d, J = 8.0 Hz, 2H), 7.69 (d, J = 7.7 Hz, 1H), 7.55 (d, J = 8.0 Hz, 2H), 7.32 (d, J = 8.1 Hz, 1H), 7.26 – 7.15 (m, 5H), 7.05 (d, J = 7.2 Hz, 2H), 6.93 (s, 1H), 5.60 (s, 2H), 3.15 (s, 1H); ^{13}C NMR (101 MHz, $CDCl_3$) δ 148.36, 137.30, 135.74, 135.14, 133.11, 132.76, 129.83, 128.62, 127.38, 126.45, 126.30, 126.02, 123.31, 122.74, 121.51, 121.01, 110.51, 97.00, 83.26, 78.53, 47.67; IR (neat) 3356, 3069, 3027, 2109, 1612, 1574, 1452, 1326, 956, 811, 707, 652, 485cm^{-1}; HRMS(ESI) m/z calcd for $C_{25}H_{18}N_4$, $[M+H]^+$ 375.1604, found 375.1593

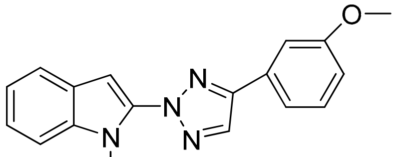

2-(4-(3-methoxyphenyl)-2H-1,2,3-triazol-2-yl)-1-methyl-1H-indole 3j :
Obtained as a white solid in 57.7% yield; M.p. 102 – 104 °C; ^{1}H NMR (400 MHz, $CDCl_3$) δ 8.05 (s, 1H), 7.82 (d, J = 8.3 Hz, 2H), 7.66 (d, J = 7.8 Hz, 1H), 7.37 (d, J = 8.1 Hz, 1H), 7.30 (t, J = 7.5 Hz, 1H), 7.18 (t, J = 7.3 Hz, 1H), 6.99 (d, J = 8.3 Hz, 2H), 6.83 (s, 1H), 3.87 (s, 3H), 3.84 (s, 3H); ^{13}C NMR (101 MHz, $CDCl_3$) δ 160.33, 149.09, 135.85, 135.48, 132.51, 127.56, 126.11, 122.82, 122.18, 121.30, 120.60, 114.20, 109.73, 96.08, 55.37, 30.70; IR (neat) 3059, 2923, 1619, 1486, 1442, 1253, 1072, 986, 836, 727, 692, 518cm^{-1}; HRMS(ESI) m/z calcd for $C_{18}H_{16}N_4O$, $[M+H]^+$ 305.1397, found 305.1390

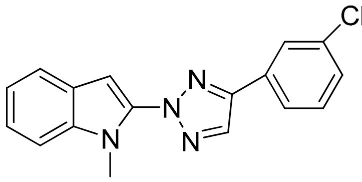

2-(4-(3-chlorophenyl)-2H-1,2,3-triazol-2-yl)-1-methyl-1H-indole 3k :

Obtained as a white solid in 64.9% yield; M.p. 88 – 90 °C; ^{1}H NMR (400 MHz, $CDCl_3$) δ 8.04 (s, 1H), 7.86 (s, 1H), 7.69 (td, J = 4.0, 1.5 Hz, 1H), 7.64 (d, J = 7.9 Hz, 1H), 7.36 – 7.26 (m, 4H), 7.17 (t, J = 6.7 Hz, 1H), 6.82 (s, 1H), 3.82 (s, 3H); ^{13}C NMR (101 MHz, $CDCl_3$) δ 147.79, 135.83, 135.11, 134.93, 132.89, 131.24, 130.21, 128.96, 126.14, 125.93, 124.17, 122.94, 121.30, 120.66, 109.73, 96.19, 30.81; IR (neat) 3062, 3016, 1703, 1561, 1467, 1352, 1266, 1056, 956, 796, 737, 628, 491cm^{-1}; HRMS(ESI) m/z calcd for $C_{17}H_{13}ClN_4$, $[M+H]^+$ 309.0902, found 309.0893

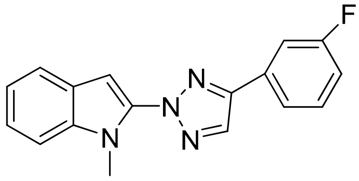

2-(4-(3-fluorophenyl)-2H-1,2,3-triazol-2-yl)-1-methyl-1H-indole 3l :

Obtained as a white solid in 62.1% yield; M.p. 80 – 82 °C; ^{1}H NMR (400 MHz, $CDCl_3$) δ 8.11 (s, 1H), 7.66 (t, J = 7.7 Hz, 2H), 7.61 (dd, J = 9.6, 2.0 Hz, 1H), 7.46 – 7.36 (m, 2H), 7.32 (t, J = 7.6 Hz, 1H), 7.19 (t, J = 7.4 Hz, 1H), 7.09 (td, J = 8.4, 2.5 Hz, 1H), 6.85 (s, 1H), 3.88 (s, 3H); ^{13}C NMR (101 MHz, $CDCl_3$) δ 164.45, 162.00, 148.14, 135.92, 135.21, 133.03, 131.70 (d, J = 8.4 Hz), 130.67 (d, J = 8.3 Hz), 126.03, 123.03, 121.85 (d, J = 2.4 Hz), 121.40, 120.73, 116.96 (d, J = 21.3 Hz), 113.16 (d, J = 23.0 Hz), 109.79, 96.35, 30.87; IR (neat) 3059, 3036, 1601, 1553, 1492, 1352, 1231, 1142, 956, 843, 737, 628, 503, 492cm^{-1}; HRMS(ESI) m/z calcd for $C_{17}H_{13}FN_4$, $[M+H]^+$ 293.1197, found 293.1191

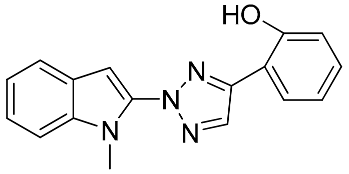

2-(2-(1-methyl-1H-indol-2-yl)-2H-1,2,3-triazol-4-yl)phenol 3m :

Obtained as a white solid in 46.6% yield; M.p. 132 – 135 °C; ^{1}H NMR (400 MHz, $CDCl_3$) δ 9.46 (s, 1H), 8.25 (s, 1H), 7.69 (d, J = 7.6 Hz, 2H), 7.41 (d, J = 8.2 Hz, 1H), 7.38 – 7.30 (m, 2H), 7.22 (t, J = 7.4 Hz, 1H), 7.11 (d, J = 8.2 Hz, 1H), 7.02 (t, J = 7.5 Hz, 1H), 6.85 (s, 1H), 3.86 (s, 3H).^{1}H NMR (400 MHz, $CDCl_3$): δ 9.47 (s, 1H), 8.26 (s, 1H), 7.69 (d, J = 8.0Hz, 2H), 7.42 (d, J = 8.2Hz, 1H), 7.38-7.33 (m, 2H), 7.23 (t, J = 7.4Hz, 1H), 7.11 (d, J = 8.3Hz, 1H), 7.03 (t, J = 7.4Hz, 1H) 6.85 (s, 1H), 3.87 (s, 3H); ^{13}C NMR (101 MHz, $CDCl_3$) δ 155.42, 154.18, 135.86, 132.78, 130.95, 126.66, 123.34, 121.43, 120.92, 120.08, 117.63, 109.86, 96.80, 88.55, 86.71, 30.82; IR (neat) 3108, 3049, 2986, 1663, 1564, 1474, 1366, 996, 757, 727, 691, 479cm^{-1}; HRMS(ESI) m/z calcd for $C_{17}H_{14}N_4O$, $[M+H]^+$ 291.1240, found 291.1236

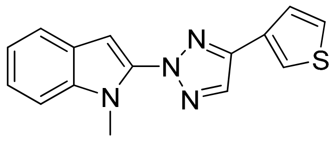

1-methyl-2-(4-(thiophen-3-yl)-2H-1,2,3-triazol-2-yl)-1H-indole 3n :

Obtained as a white solid in 50.6% yield; M.p. 92 – 94 ºC; ^{1}H NMR (400 MHz, $CDCl_3$) δ 7.97 (s, 1H), 7.70 (d, J = 2.0 Hz, 1H), 7.64 (d, J = 7.9 Hz, 1H), 7.51 (d, J = 4.8 Hz, 1H), 7.39 – 7.36 (m, 1H), 7.34 (d, J = 8.2 Hz, 1H), 7.28 (t, J = 7.5 Hz, 1H), 7.17 (t, J = 7.3 Hz, 1H), 6.82 (s, 1H), 3.82 (s, 3H); ^{13}C NMR (101 MHz, $CDCl_3$) δ 145.31, 135.82, 135.26, 133.09, 130.85, 126.73, 126.03, 125.91, 122.87, 122.61, 121.30, 120.61, 109.72, 96.24, 30.44; IR (neat) 3033, 2932, 1586, 1454, 1336, 1189, 965, 873, 769, 715, 473cm^{-1}; HRMS(ESI) m/z calcd for $C_{15}H_{12}N_4S$, $[M+H]^+$ 281.0855, found 281.0847

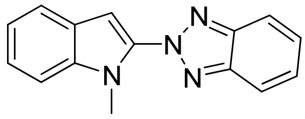

2-(1-methyl-1H-indol-2-yl)-2H-benzo[d][1,2,3]triazole 3o :

Obtained as a white solid in 53.5% yield; M.p. 137 – 139 ºC; ^{1}H NMR (400 MHz, $CDCl_3$) δ 7.95 (dd, J = 6.6, 3.1 Hz, 2H), 7.70 (d, J = 7.9 Hz, 1H), 7.50 – 7.38 (m, 3H), 7.34 (t, J = 7.5 Hz, 1H), 7.21 (t, J = 7.1 Hz, 1H), 7.06 (s, 1H), 4.00 (s, 3H); ^{13}C NMR (101 MHz, $CDCl_3$) δ 144.93, 136.51, 135.80, 127.48, 126.06, 123.41, 121.59, 120.89, 118.31, 109.79, 97.65, 31.44; IR (neat) 3052, 3028, 1568, 1452, 1346, 1271, 1059, 956, 771, 732, 692, 433cm^{-1}; HRMS(ESI) m/z calcd for $C_{15}H_{12}N_4$, $[M+H]^+$ 249.1135, found 249.1124

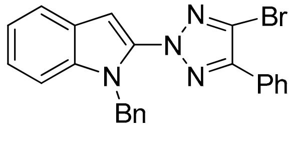

1-benzyl-2-(4-bromo-5-phenyl-2H-1,2,3-triazol-2-yl)-1H-indole 3p

Obtained as a white solid in 53% yield; M.p. 90 – 91 ºC; ^{1}H NMR (400 MHz, $CDCl_3$) δ 7.92 (d, J = 6.5 Hz, 2H), 7.70 (d, J = 7.8 Hz, 1H), 7.50 – 7.40 (m, 3H), 7.34 (d, J = 8.1 Hz, 1H), 7.28 (d, J = 7.0 Hz, 1H), 7.24 – 7.17 (m, 4H), 7.05 (d, J = 6.6 Hz, 2H), 6.93 (s, 1H), 5.61 (s, 2H); ^{13}C NMR (101 MHz, $CDCl_3$) δ 146.99, 137.07, 135.75, 134.54, 129.39, 128.71, 128.64, 128.32, 127.50, 127.45, 126.77, 126.51, 126.11, 123.52, 121.62, 121.08, 110.51, 97.33, 47.65; IR (neat) 3059, 3030, 2924, 1558, 1454, 1328, 1161, 1010, 956, 727, 694cm^{-1}; HRMS (ESI) Calcd for $C_{23}H_{18}BrN_4$ $[M+H]^+$: 429.07094; Found: 429.06996.

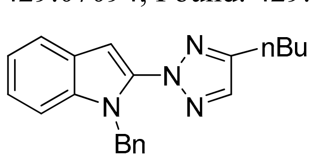

1-Benzyl-2-(4-butyl-2H-1,2,3-triazol-2-yl)-1H-indole 3q

Obtained as a colorless oil in 60% yield; ^{1}H NMR (400 MHz, $CDCl_3$) δ 7.59 (d, J = 7.7 Hz, 1H), 7.51 (s, 1H), 7.22 (d, J = 8.0 Hz, 1H), 7.18 – 7.05 (m, 5H), 6.94 (d, J =

6.9 Hz, 2H), 6.74 (s, 1H), 5.46 (s, 2H), 2.66 (t, J = 7.7 Hz, 2H), 1.83 – 1.46 (m, 2H), 1.36 – 1.20 (m, 2H), 0.85 (t, J = 7.3 Hz, 3H); ^{13}C NMR (101 MHz, $CDCl_3$) δ 150.56, 137.32, 135.46, 134.87, 128.54, 127.28, 126.49, 126.38, 122.96, 121.37, 120.77, 110.44, 96.66, 47.42, 31.09, 25.14, 22.15, 13.75; IR (neat) 3059, 3030, 2929, 1566, 1456, 1346, 1161, 1029, 954, 748, 727, 694cm^{-1}; HRMS (ESI) Calcd for $C_{21}H_{23}N_4$ [M+H]$^+$: 331.1923; Found: 331.1910.

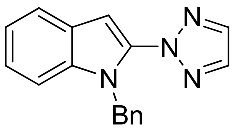

1-Benzyl-2-(2H-1,2,3-triazol-2-yl)-1H-indole 3r

Obtained as a white solid in 95% yield; M.p. 95 – 98 °C; ^{1}H NMR (400 MHz, $CDCl_3$) δ 7.76 (s, 2H), 7.61 (d, J = 7.7 Hz, 1H), 7.22 (d, J = 8.2 Hz, 1H), 7.17 – 7.07 (m, 5H), 6.94 (d, J = 6.7 Hz, 2H), 6.80 (s, 1H), 5.45 (s, 2H); ^{13}C NMR (101 MHz, $CDCl_3$) δ 137.16, 135.97, 135.51, 135.14, 128.69, 127.37, 126.43, 126.25, 123.24, 121.58, 120.92, 110.43, 97.20, 47.57; IR (neat) 3059, 3032, 2360, 1566, 1454, 1163, 945, 748, 727, 455cm^{-1}; HRMS (ESI) Calcd for C17H15N4 [M+H]$^+$: 275.1297; Found: 275.1285.

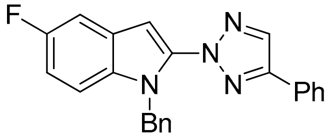

5-Fluoro-2-(4-phenyl-2H-1,2,3-triazol-2-yl)-1H-indole 3s

Obtained as a white solid in 78% yield; M.p. 116 – 119 °C; ^{1}H NMR (400 MHz, $CDCl_3$) δ 8.08 (s, 1H), 7.81 (d, J = 7.0 Hz, 2H), 7.48 – 7.38 (m, 3H), 7.33 (dd, J = 9.2, 2.4 Hz, 1H), 7.26 – 7.18 (m, 4H), 7.06 (d, J = 6.6 Hz, 2H), 6.98 (td, J = 9.1, 2.5 Hz, 1H), 6.89 (s, 1H), 5.61 (s, 2H); ^{13}C NMR (101 MHz, $CDCl_3$) δ 158.46(d, *J* =235.9Hz), 149.40, 137.06, 136.43, 133.17, 132.19, 129.38, 129.19, 129.04, 128.69, 127.50, 126.70, 126.59, 126.45, 126.22, 111.84, 111.53(d, *J* =9.5Hz), 111.39, 106.24(d, *J* =23.6Hz), 96.72(d, *J* =4.5Hz), 96.69, 47.85; IR (neat) 3064, 3034, 1722, 1585, 1454, 1342, 1122, 950, 767, 690, 520cm^{-1}; HRMS (ESI) Calcd for $C_{23}H_{18}FN_4$ [M+H]$^+$: 369.1515; Found: 369.1502.

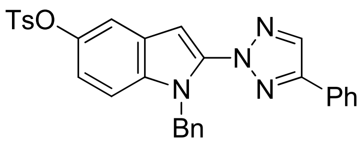

1-Benzyl-2-(4-phenyl-2H-1,2,3-triazol-2-yl)-1H-indol-5-yl-4-methylbenzenesulfonate 3t

Obtained as a white solid in 83% yield; M.p. 169 – 171 °C; ^{1}H NMR (400 MHz, $CDCl_3$) δ 8.07 (s, 1H), 7.80 (d, J = 6.9 Hz, 2H), 7.70 (d, J = 8.3 Hz, 2H), 7.48 – 7.36 (m, 3H), 7.28 (d, J = 8.1 Hz, 2H), 7.25-7.18 (m, 5H), 7.04 (d, J = 6.3 Hz, 2H), 6.89 (dd, J = 8.9, 2.3 Hz, 1H), 6.84 (s, 1H), 5.58 (s, 2H), 2.43 (s, 3H); ^{13}C NMR (101 MHz, $CDCl_3$) δ 149.50, 145.15, 144.25, 136.78, 136.42, 133.98, 133.28, 132.48, 129.71, 129.26, 129.06, 128.70, 128.64, 127.58, 126.50, 126.22, 123.62, 118.06, 114.65,

111.24, 97.07, 47.96, 21.76; IR (neat) 3064, 3034, 1732, 1581, 1477, 1454, 1176, 1091, 948, 854, 692, 551cm^{-1}; HRMS (ESI) Calcd for C30H25N4O3S $[M+H]^+$: 521.1647; Found: 521.1646.

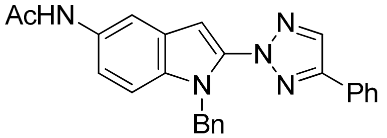

N-(1-Benzyl-2-(4-phenyl-2H-1,2,3,-triazol-2-yl)-1H-indol-5-yl)acetamide 3u
Obtained as a white solid in 60% yield; M.p. 128 – 131 °C; ^{1}H NMR (400 MHz, $CDCl_3$) δ 8.06 (s, 1H), 7.87 (br, 1H), 7.80 (d, J = 7.2 Hz, 2H), 7.51 – 7.36 (m, 4H), 7.30 – 7.15 (m, 5H), 7.04 (d, J = 6.8 Hz, 2H), 6.87 (s, 1H), 5.58 (s, 2H), 2.17 (s, 3H); ^{13}C NMR (101 MHz, $CDCl_3$) δ 168.44, 149.27, 137.20, 135.88, 133.04, 131.50, 129.43, 129.13, 129.02, 128.63, 127.40, 126.46, 126.21, 117.51, 113.23, 110.78, 96.88, 47.77, 24.49; IR (neat) 3062, 3032, 1658, 1550, 1485, 1452, 1276, 956, 767, 692cm^{-1}; HRMS (ESI) Calcd for C25H22N5O $[M+H]^+$: 408.1824; Found: 408.1818.

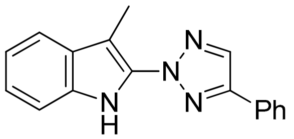

3-Methyl-2-(4-phenyl-2H-1,2,3,-triazol-2-yl)-1H-indole 3v
Obtained as a white solid in 55% yield; M.p. 132 – 134 °C; ^{1}H NMR (400 MHz, $CDCl_3$) δ 8.88 (br, 1H), 8.03 (s, 1H), 7.86 (d, J = 5.3 Hz, 2H), 7.60 (d, J = 7.8 Hz, 1H), 7.45 (t, J = 7.4 Hz, 2H), 7.41 – 7.35 (m, 1H), 7.31 (d, J = 8.0 Hz, 1H), 7.23 (t, J = 7.0 Hz, 1H), 7.17 (t, J = 7.0 Hz, 1H), 2.66 (s, 3H); ^{13}C NMR (101 MHz, $CDCl_3$) δ 148.41, 132.86, 132.15, 130.67, 129.67, 129.14, 129.05, 129.02, 126.17, 123.07, 120.22, 119.38, 110.88, 100.67, 9.02; IR (neat) 3118, 3035, 2924, 1589, 1496, 1454, 1328, 1091, 975, 858, 767, 688, 505cm^{-1}; HRMS (ESI) Calcd for C17H15N4 $[M+H]^+$: 275.1297; Found: 275.1286.

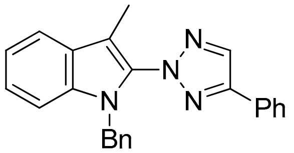

1-Benzyl-3-methyl-2-(4-phenyl-2H-1,2,3,-triazol-2-yl)-1H-indole 3w
Obtained as a white solid in 75% yield; M.p. 78 – 80 °C; ^{1}H NMR (400 MHz, $CDCl_3$) δ 8.09 (s, 1H), 7.81 (d, J = 7.0 Hz, 2H), 7.66 (d, J = 7.8 Hz, 1H), 7.43 (t, J = 7.3 Hz, 2H), 7.40 – 7.34 (m, 1H), 7.30 – 7.23 (m, 2H), 7.21 – 7.13 (m, 4H), 7.03 (d, J = 6.4 Hz, 2H), 5.26 (s, 2H), 2.36 (s, 3H); ^{13}C NMR (101 MHz, $CDCl_3$) δ 149.24, 137.35, 134.70, 133.01, 131.66, 129.69, 129.09, 129.07, 128.60, 127.39, 126.90, 126.68, 126.26, 123.67, 120.17, 119.99, 110.33, 107.43, 47.18, 8.39; IR (neat) 3061, 3034, 1622, 1454, 1350, 1186, 1091, 956, 740, 692, 455cm^{-1}; HRMS (ESI) Calcd for C24H21N4 $[M+H]^+$: 365.1766; Found: 365.1756.

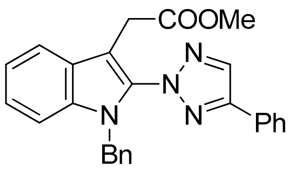

Methyl 2-(1-benzyl-2-(4-phenyl-2H-1,2,3,-triazol-2-yl)-1H-indol-3-yl)acetate 3x

Obtained as a white solid in 67% yield; M.p. 104 – 106 °C; ^{1}H NMR (400 MHz, $CDCl_3$) δ 8.11 (s, 1H), 7.82 (d, J = 7.0 Hz, 2H), 7.69 (d, J = 7.8 Hz, 1H), 7.45 (t, J = 7.3 Hz, 2H), 7.42 – 7.37 (m, 1H), 7.33 – 7.27 (m, 2H), 7.24 – 7.18 (m, 4H), 7.06 (d, J = 6.6 Hz, 2H), 5.42 (s, 2H), 3.91 (s, 2H), 3.63 (s, 3H); ^{13}C NMR (101 MHz, $CDCl_3$) δ 171.49, 149.37, 137.06, 134.65, 133.19, 132.61, 129.49, 129.14, 129.03, 128.61, 127.41, 126.62, 126.23, 123.76, 120.82, 119.90, 110.55, 103.39, 52.09, 47.46, 29.79; IR (neat) 3061, 3034, 1737, 1456, 1348, 1192, 1166, 975, 744, 692, cm^{-1}; HRMS (ESI) Calcd for $C_{26}H_{23}N_4O_2$ $[M+H]^+$: 423.1821; Found: 423.1825.

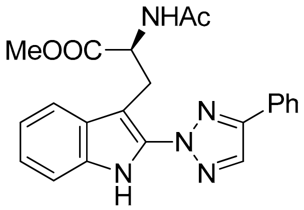

(S)-Methyl 2-acetamido-3-(2-(4-phenyl-2H-1,2,3,-triazol-2-yl)-1H-indol-3-yl) propanoate 3y

Obtained as a yellow solid in 62% yield, $[\alpha]_D^{20}$ = + 15.1 (*c* = 1.00); M.p. 137 – 140 °C; ^{1}H NMR (400 MHz, DMSO) δ 12.10 (br, 1H), 8.74 (s, 1H) , 8.47 (d, *J* =7.6Hz, 1H), 8.06 (d, J = 7.2 Hz, 2H), 7.64 (d, J = 7.9 Hz, 1H), 7.56 (t, J = 7.5 Hz, 2H), 7.50 – 7.42 (m, 2H), 7.20 (t, J = 7.5 Hz, 1H), 7.11 (t, J = 7.4 Hz, 1H), 4.69 (q, J = 7.5 Hz, 1H), 3.64 (dd, J = 13.9, 7.4 Hz, 1H), 3.44 (s, 3H), 3.37 (dd, J = 13.9, 7.5 Hz, 1H), 1.77 (s, 3H); ^{13}C NMR (101 MHz, DMSO) δ 172.82, 169.65, 148.85, 133.76, 133.60, 131.78, 129.63, 129.58, 128.12, 126.49, 125.90, 122.93, 120.23, 119.27, 112.15, 100.02, 53.34, 52.10, 26.63, 22.76; IR (neat) 3062, 2254, 1739, 1666, 1456, 1373, 1286, 1026, 977, 769, 696, 507cm^{-1}; HRMS (ESI) Calcd for $C_{22}H_{22}N_5O_3$ $[M+H]^+$: 404.1723; Found: 404.1714.

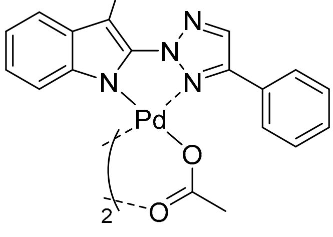

Compound 5

Obtained as a yellow solid in 45% yield, ^{1}H NMR (400 MHz, $CDCl_3$) δ 7.43 – 7.38 (m, 1H), 7.36 (dt, J = 7.8, 3.8 Hz, 1H), 7.01 – 6.94 (m, 1H), 6.72 (d, J = 8.0 Hz, 1H), 6.61 (ddd, J = 7.9, 5.2, 2.7 Hz, 1H), 6.57 (s, 1H), 2.38 (s, 1H), 2.30 (s, 1H); ^{13}C NMR (101 MHz, $CDCl_3$) δ 193.64, 186.49, 180.98, 173.69, 161.51, 129.30, 128.48, 126.61, 126.02, 121.68, 119.74, 117.96, 111.80, 98.63, 23.79, 7.65.

References:

1, (a) Parker, C. A.; Rees, W. T. Analyst, **1960**, 85, 587. (b) Forgues, S. F.; Lavabre, D. *J. Chem. Educ.* **1999**, 76,1260. (c) Berlman, I. B. Handbook of fluorescence spectra of aromatic molecules, Academic Press, New York, **1971**. (d) Ajayaghosh, A.; Carol, P.; Sreejith, S. *J. Am. Chem. Soc.* **2005**, 127, 14962. (e) Lin, W.; Yuan, L.; Feng, J.; Cao, X. *Eur. J. Org. Chem.* **2008**, 2689.

2, Jin, T.; Kamijo, S.; Yamamoto, Y. Eur. *J. Org. Chem.* **2004**, 3789.

3, Choy, P. Y.; Lau, C. P.; Kwong, F. Y. *J. Org. Chem.* **1999**, 64, 611.

4, Huang, Q.; Zheng, M.; Yang, S.; Kuang, C.; Yu, C.; Yang, Q. *Eur. J. Med. Chem.* **2011**, 46, 5680.

5, Qinquan H.; Yi L.; Xiaocong D.; Yanjun L.; and Yunfeng C.; *Advanced Synthesis & Catalysis*, **2016** , 358, 1689.

6, Novak, L.; Hornyhszky, G.; Rohalya, J.; Kolonits, P.; Szhtay, C. Liebigs Ann. **1995**, 1877.

7, Peng, Y. L.; Zhao, Z. G.; Liu, X. L.; Li, G. H. Res Chem Intermed, **2013**, 39, 1897.

8, Wuming Y.; Qiaoyi W.; Quan L.; Minyong L.; Jeffrey L. Petersen, and Xiaodong S.; *Chem. Eur. J.* **2011**, 17, 5011.

H[1] NMR and C[13] NMR spectra

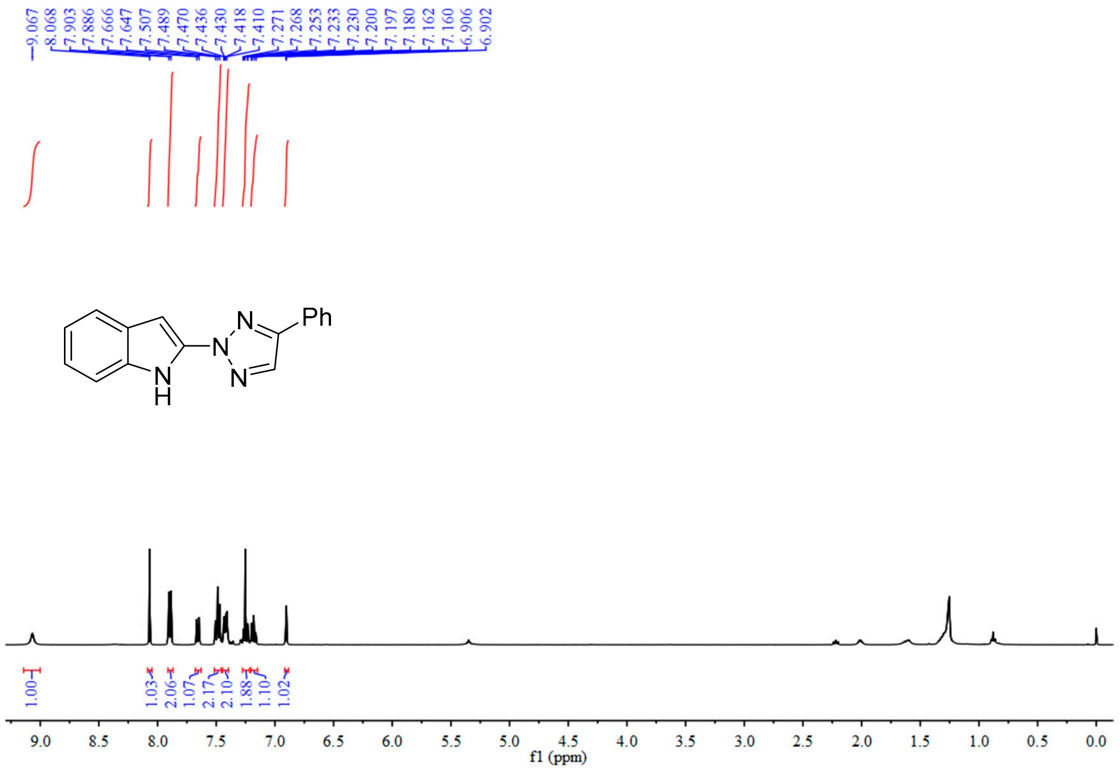

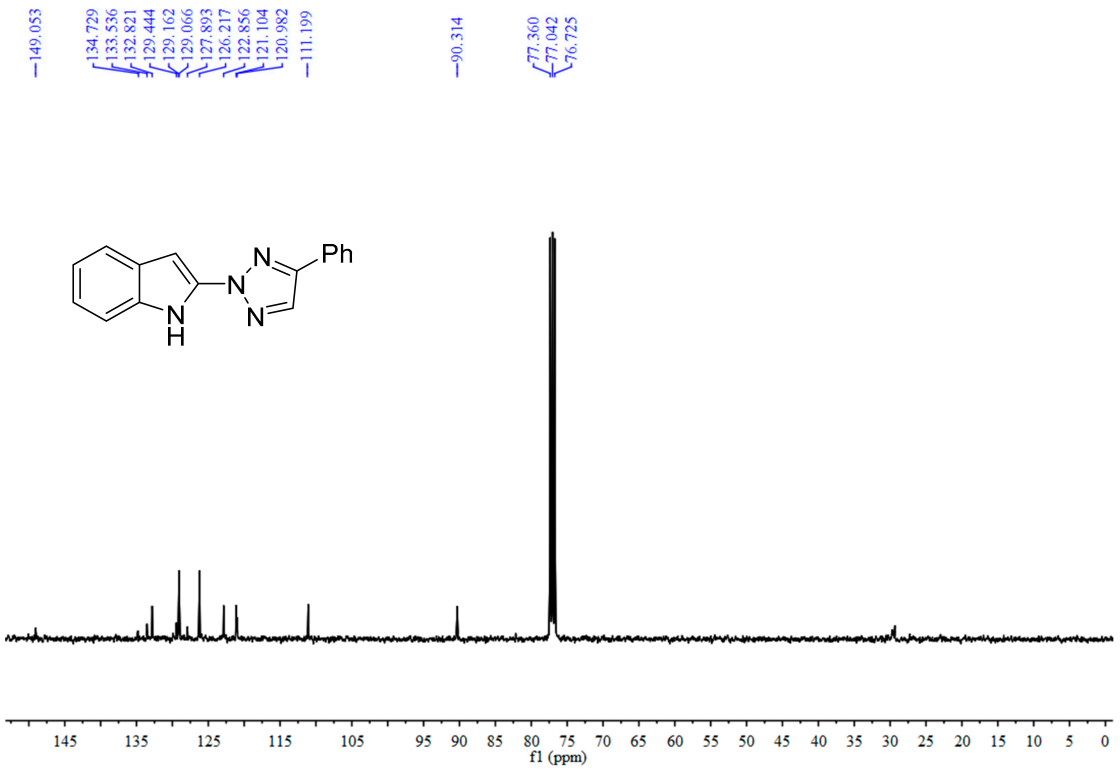

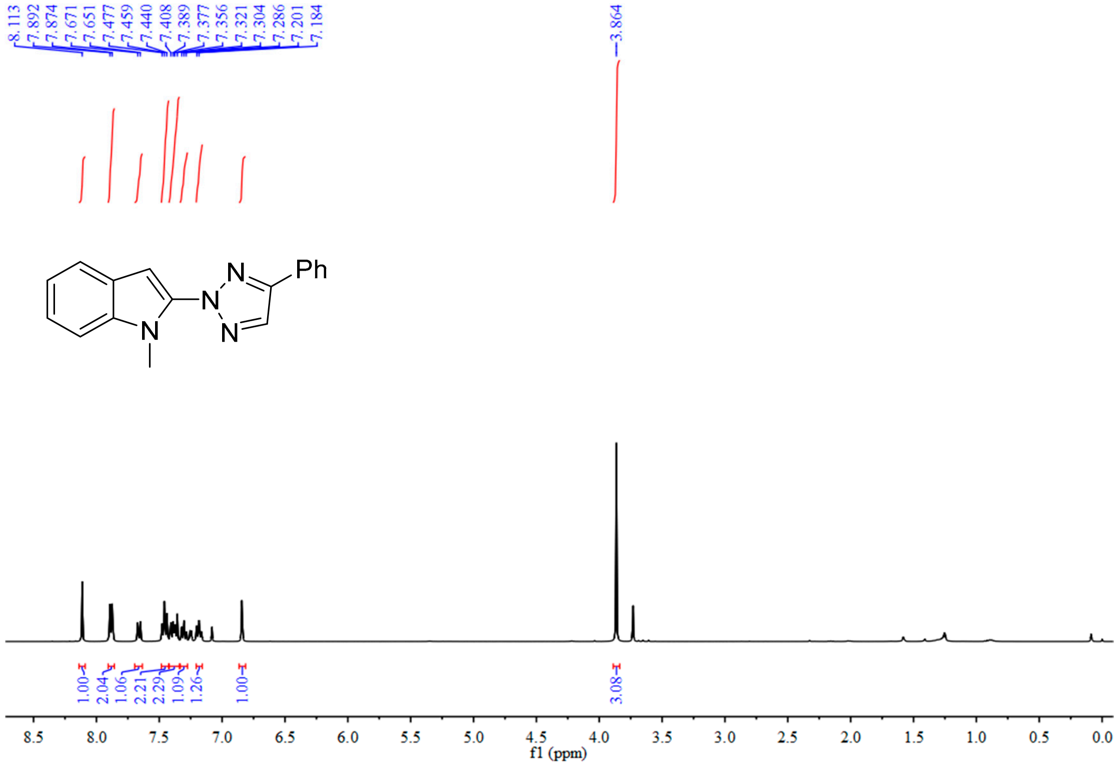
8.113
7.892
7.874
7.671
7.651
7.477
7.459
7.440
7.408
7.389
7.377
7.356
7.321
7.304
7.286
7.201
7.184
3.864
Ph
N
N
N
N
1.00
2.04
1.06
2.21
2.29
1.09
1.26
1.00
3.08
8.5
8.0
7.5
7.0
6.5
6.0
5.5
5.0
4.5
4.0
3.5
3.0
2.5
2.0
1.5
1.0
0.5
0.0
f1 (ppm)

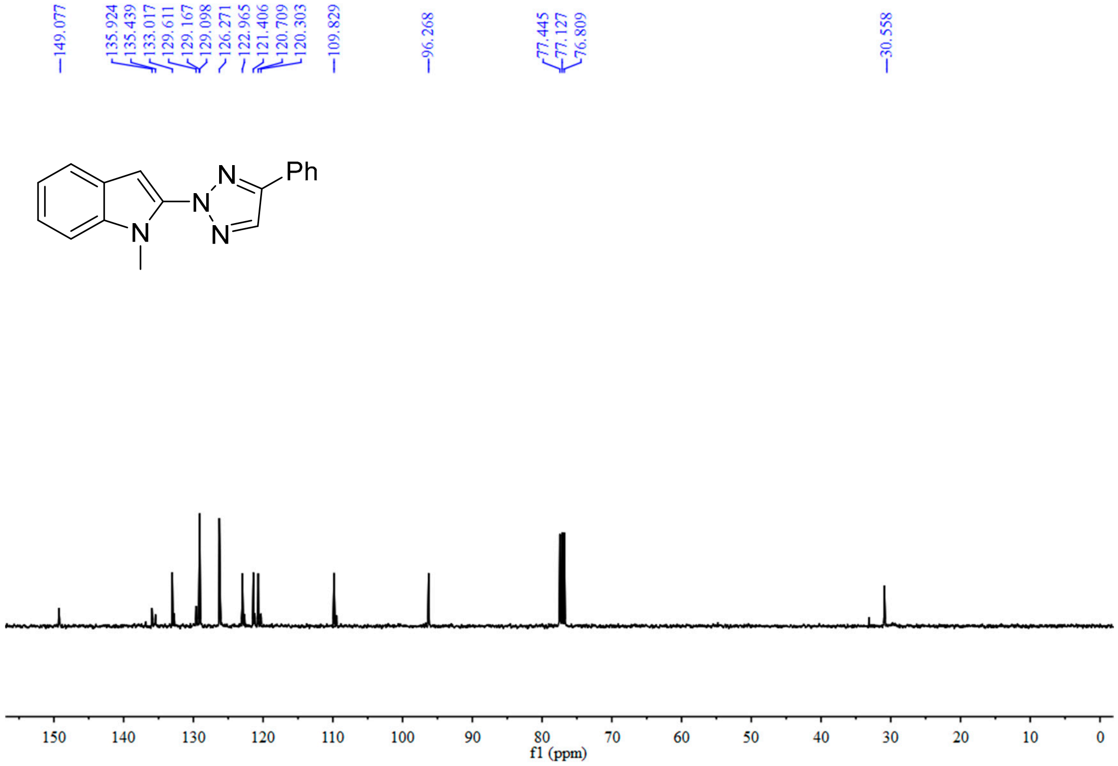
149.077
135.924
135.439
133.017
129.611
129.167
129.098
126.271
122.965
121.406
120.709
120.303
109.829
96.268
77.445
77.127
76.809
30.558
Ph
N
N
N
N
150
140
130
120
110
100
90
80
70
60
50
40
30
20
10
0
f1 (ppm)

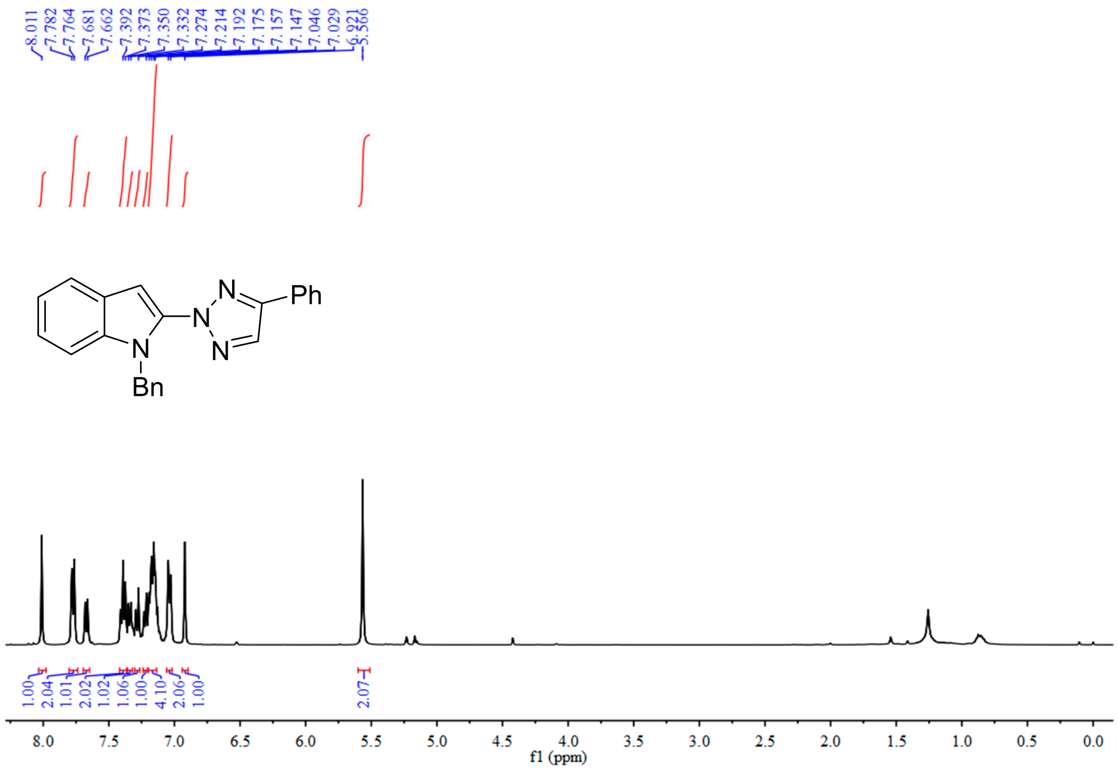
8.011
7.782
7.764
7.681
7.662
7.392
7.373
7.350
7.332
7.274
7.214
7.192
7.175
7.157
7.147
7.046
7.029
6.921
5.566
N
N
N
Ph
N
Bn
1.00
2.04
1.01
2.02
1.02
1.06
1.00
4.10
2.06
1.00
2.07
f1 (ppm)

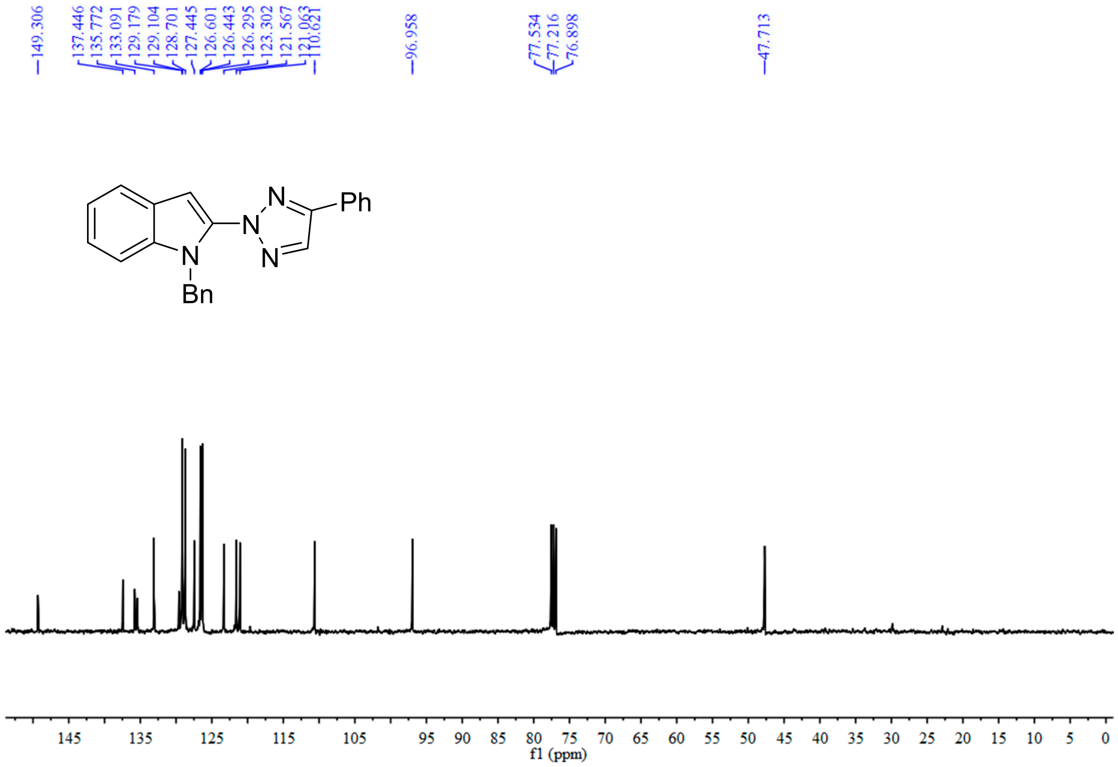
149.306
137.446
135.772
133.091
129.179
129.104
128.701
127.445
126.601
126.443
126.295
123.302
121.567
121.063
110.621
96.958
77.534
77.216
76.898
47.713
N
N
N
Ph
N
Bn
f1 (ppm)

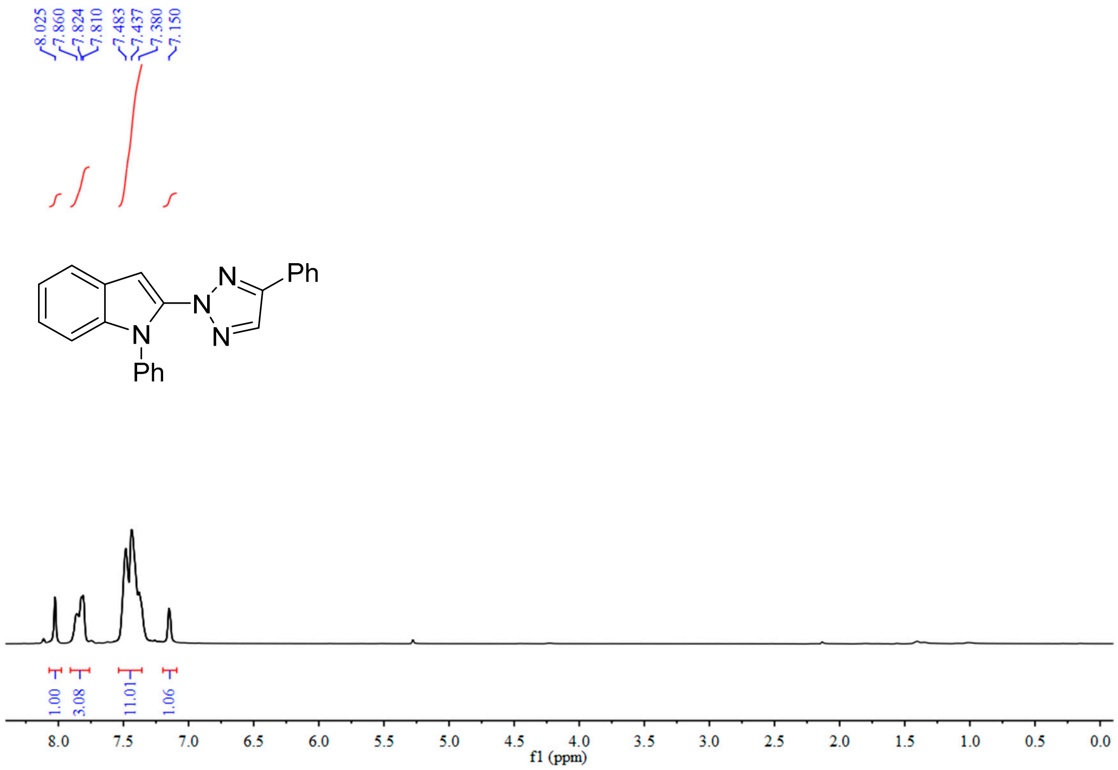
8.025
7.860
7.824
7.810
7.483
7.437
7.380
7.150
N
N
N
N
Ph
Ph
1.00
3.08
11.01
1.06
8.0 7.5 7.0 6.5 6.0 5.5 5.0 4.5 4.0 3.5 3.0 2.5 2.0 1.5 1.0 0.5 0.0
f1 (ppm)

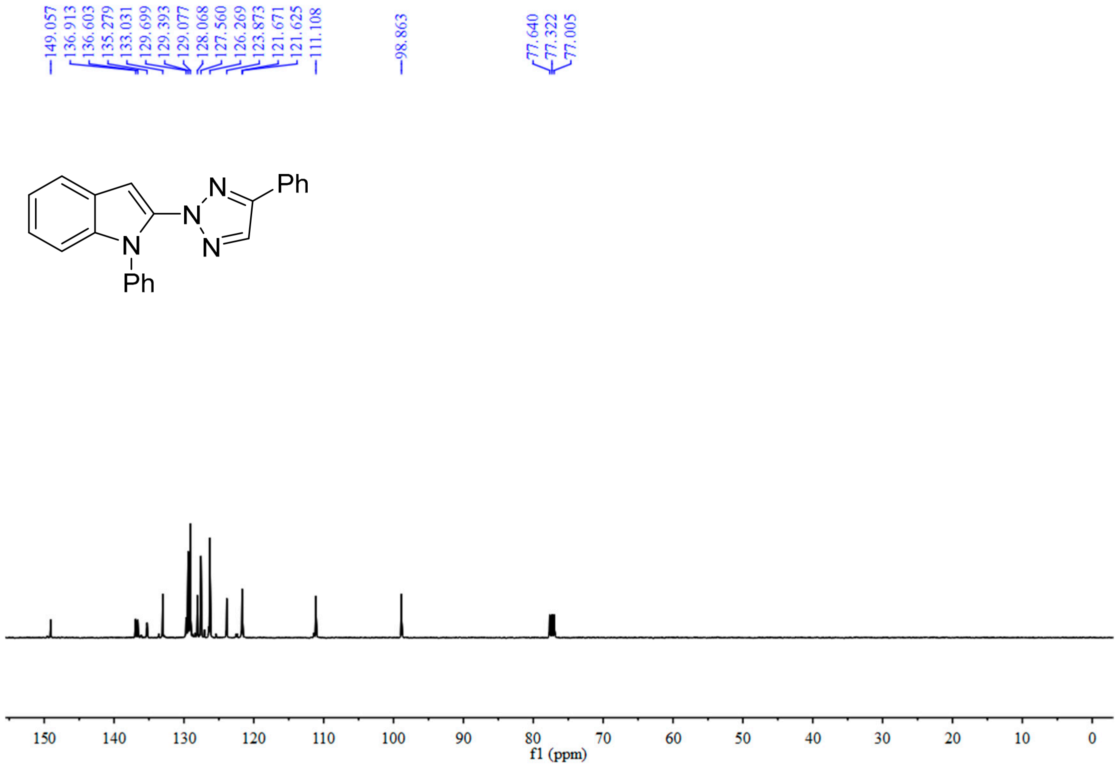
149.057
136.913
136.603
135.279
133.031
129.699
129.393
129.077
128.068
127.560
126.269
123.873
121.671
121.625
111.108
98.863
77.640
77.322
77.005
N
N
N
N
Ph
Ph
150 140 130 120 110 100 90 80 70 60 50 40 30 20 10 0
f1 (ppm)

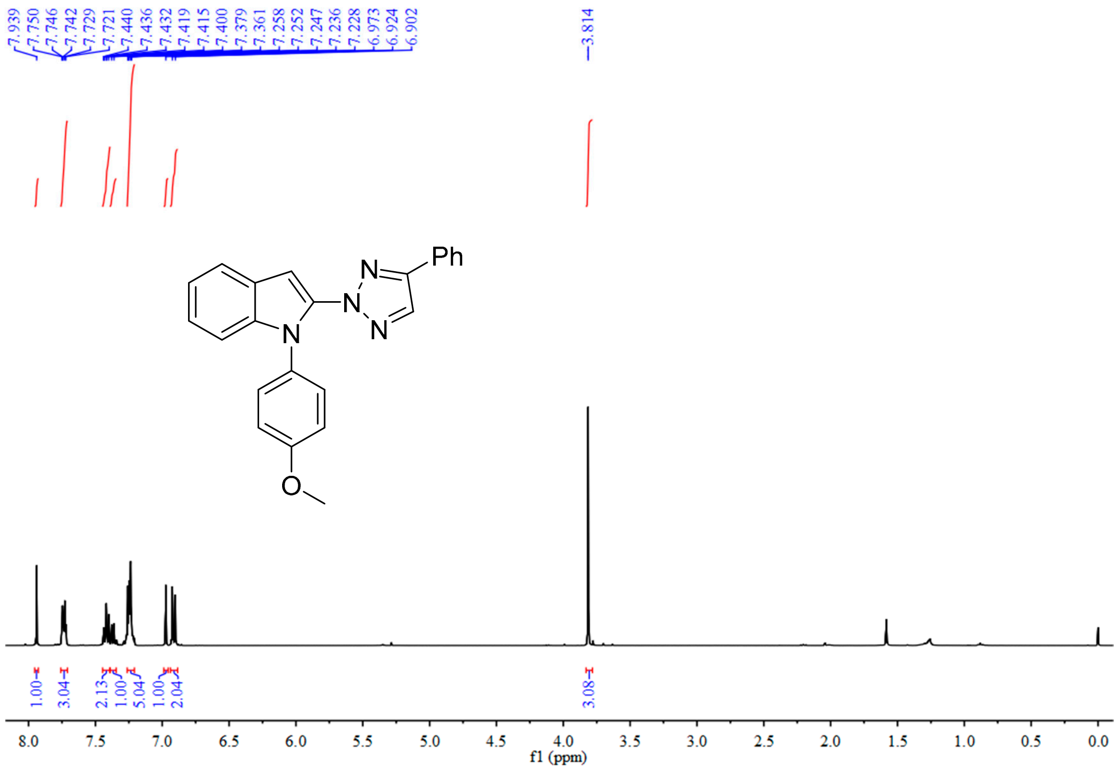

7.939
7.750
7.746
7.742
7.729
7.721
7.440
7.436
7.432
7.419
7.415
7.400
7.379
7.361
7.258
7.252
7.247
7.236
7.228
6.973
6.924
6.902
3.814
Ph
N
N
N
N
O
1.00
3.04
2.13
1.00
5.04
1.00
2.04
3.08
8.0
7.5
7.0
6.5
6.0
5.5
5.0
4.5
4.0
3.5
3.0
2.5
2.0
1.5
1.0
0.5
0.0
f1 (ppm)

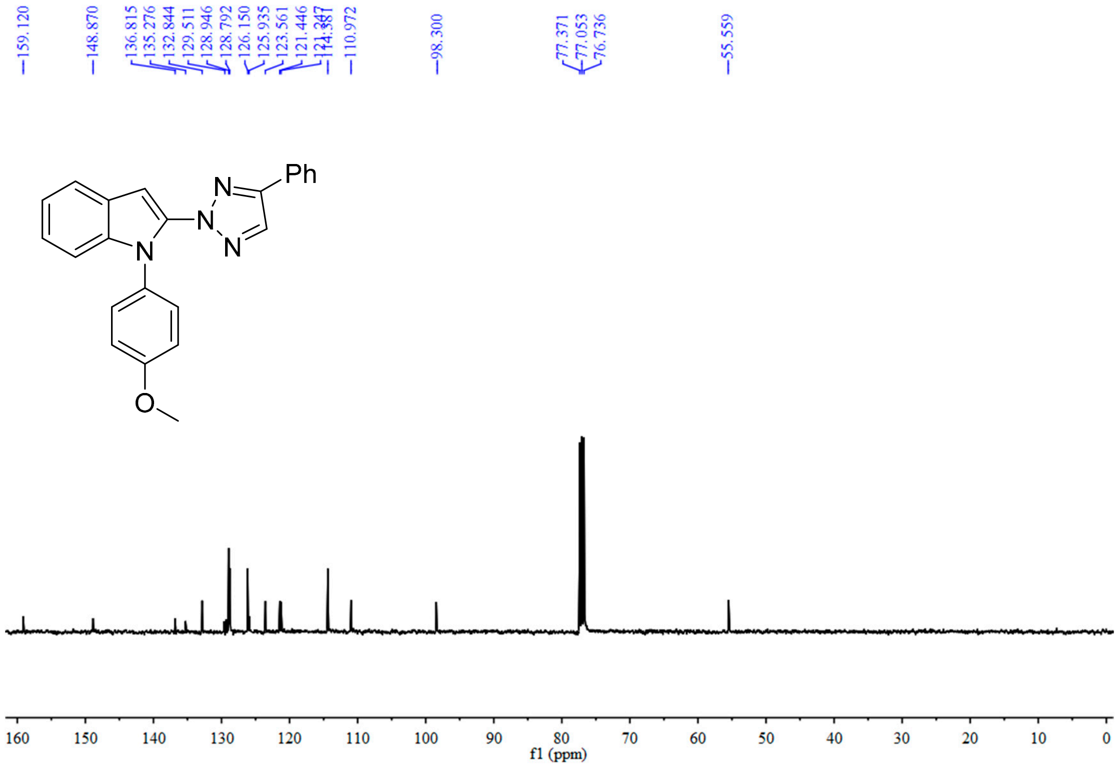

159.120
148.870
136.815
135.276
132.844
129.511
128.946
128.792
126.150
125.935
123.561
121.446
114.581
110.972
98.300
77.371
77.053
76.736
55.559
Ph
N
N
N
N
O
160
150
140
130
120
110
100
90
80
70
60
50
40
30
20
10
0
f1 (ppm)

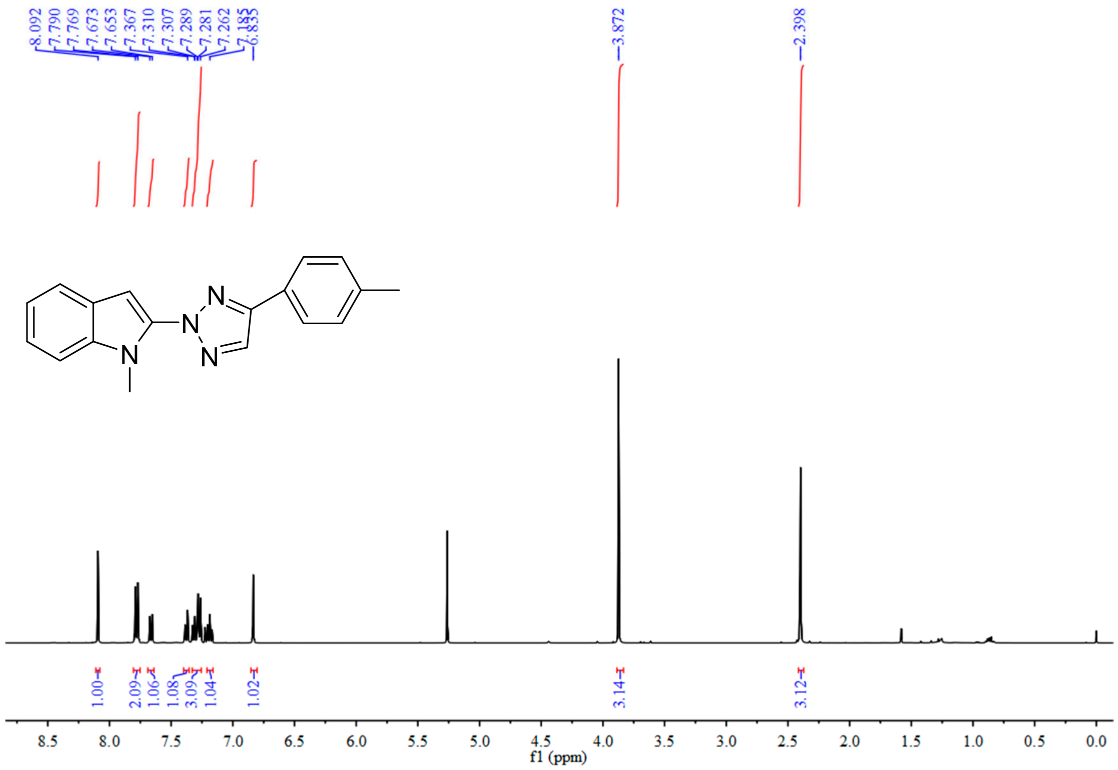
8.092
7.790
7.769
7.673
7.653
7.367
7.310
7.307
7.289
7.281
7.262
7.185
6.835
3.872
2.398
N
N
N
N
1.00
2.09
1.06
1.08
3.09
1.04
1.02
3.14
3.12
8.5
8.0
7.5
7.0
6.5
6.0
5.5
5.0
4.5
4.0
3.5
3.0
2.5
2.0
1.5
1.0
0.5
0.0
f1 (ppm)

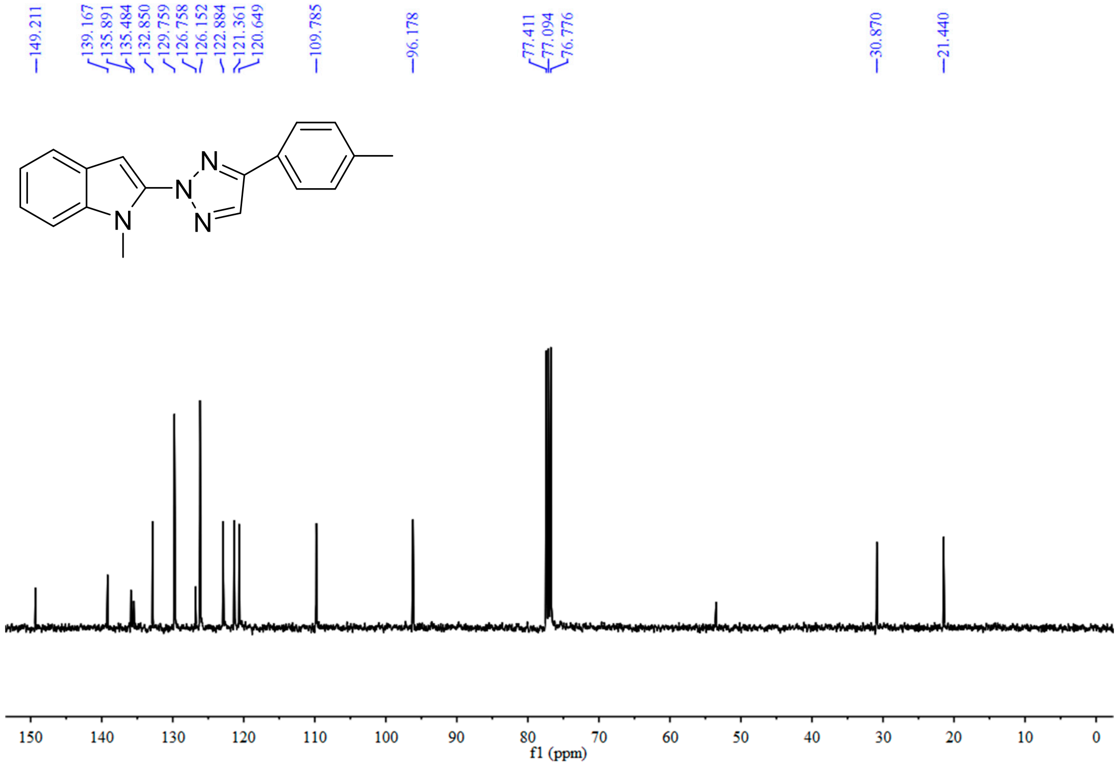
149.211
139.167
135.891
135.484
132.850
129.759
126.758
126.152
122.884
121.361
120.649
109.785
96.178
77.411
77.094
76.776
30.870
21.440
N
N
N
N
150
140
130
120
110
100
90
80
70
60
50
40
30
20
10
0
f1 (ppm)

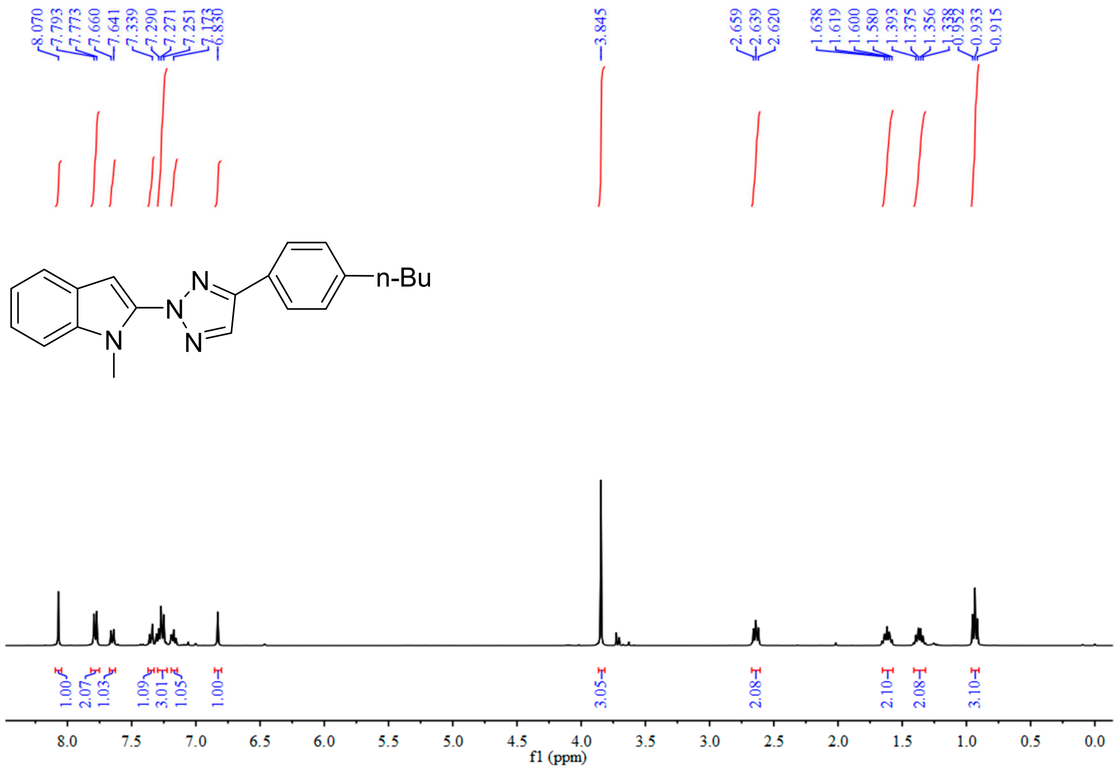
8.070
7.793
7.773
7.660
7.641
7.339
7.290
7.271
7.251
6.830
3.845
2.659
2.639
2.620
1.638
1.619
1.600
1.580
1.393
1.375
1.356
0.933
0.915
n-Bu
N
1.00
2.07
1.03
1.09
3.01
1.05
1.00
3.05
2.08
2.10
2.08
3.10
8.0 7.5 7.0 6.5 6.0 5.5 5.0 4.5 4.0 3.5 3.0 2.5 2.0 1.5 1.0 0.5 0.0
f1 (ppm)

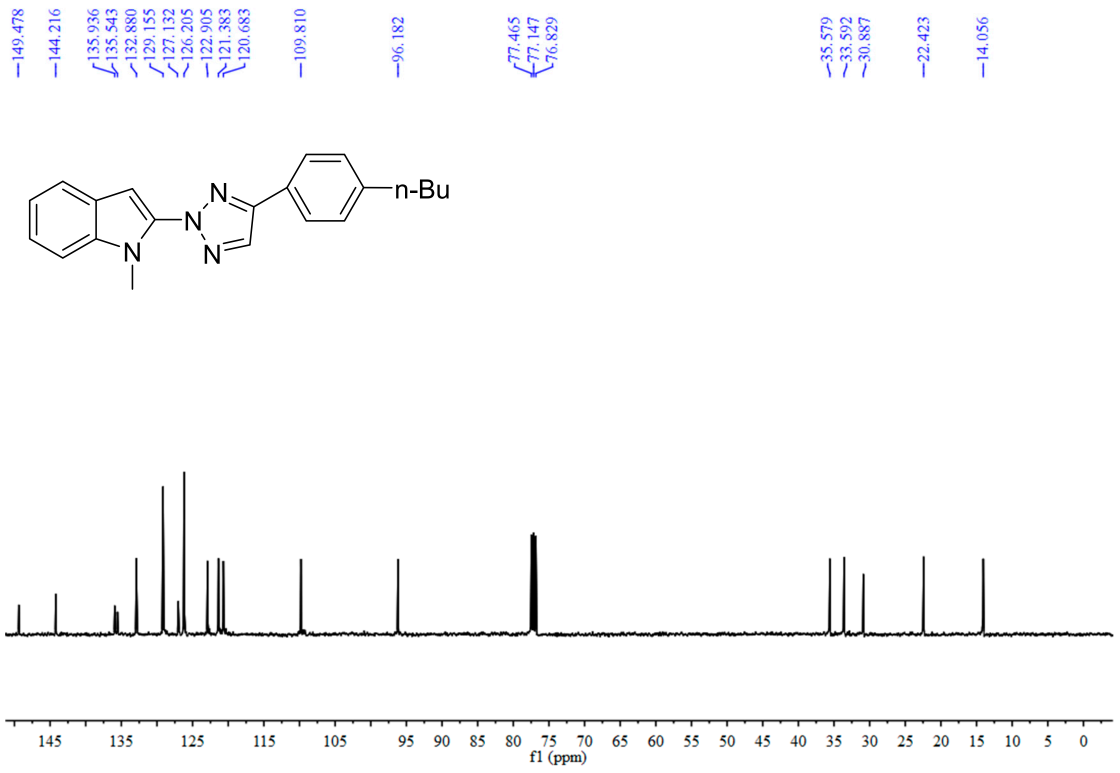
149.478
144.216
135.936
135.543
132.880
129.155
127.132
126.205
122.905
121.383
120.683
109.810
96.182
77.465
77.147
76.829
35.579
33.592
30.887
22.423
14.056
n-Bu
N
145 135 125 115 105 95 90 85 80 75 70 65 60 55 50 45 40 35 30 25 20 15 10 5 0
f1 (ppm)

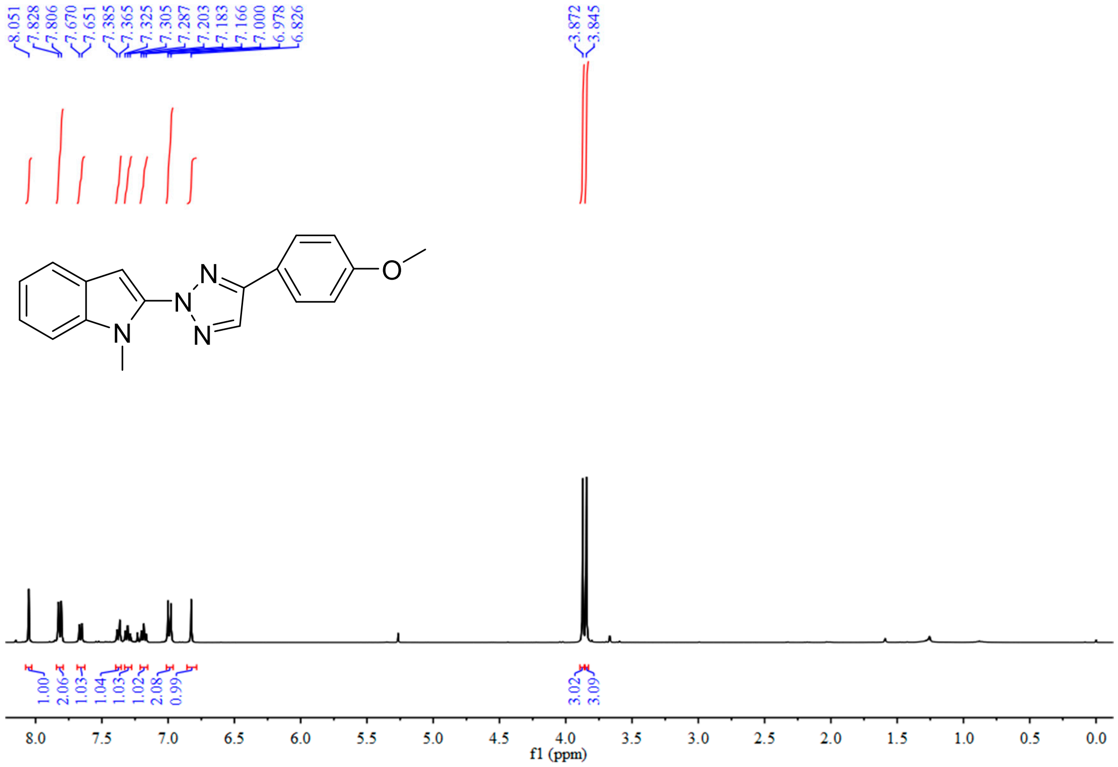
8.051
7.828
7.806
7.670
7.651
7.385
7.365
7.325
7.305
7.287
7.203
7.183
7.166
7.000
6.978
6.826
3.872
3.845
N
N
N
N
O
1.00
2.06
1.03
1.04
1.03
1.02
2.08
0.99
3.02
3.09
8.0 7.5 7.0 6.5 6.0 5.5 5.0 4.5 4.0 3.5 3.0 2.5 2.0 1.5 1.0 0.5 0.0
f1 (ppm)

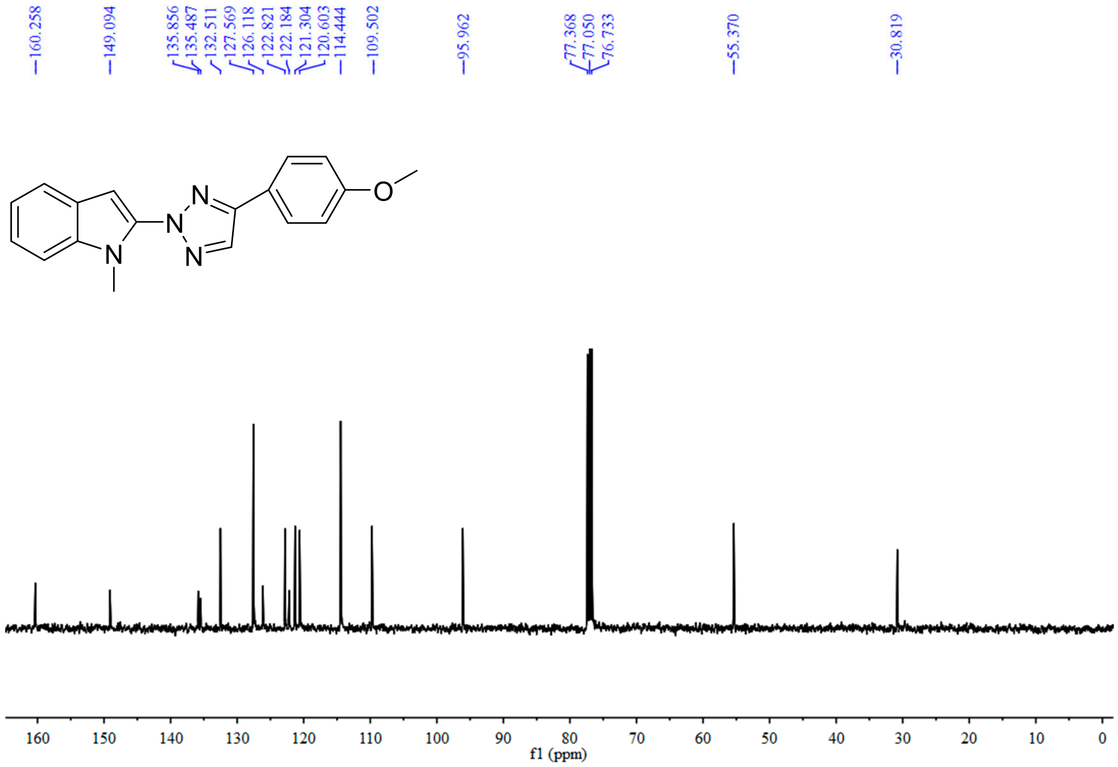
160.258
149.094
135.856
135.487
132.511
127.569
126.118
122.821
122.184
121.304
120.603
114.444
109.502
95.962
77.368
77.050
76.733
55.370
30.819
N
N
N
N
O
160 150 140 130 120 110 100 90 80 70 60 50 40 30 20 10 0
f1 (ppm)

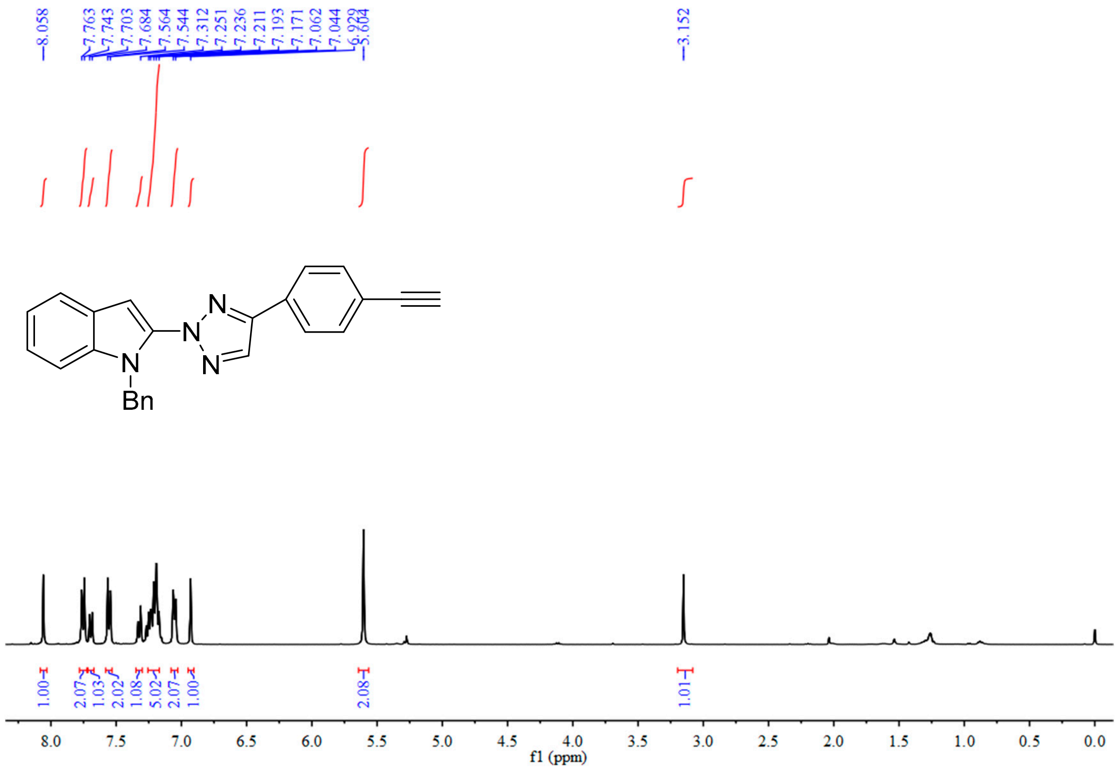
8.058
7.763
7.743
7.703
7.684
7.564
7.544
7.312
7.251
7.236
7.211
7.193
7.171
7.062
7.044
6.929
5.604
3.152
N
N
N
N
Bn
1.00
2.07
1.03
2.02
1.08
5.02
2.07
1.00
2.08
1.01
f1 (ppm)

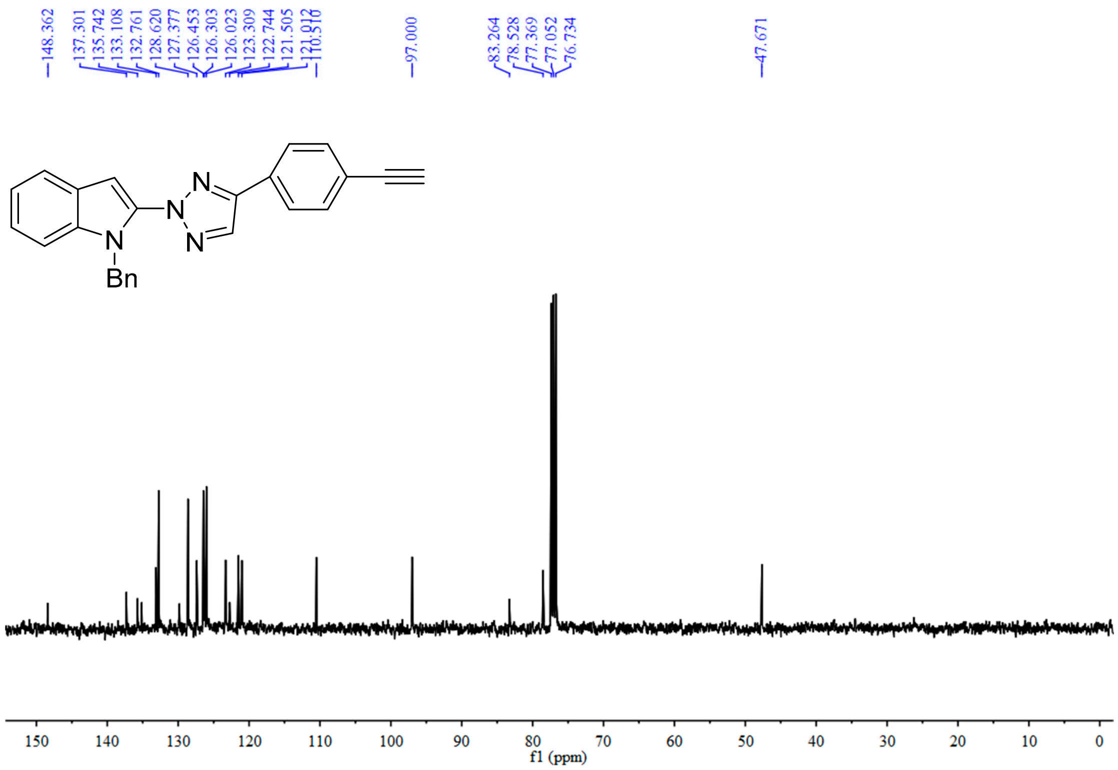
148.362
137.301
135.742
133.108
132.761
128.620
127.377
126.453
126.303
126.023
123.309
122.744
121.505
121.012
110.510
97.000
83.264
78.528
77.369
77.052
76.734
47.671
N
N
N
N
Bn
f1 (ppm)

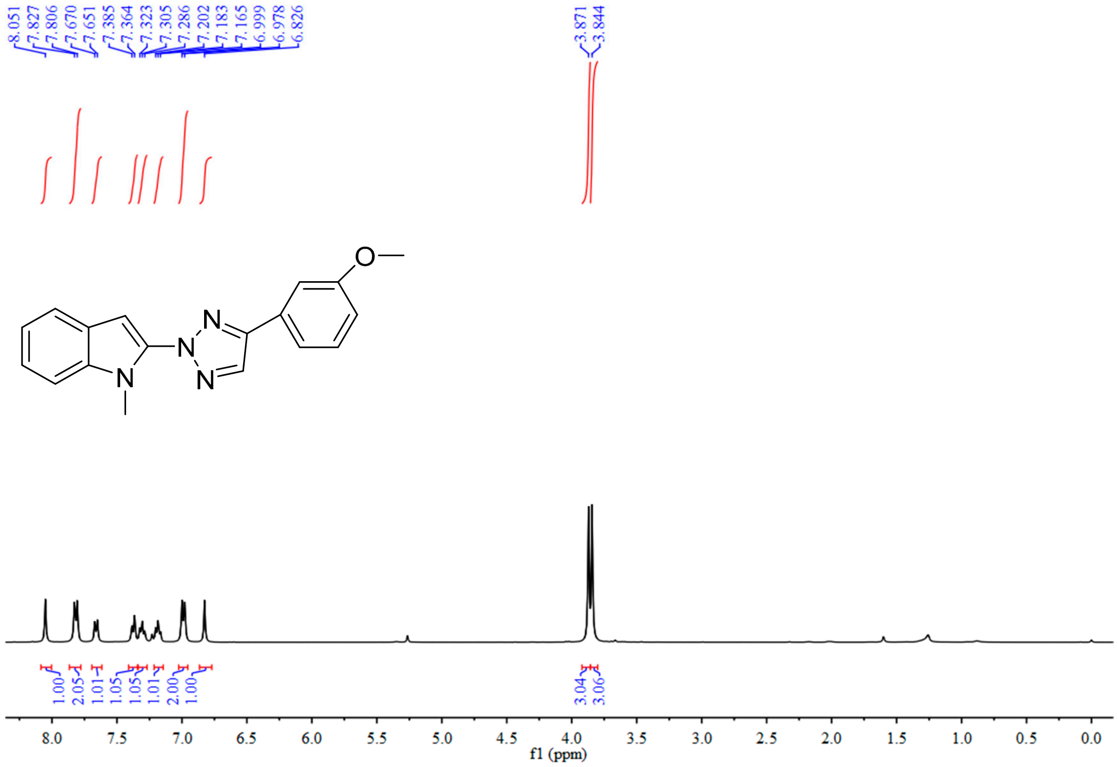
8.051
7.827
7.806
7.670
7.651
7.385
7.364
7.323
7.305
7.286
7.202
7.183
7.165
6.999
6.978
6.826
3.871
3.844
N
N
N
N
O
1.00
2.05
1.01
1.05
1.05
1.01
2.00
1.00
3.04
3.06
f1 (ppm)

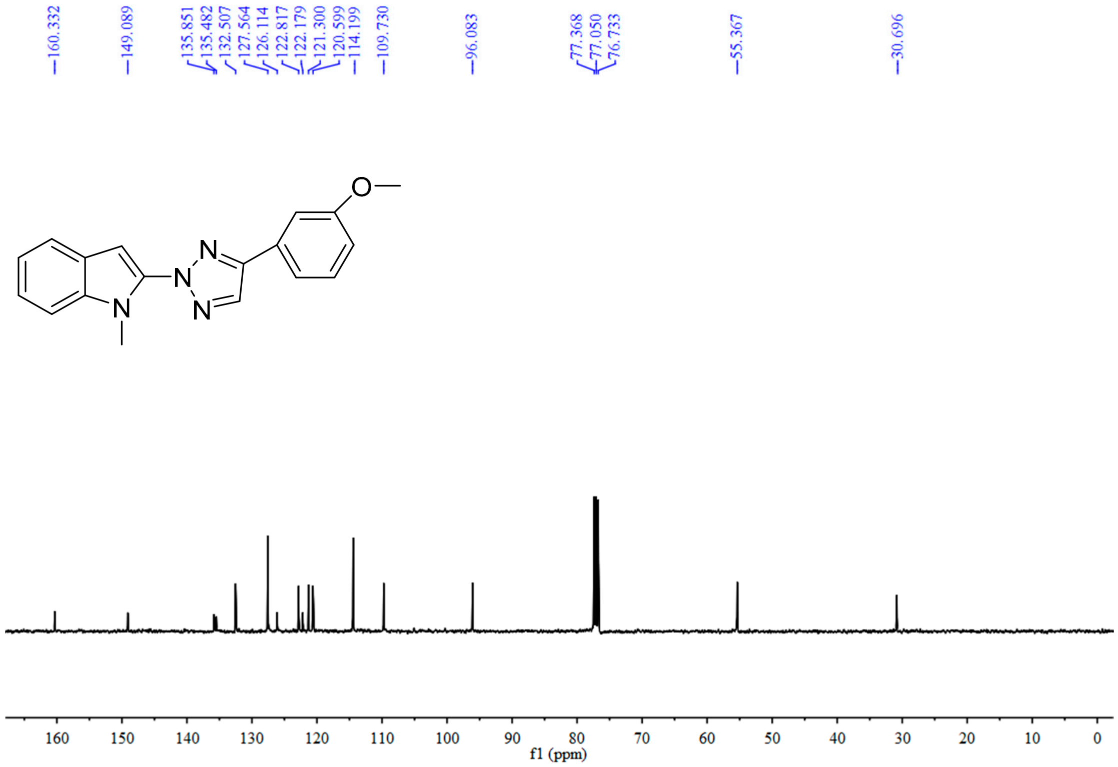
160.332
149.089
135.851
135.482
132.507
127.564
126.114
122.817
122.179
121.300
120.599
114.199
109.730
96.083
77.368
77.050
76.733
55.367
30.696
N
N
N
N
O
f1 (ppm)

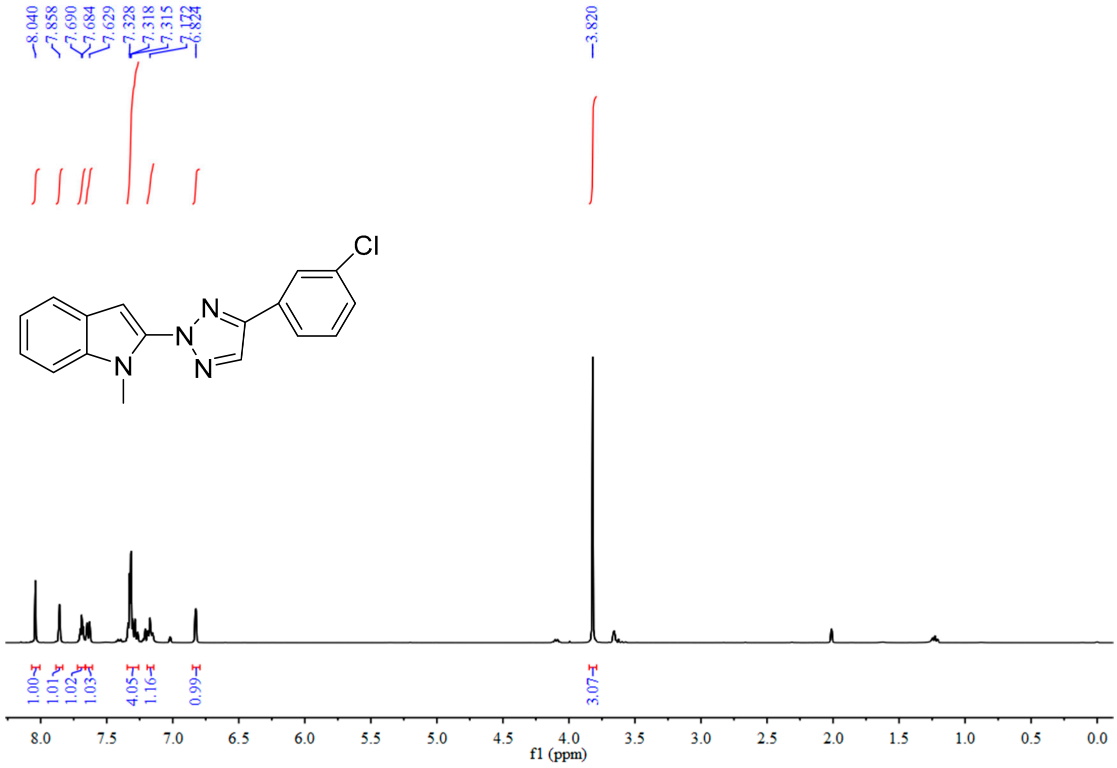
8.040
7.858
7.690
7.684
7.629
7.328
7.318
7.315
7.172
6.824
3.820
1.00
1.01
1.02
1.03
4.05
1.16
0.99
3.07
f1 (ppm)

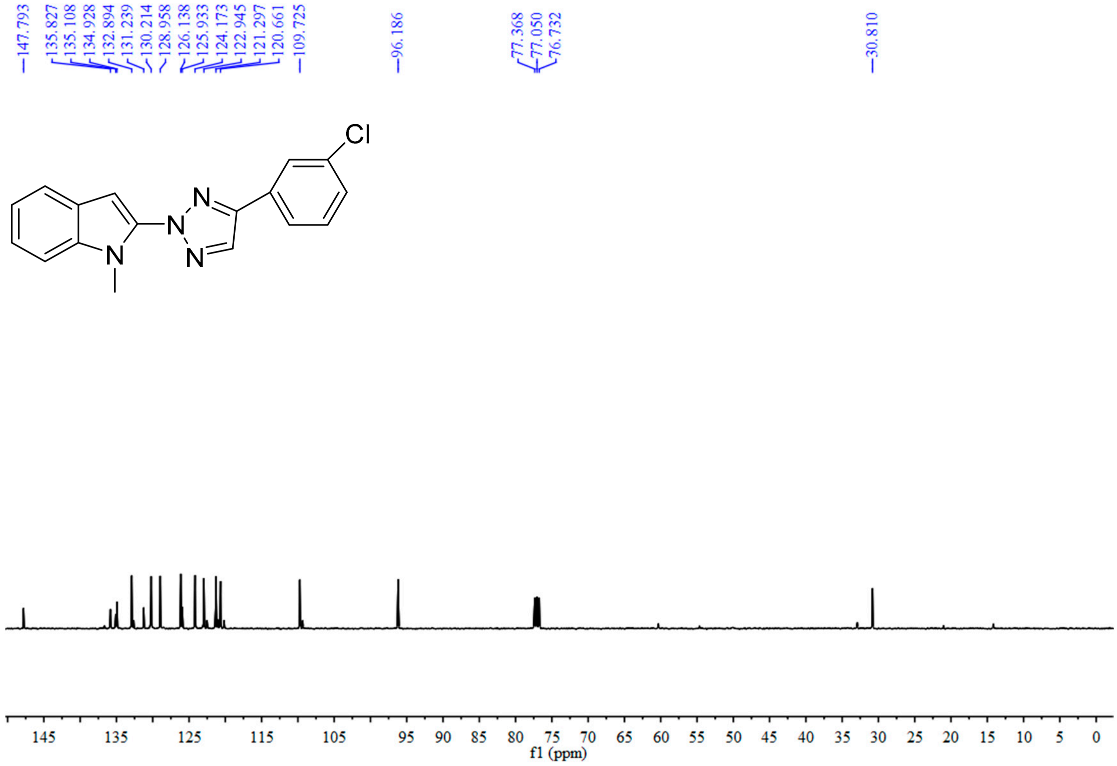
147.793
135.827
135.108
134.928
132.894
131.239
130.214
128.958
126.138
125.933
124.173
122.945
121.297
120.661
109.725
96.186
77.368
77.050
76.732
30.810
f1 (ppm)

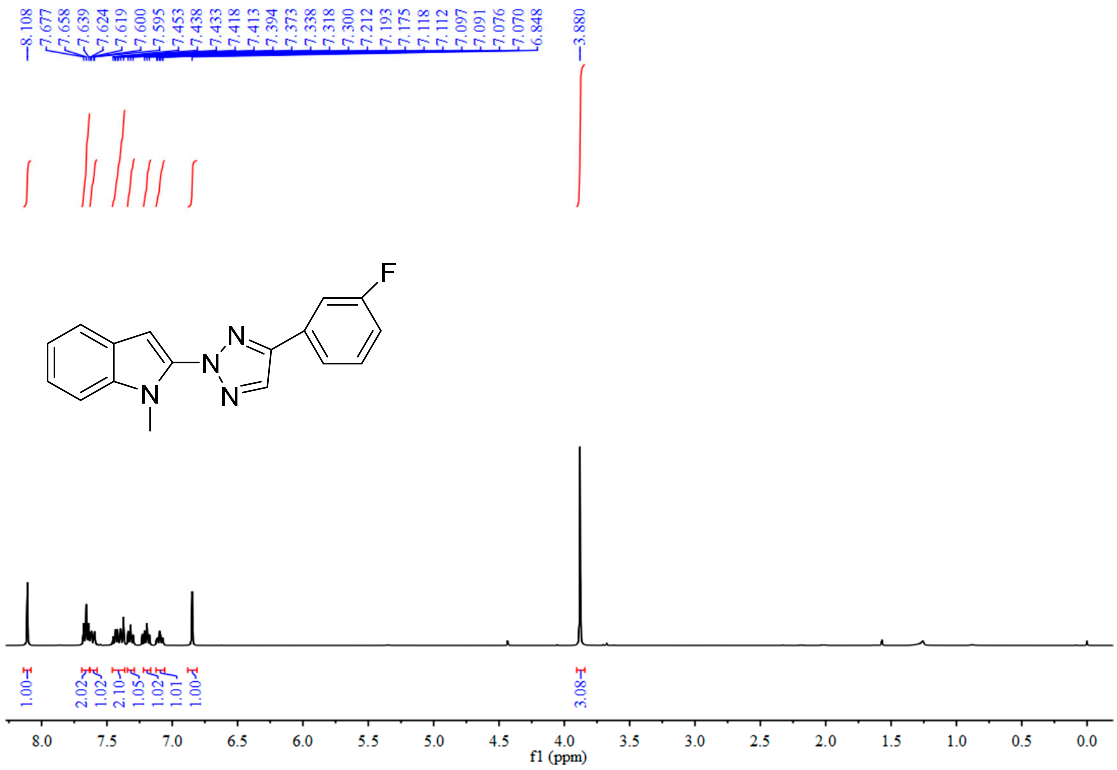
8.108
7.677
7.658
7.639
7.624
7.619
7.600
7.595
7.453
7.438
7.433
7.418
7.413
7.394
7.373
7.338
7.318
7.300
7.212
7.193
7.175
7.118
7.112
7.097
7.091
7.076
7.070
6.848
3.880
F
N
N
N
N
1.00
2.02
1.02
2.10
1.05
1.02
1.01
1.00
3.08
8.0 7.5 7.0 6.5 6.0 5.5 5.0 4.5 4.0 3.5 3.0 2.5 2.0 1.5 1.0 0.5 0.0
f1 (ppm)

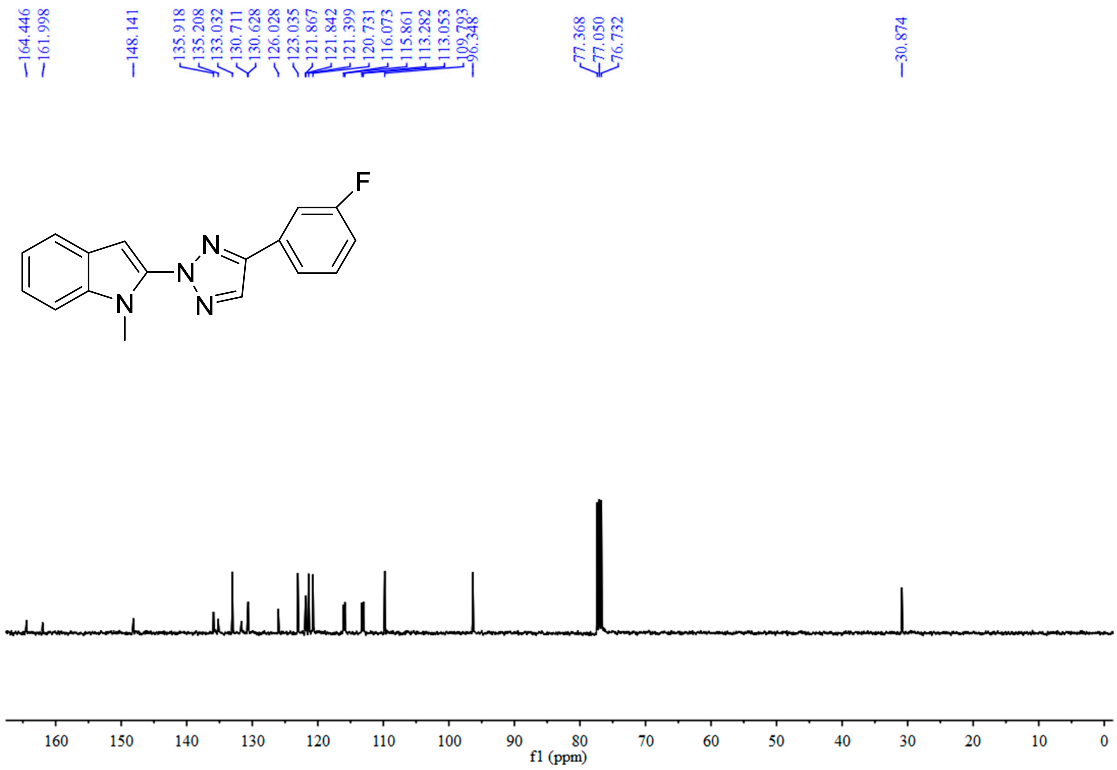
164.446
161.998
148.141
135.918
135.208
133.032
130.711
130.628
126.028
123.035
121.867
121.842
121.399
120.731
116.073
115.861
113.282
113.053
109.793
96.348
77.368
77.050
76.732
30.874
F
N
N
N
N
160 150 140 130 120 110 100 90 80 70 60 50 40 30 20 10 0
f1 (ppm)

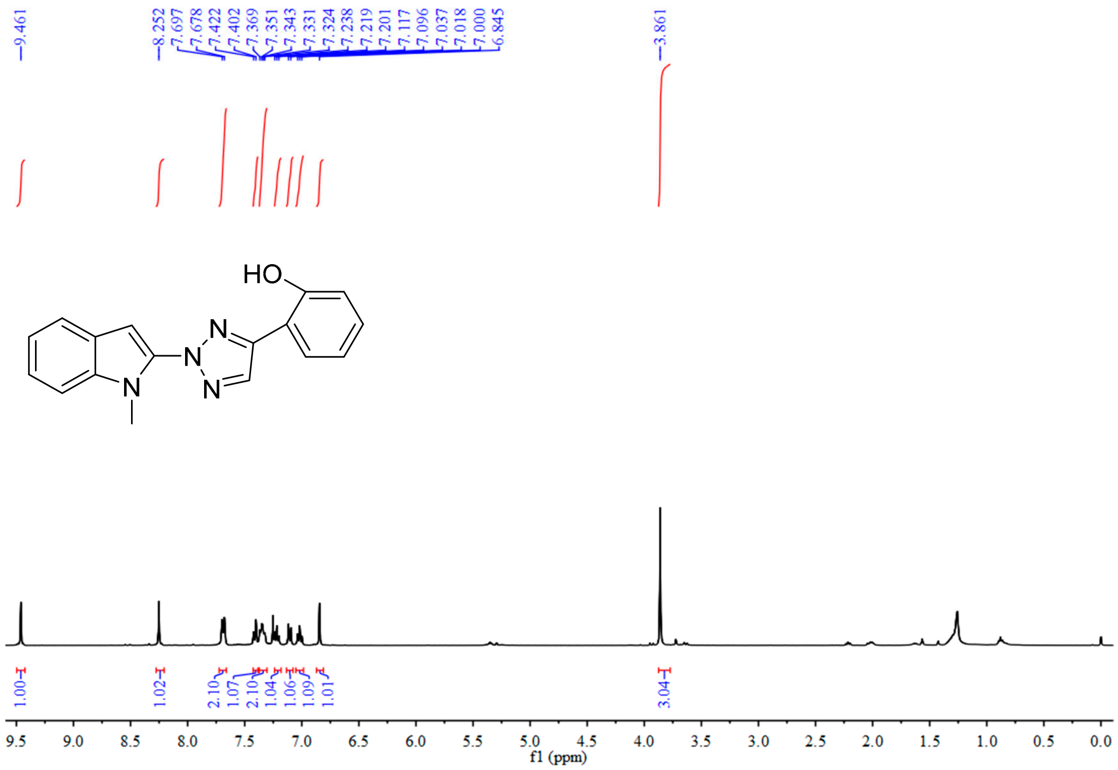
9.461
8.252
7.697
7.678
7.422
7.402
7.369
7.351
7.343
7.331
7.324
7.238
7.219
7.201
7.117
7.096
7.037
7.018
7.000
6.845
3.861
HO
N
N
N
N
1.00
1.02
2.10
1.07
2.10
1.04
1.06
1.09
1.01
3.04
f1 (ppm)

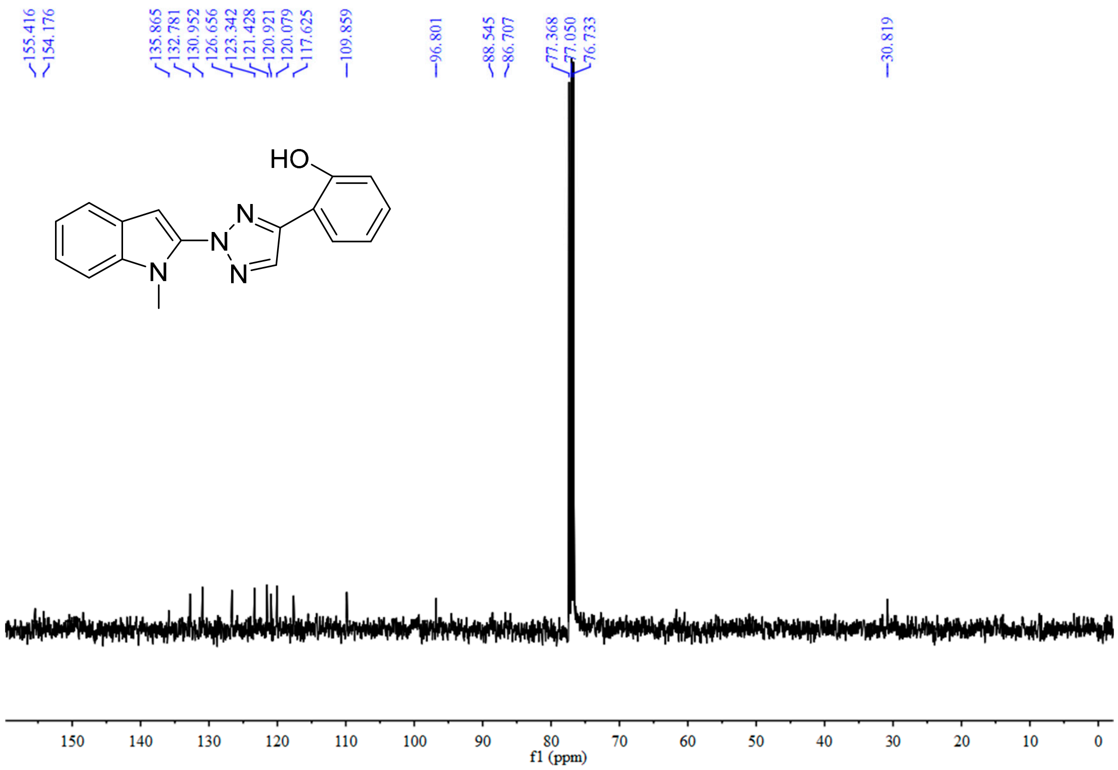
155.416
154.176
135.865
132.781
130.952
126.656
123.342
121.428
120.921
120.079
117.625
109.859
96.801
88.545
86.707
77.368
77.050
76.733
30.819
HO
N
N
N
N
f1 (ppm)

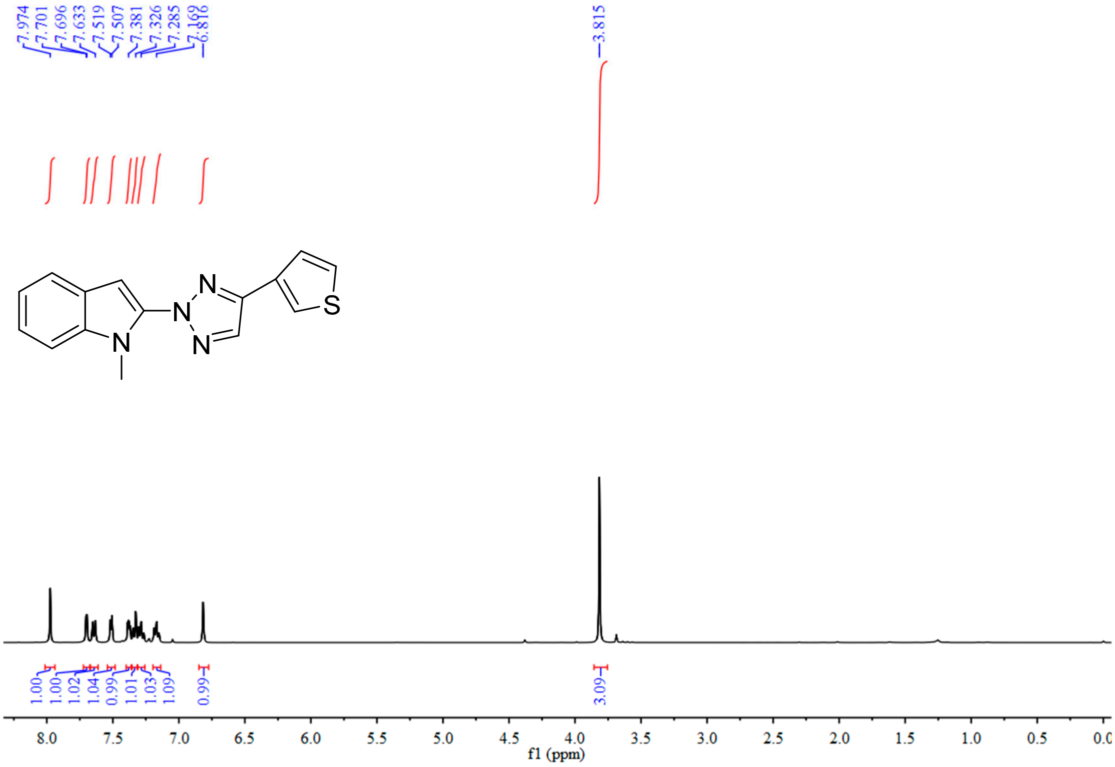

7.974
7.701
7.696
7.633
7.519
7.507
7.381
7.326
7.285
7.169
6.816
3.815
N
N
N
N
S
1.00
1.00
1.02
1.04
0.99
1.01
1.03
1.09
0.99
3.09
f1 (ppm)

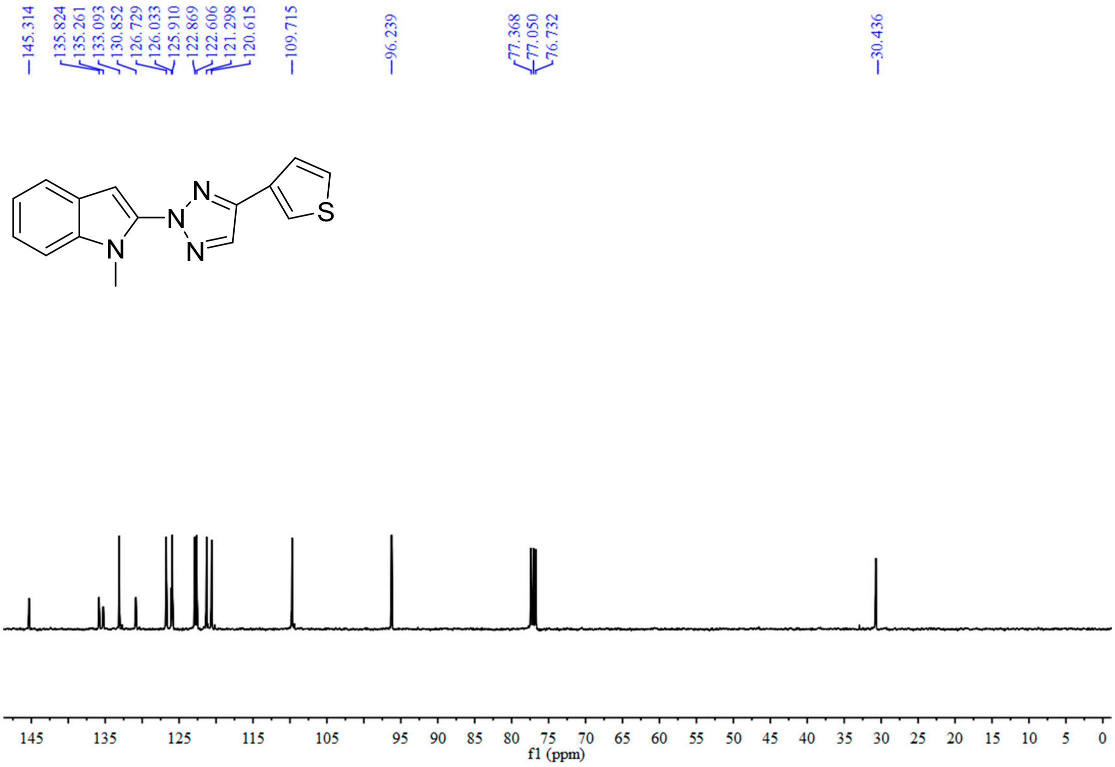

145.314
135.824
135.261
133.093
130.852
126.729
126.033
125.910
122.869
122.606
121.298
120.615
109.715
96.239
77.368
77.050
76.732
30.436
N
N
N
N
S
f1 (ppm)

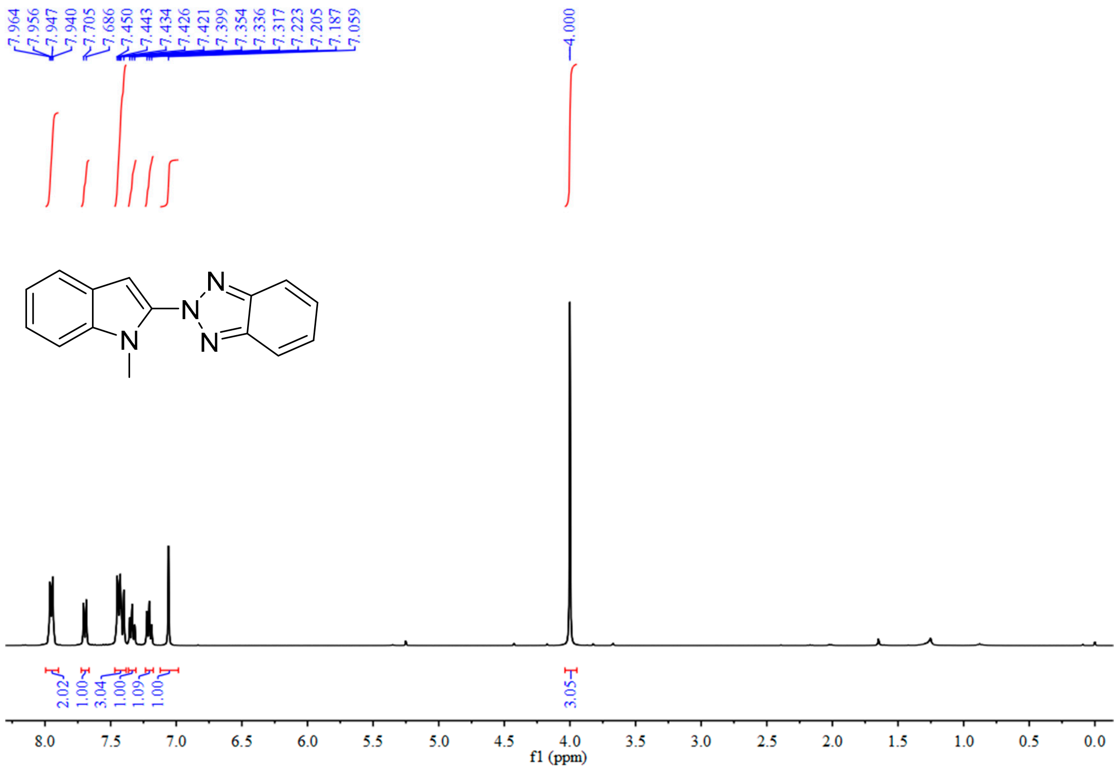

7.964
7.956
7.947
7.940
7.705
7.686
7.450
7.443
7.434
7.426
7.421
7.399
7.354
7.336
7.317
7.223
7.205
7.187
7.059
4.000
2.02
1.00
3.04
1.00
1.09
1.00
3.05
8.0 7.5 7.0 6.5 6.0 5.5 5.0 4.5 4.0 3.5 3.0 2.5 2.0 1.5 1.0 0.5 0.0
f1 (ppm)

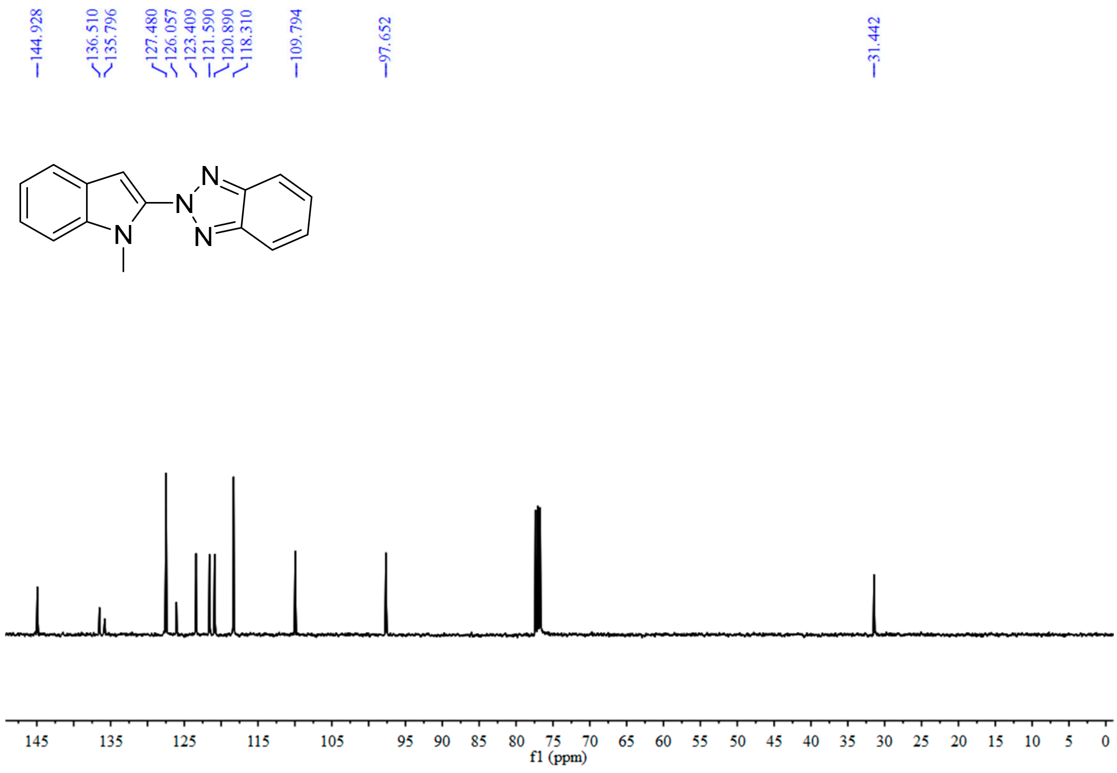

144.928
136.510
135.796
127.480
126.057
123.409
121.590
120.890
118.310
109.794
97.652
31.442
145 135 125 115 105 95 90 85 80 75 70 65 60 55 50 45 40 35 30 25 20 15 10 5 0
f1 (ppm)

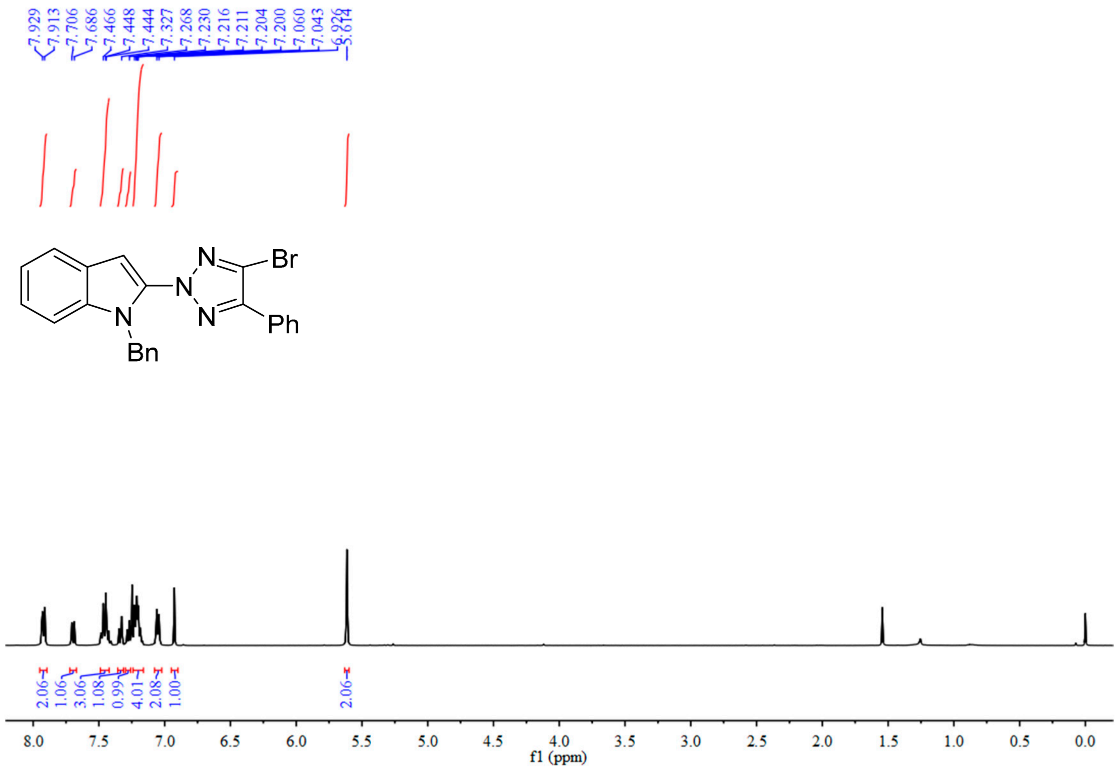

7.929
7.913
7.706
7.686
7.466
7.448
7.444
7.327
7.268
7.230
7.216
7.211
7.204
7.200
7.060
7.043
6.926
5.614
N
N
N
Br
Ph
Bn
2.06
1.06
3.06
1.08
0.99
4.01
2.08
1.00
2.06
f1 (ppm)

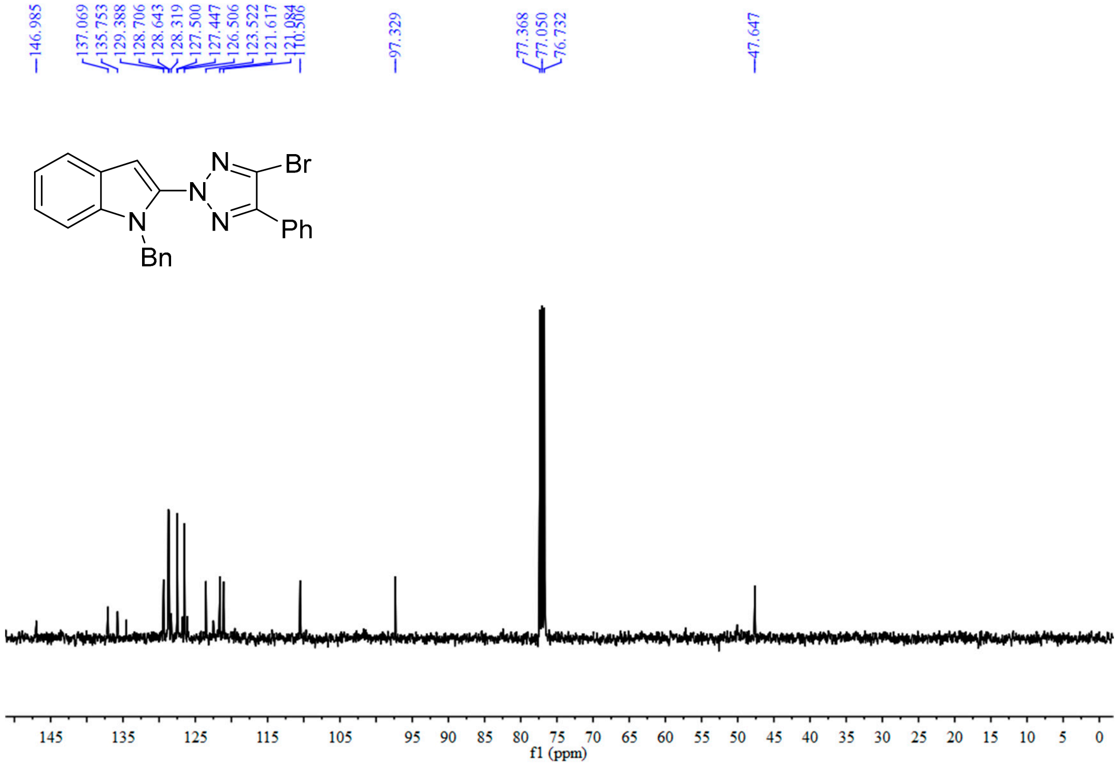

146.985
137.069
135.753
129.388
128.706
128.643
128.319
127.500
127.447
126.506
123.522
121.617
110.506
97.329
77.368
77.050
76.732
47.647
N
N
N
Br
Ph
Bn
f1 (ppm)

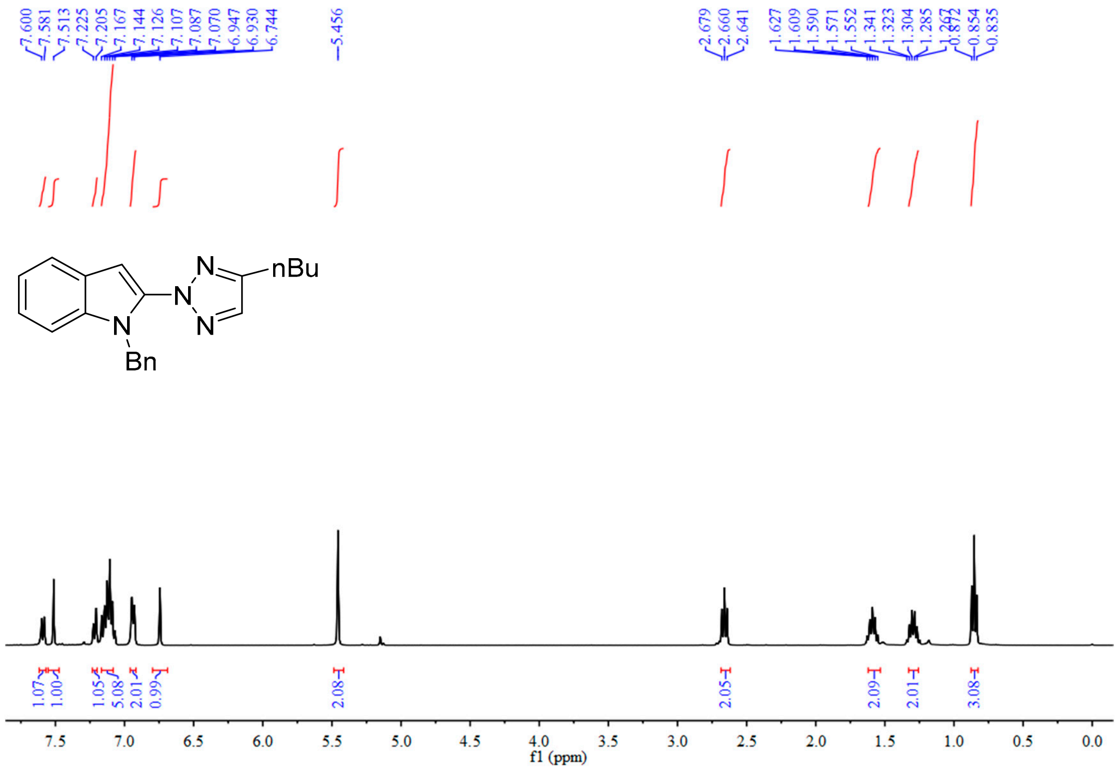

7.600
7.581
7.513
7.225
7.205
7.167
7.144
7.126
7.107
7.087
7.070
6.947
6.930
6.744
5.456
2.679
2.660
2.641
1.627
1.609
1.590
1.571
1.552
1.341
1.323
1.304
1.285
0.854
0.835
nBu
N
N
N
N
Bn
1.07
1.00
1.05
5.08
2.01
0.99
2.08
2.05
2.09
2.01
3.08
7.5 7.0 6.5 6.0 5.5 5.0 4.5 4.0 3.5 3.0 2.5 2.0 1.5 1.0 0.5 0.0
f1 (ppm)

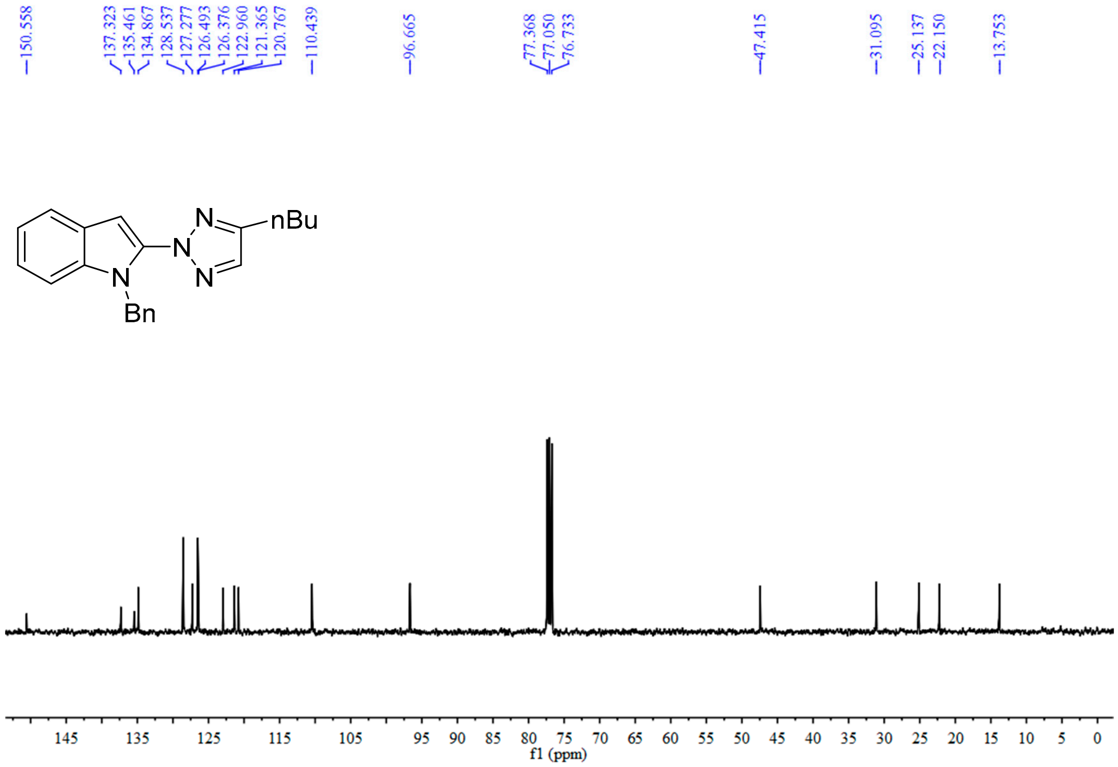

150.558
137.323
135.461
134.867
128.537
127.277
126.493
126.376
122.960
121.365
120.767
110.439
96.665
77.368
77.050
76.733
47.415
31.095
25.137
22.150
13.753
nBu
N
N
N
N
Bn
145 135 125 115 105 95 90 85 80 75 70 65 60 55 50 45 40 35 30 25 20 15 10 5 0
f1 (ppm)

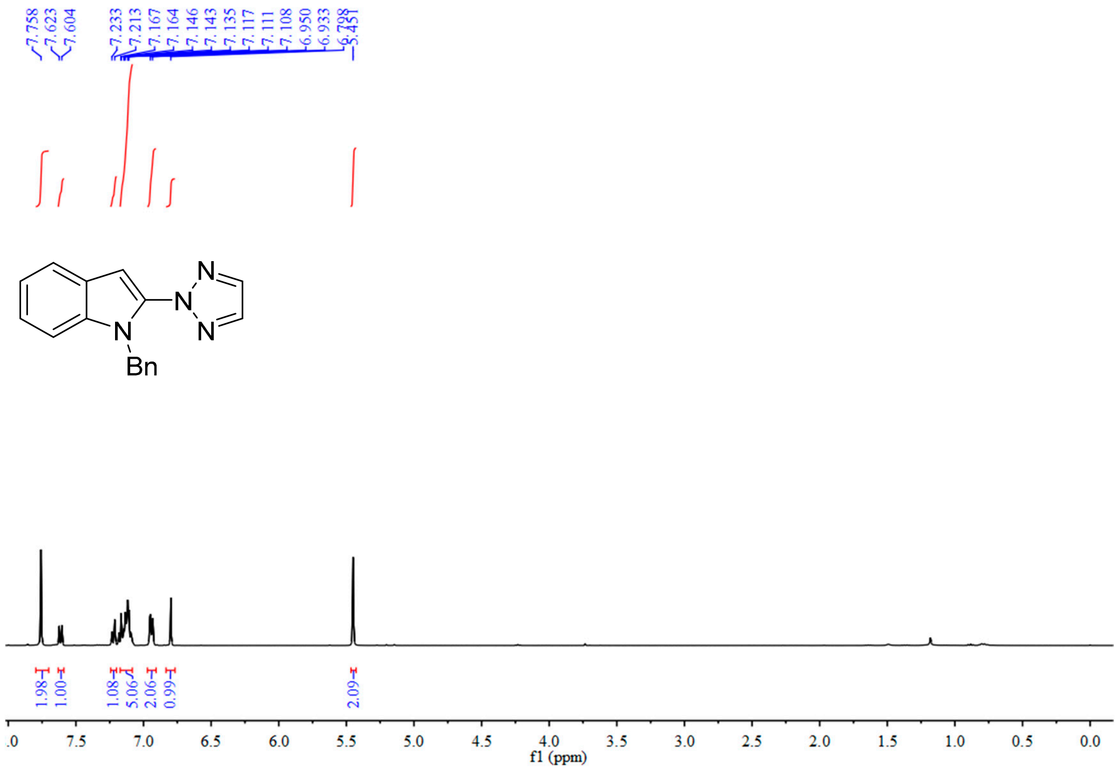
7.758
7.623
7.604
7.233
7.213
7.167
7.164
7.146
7.143
7.135
7.117
7.111
7.108
6.950
6.933
6.798
5.451
N
N
N
N
Bn
1.98
1.00
1.08
5.06
2.06
0.99
2.09
.0
7.5
7.0
6.5
6.0
5.5
5.0
4.5
4.0
3.5
3.0
2.5
2.0
1.5
1.0
0.5
0.0
f1 (ppm)

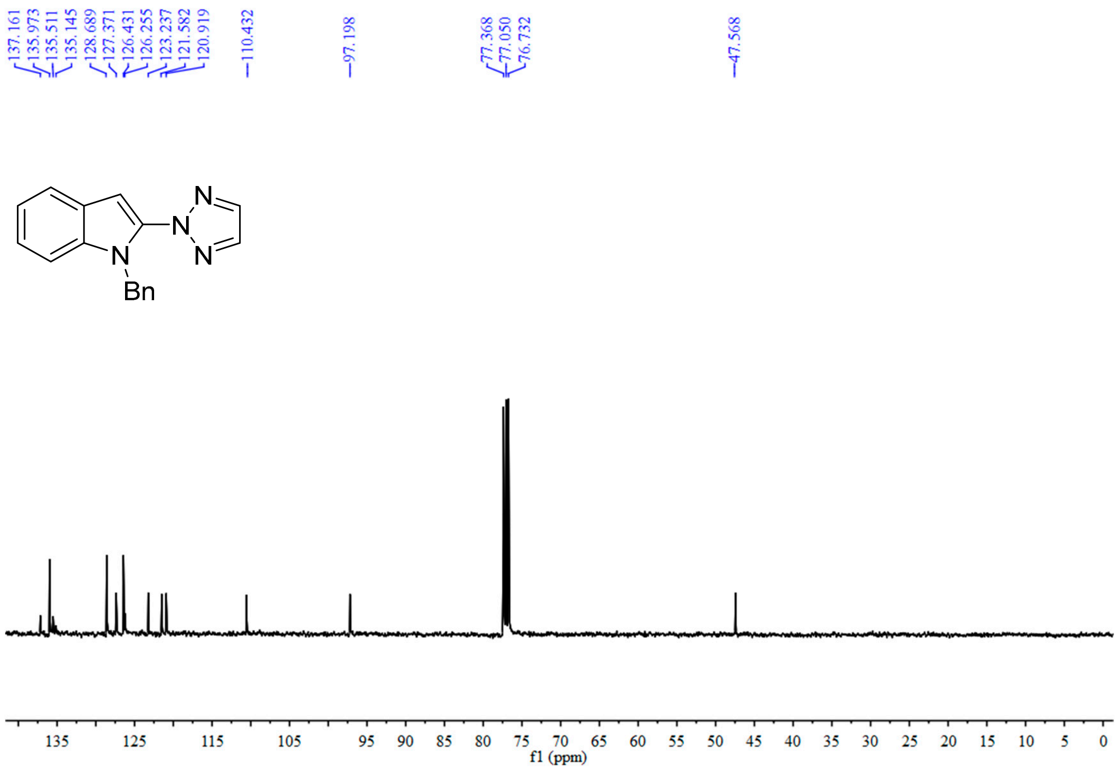
137.161
135.973
135.511
135.145
128.689
127.371
126.431
126.255
123.237
121.582
120.919
110.432
97.198
77.368
77.050
76.732
47.568
N
N
N
N
Bn
135
125
115
105
95
90
85
80
75
70
65
60
55
50
45
40
35
30
25
20
15
10
5
0
f1 (ppm)

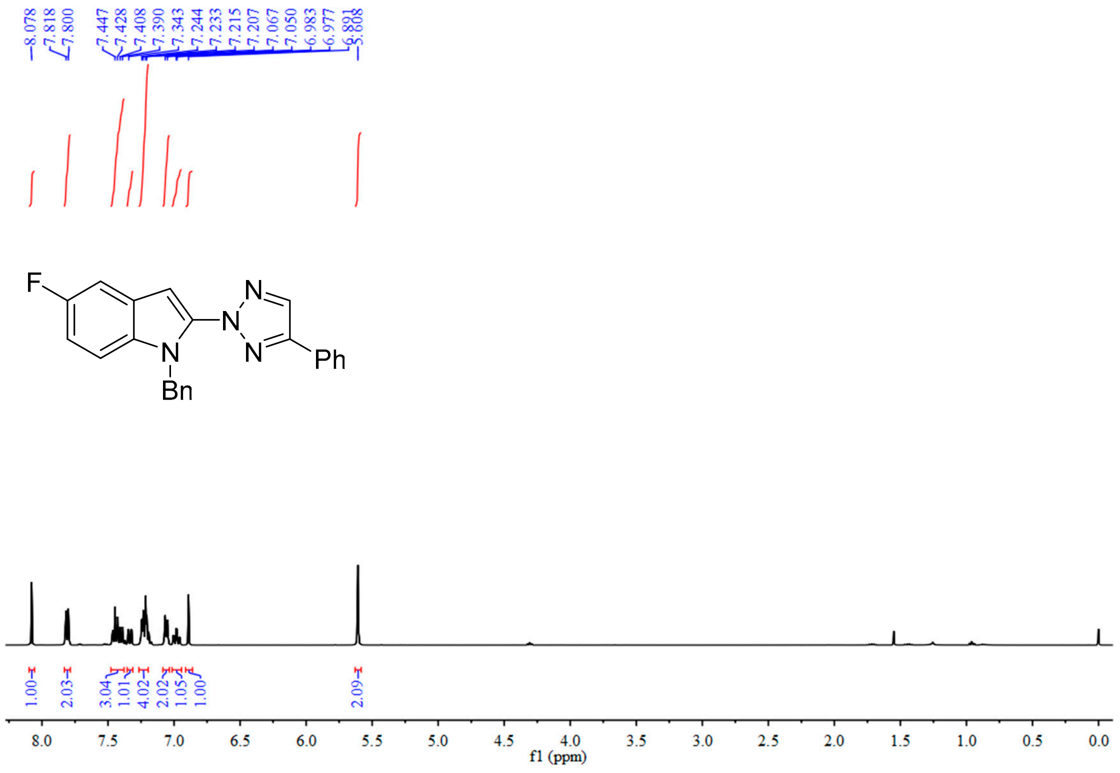

8.078
7.818
7.800
7.447
7.428
7.408
7.390
7.343
7.244
7.233
7.215
7.207
7.067
7.050
6.983
6.977
6.891
5.608
F
N
N
N
N
Ph
Bn
1.00
2.03
3.04
1.01
4.02
2.02
1.05
1.00
2.09
8.0
7.5
7.0
6.5
6.0
5.5
5.0
4.5
4.0
3.5
3.0
2.5
2.0
1.5
1.0
0.5
0.0
f1 (ppm)

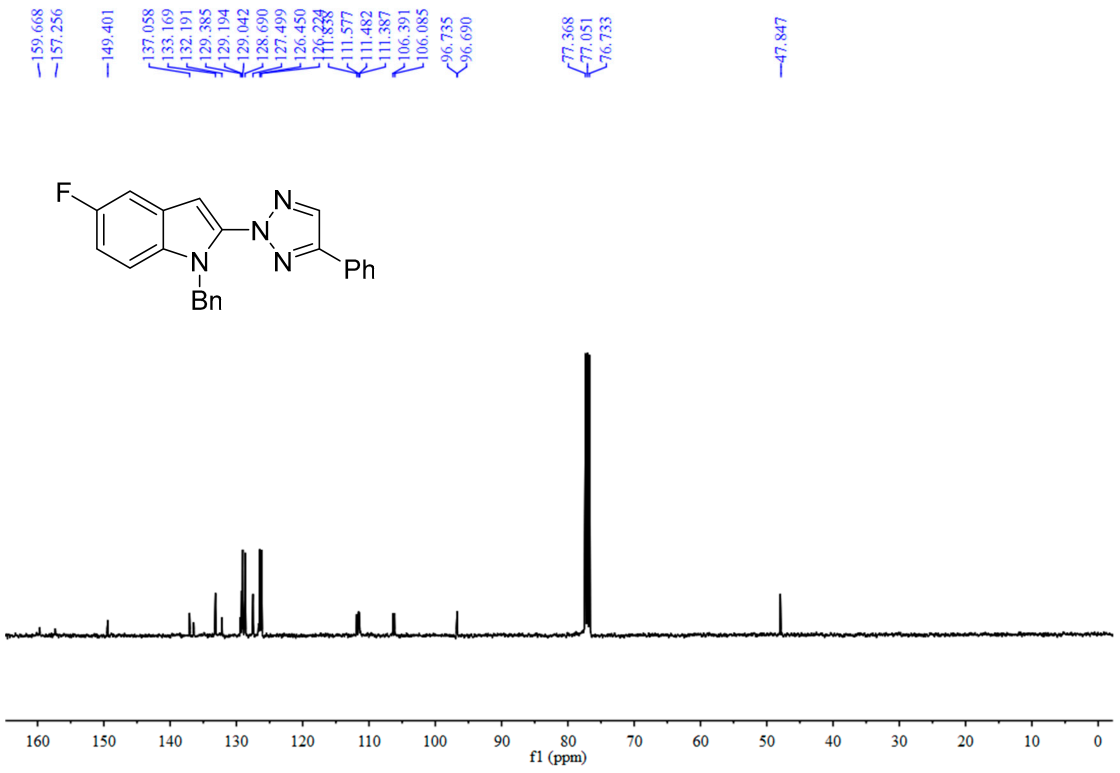

159.668
157.256
149.401
137.058
133.169
132.191
129.385
129.194
129.042
128.690
127.499
126.450
111.577
111.482
111.387
106.391
106.085
96.735
96.690
77.368
77.051
76.733
47.847
F
N
N
N
N
Ph
Bn
160
150
140
130
120
110
100
90
80
70
60
50
40
30
20
10
0
f1 (ppm)

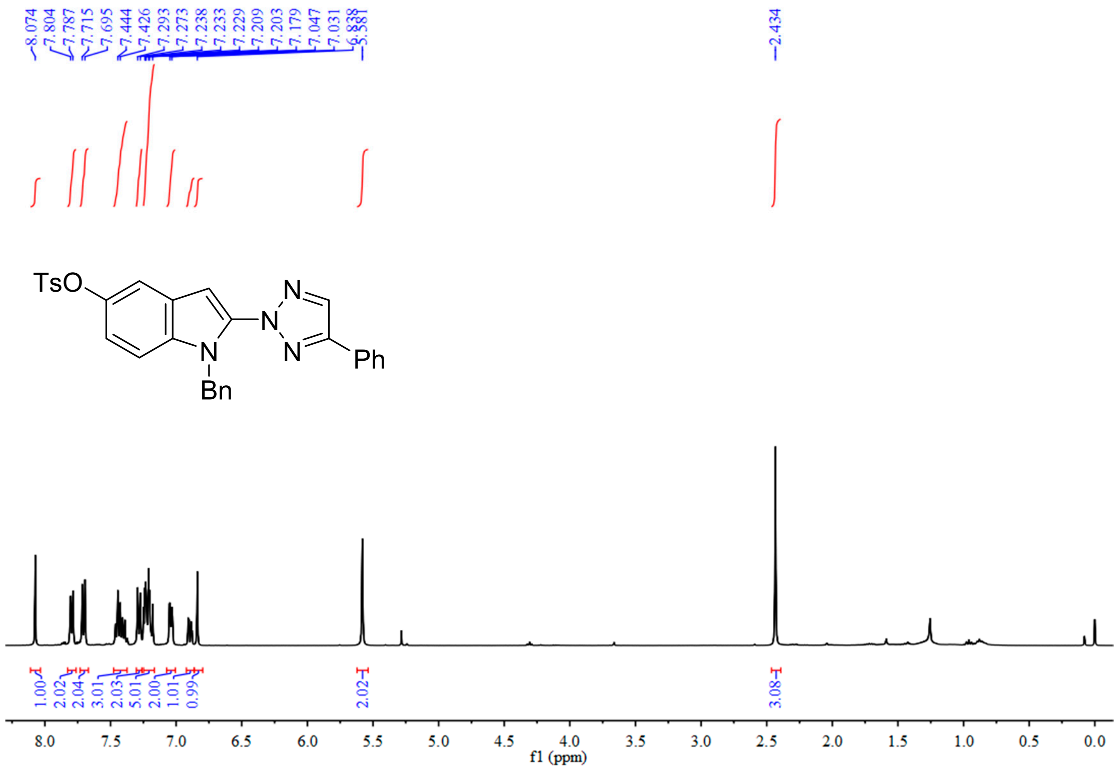
8.074
7.804
7.787
7.715
7.695
7.444
7.426
7.293
7.273
7.238
7.233
7.229
7.209
7.203
7.179
7.047
7.031
6.838
5.581
2.434
TsO
N
N
N
N
Ph
Bn
1.00
2.02
2.04
3.01
2.03
5.01
2.00
1.01
0.99
2.02
3.08
f1 (ppm)

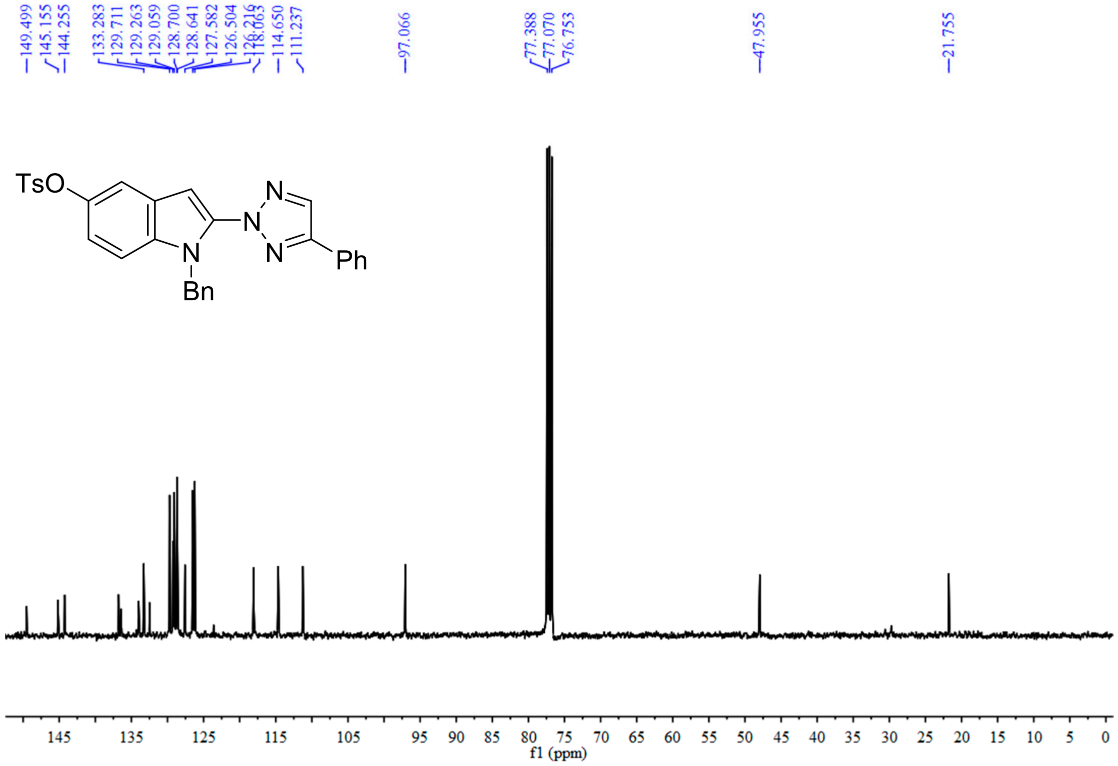
149.499
145.155
144.255
133.283
129.711
129.263
129.059
128.700
128.641
127.582
126.504
118.063
114.650
111.237
97.066
77.388
77.070
76.753
47.955
21.755
TsO
N
N
N
N
Ph
Bn
f1 (ppm)

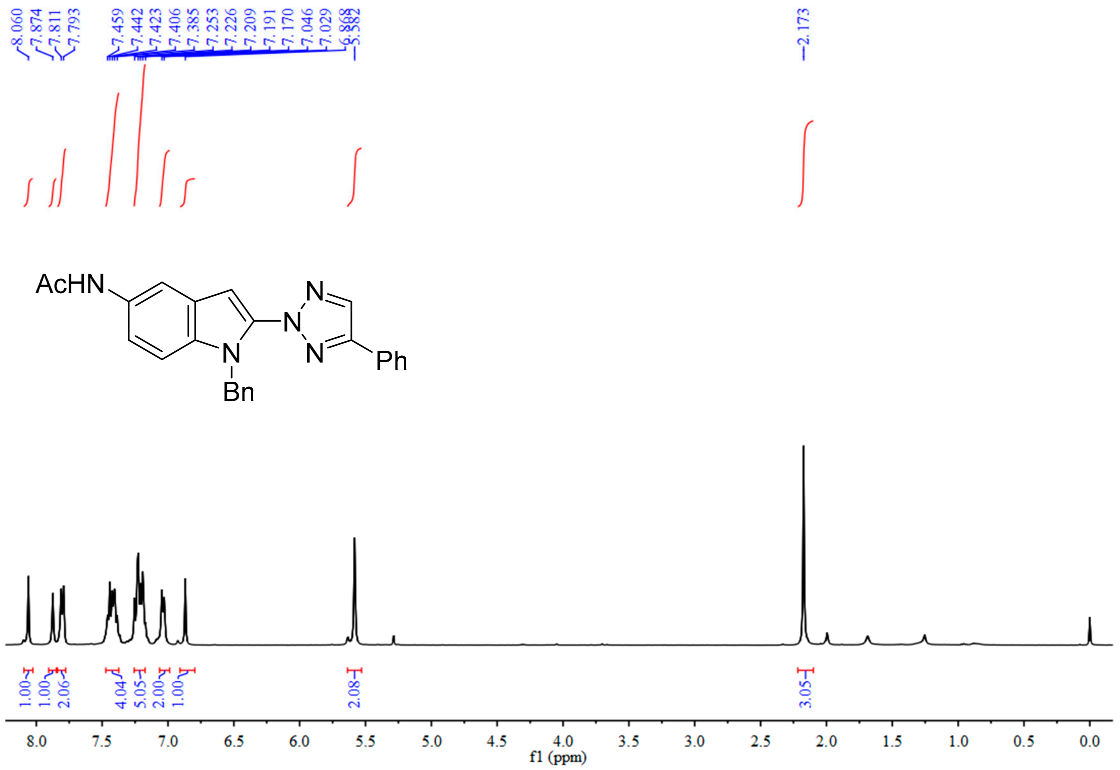

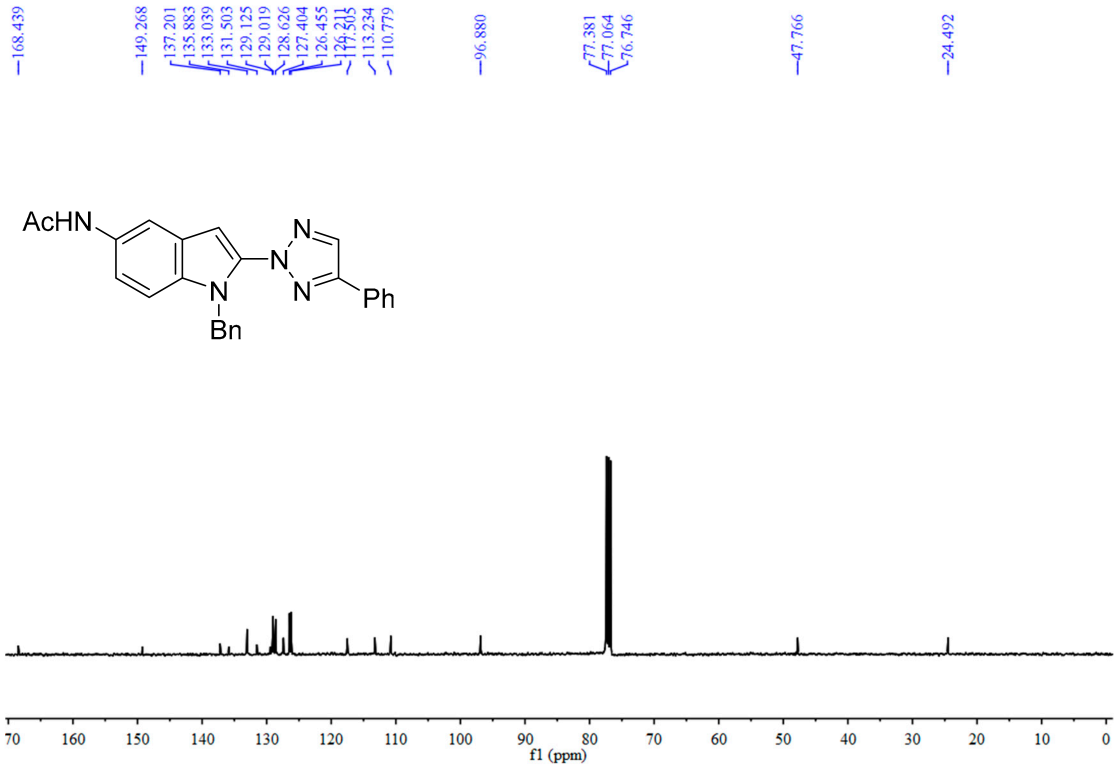

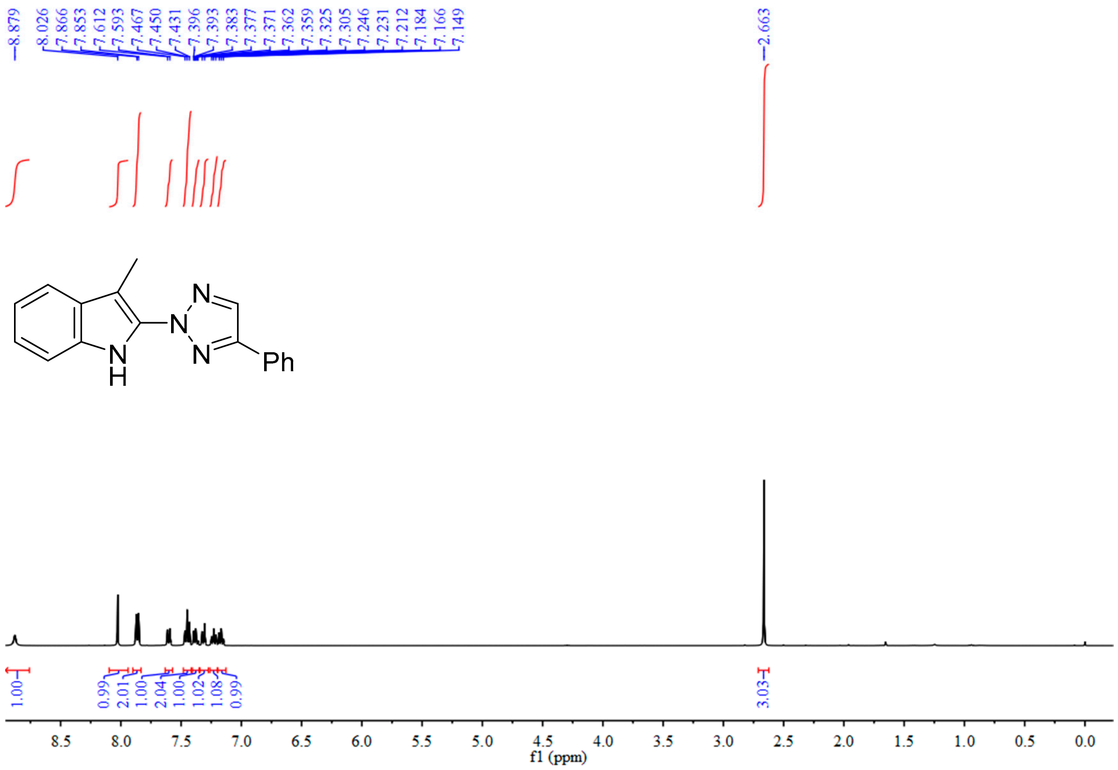
8.879
8.026
7.866
7.853
7.612
7.593
7.467
7.450
7.431
7.396
7.393
7.383
7.377
7.371
7.362
7.359
7.325
7.305
7.246
7.231
7.212
7.184
7.166
7.149
2.663
N
N
N
N
H
Ph
1.00
0.99
2.01
1.00
2.04
1.00
1.02
1.08
0.99
3.03
f1 (ppm)

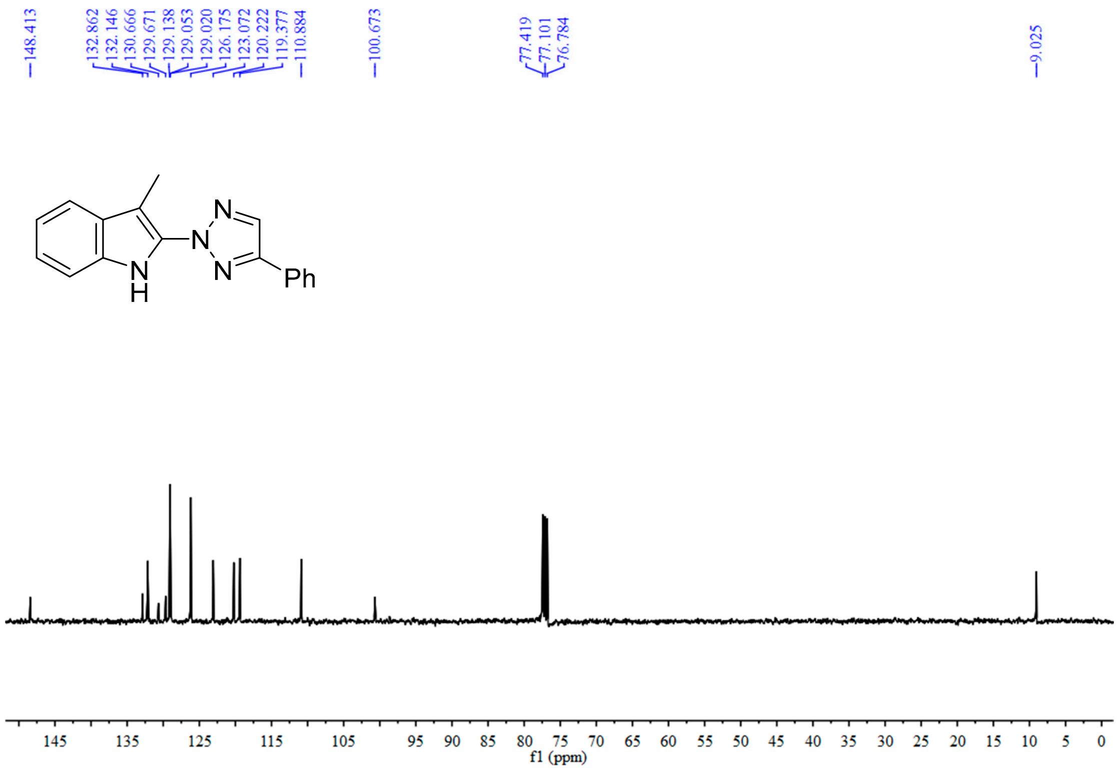
148.413
132.862
132.146
130.666
129.671
129.138
129.053
129.020
126.175
123.072
120.222
119.377
110.884
100.673
77.419
77.101
76.784
9.025
N
N
N
N
H
Ph
f1 (ppm)

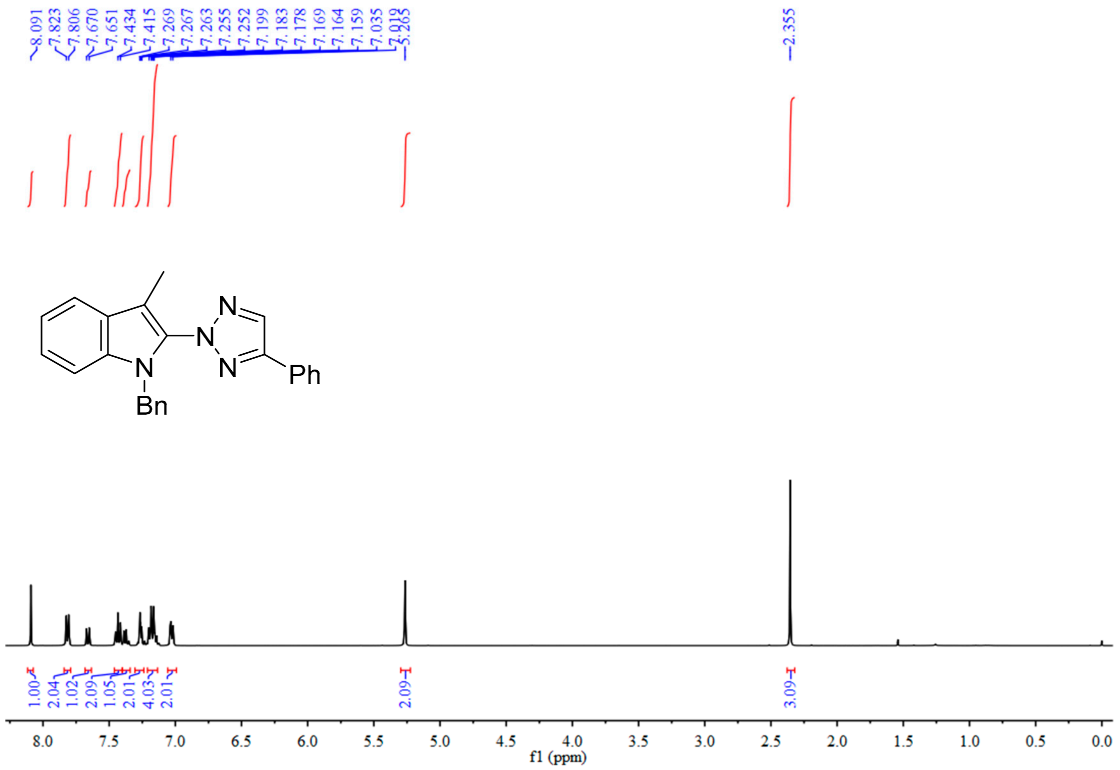

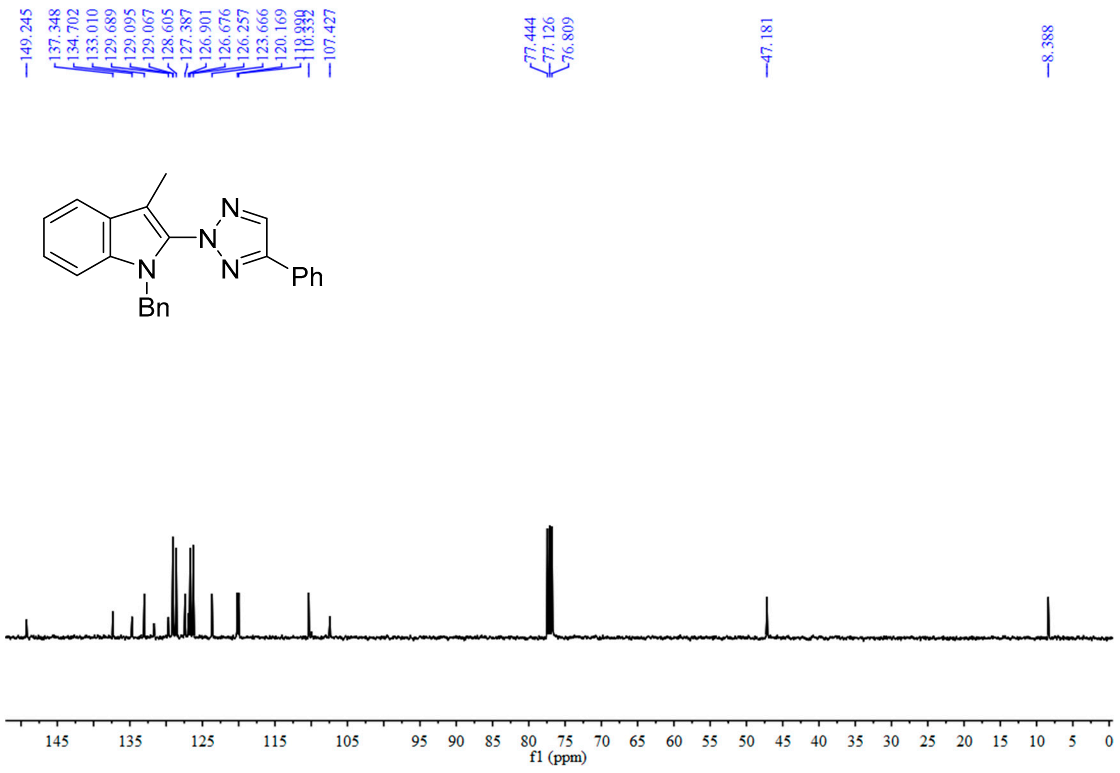

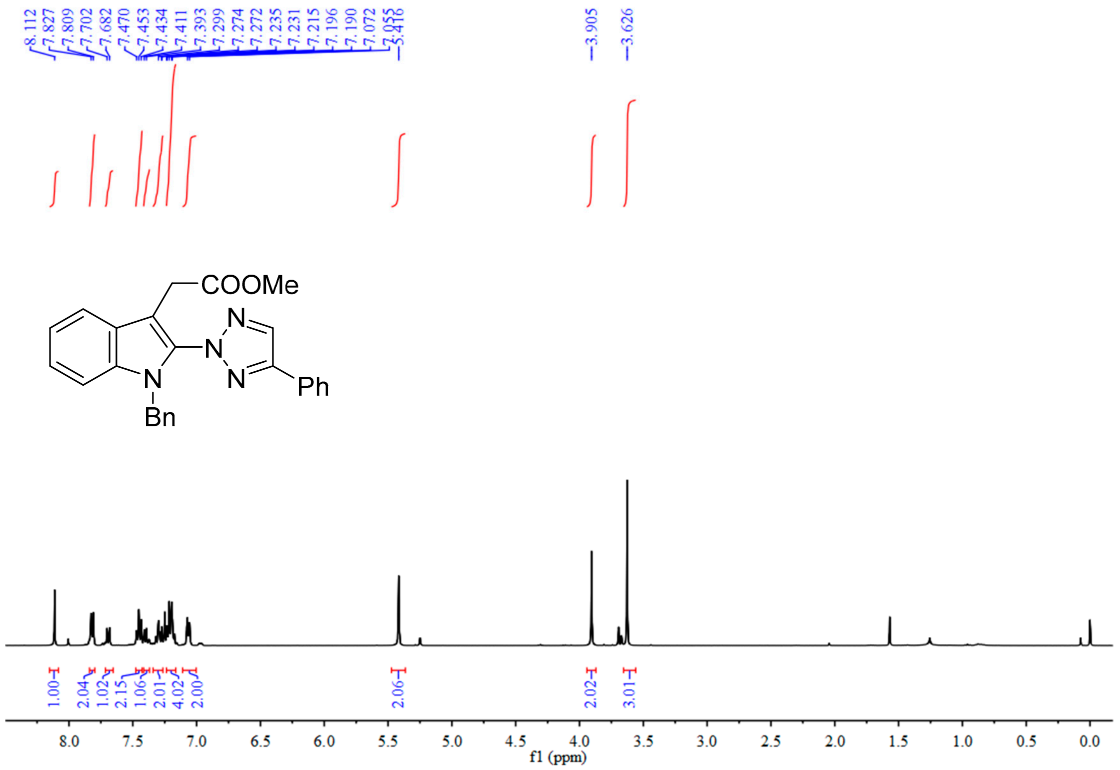

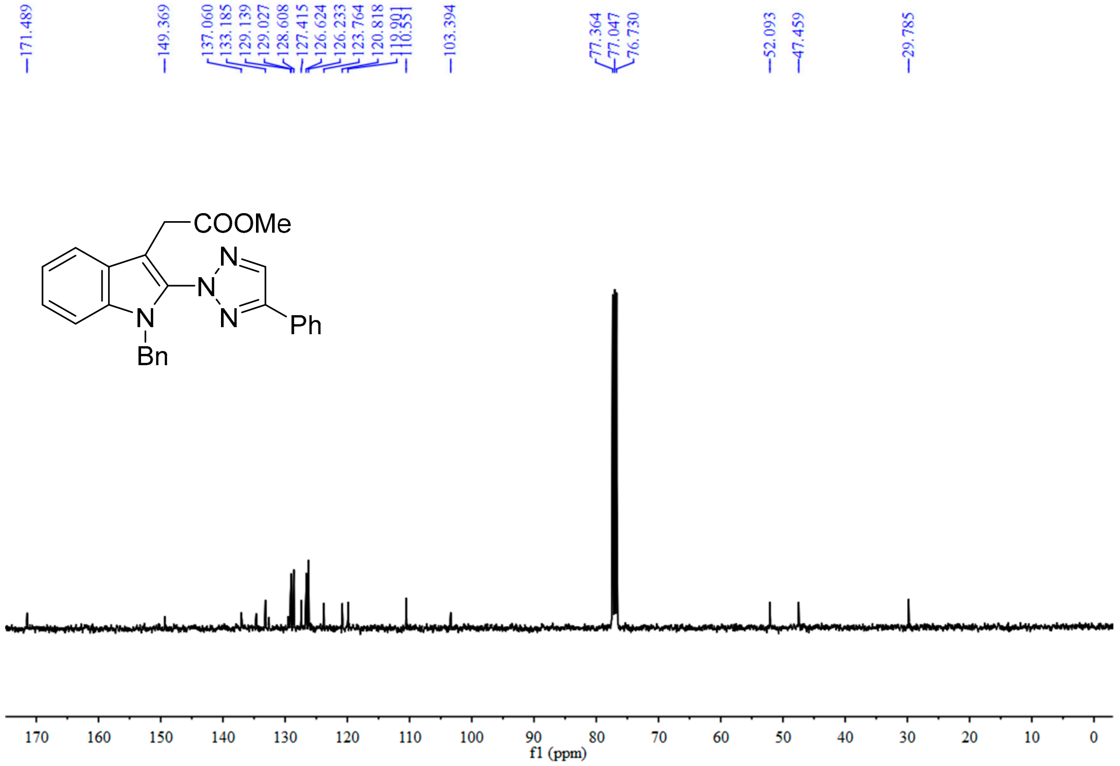

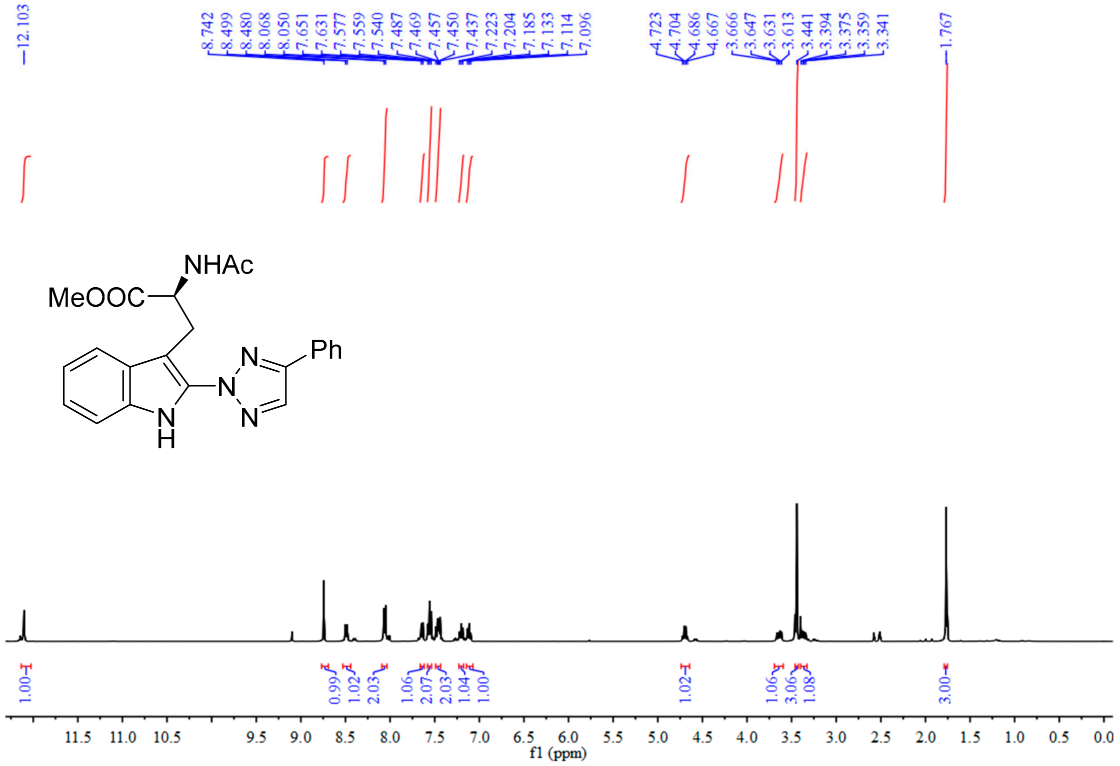

MeOOC
NHAc
N
N
N
Ph
N
H
f1 (ppm)

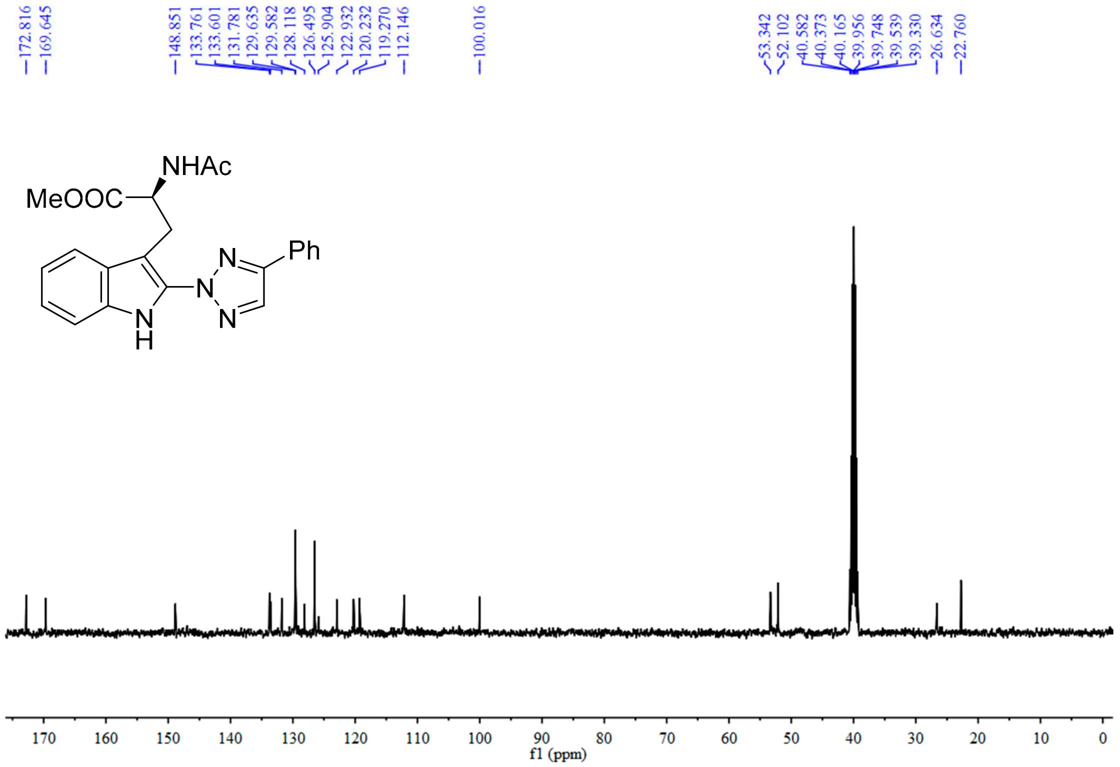

MeOOC
NHAc
N
N
N
Ph
N
H
f1 (ppm)

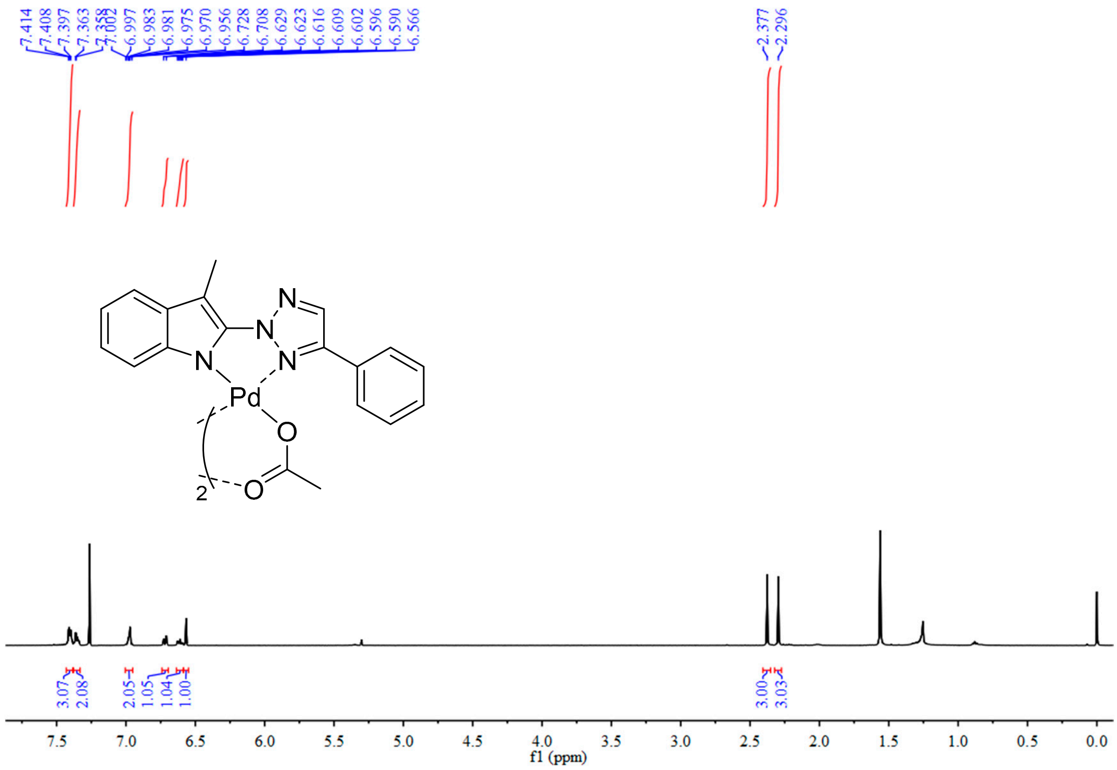
7.414
7.408
7.397
7.363
7.358
7.002
6.997
6.983
6.981
6.975
6.970
6.956
6.728
6.708
6.629
6.623
6.616
6.609
6.602
6.596
6.590
6.566
2.377
2.296
N
N
N
N
Pd
O
O
2
3.07
2.08
2.05
1.05
1.04
1.00
3.00
3.03
7.5
7.0
6.5
6.0
5.5
5.0
4.5
4.0
3.5
3.0
2.5
2.0
1.5
1.0
0.5
0.0
f1 (ppm)

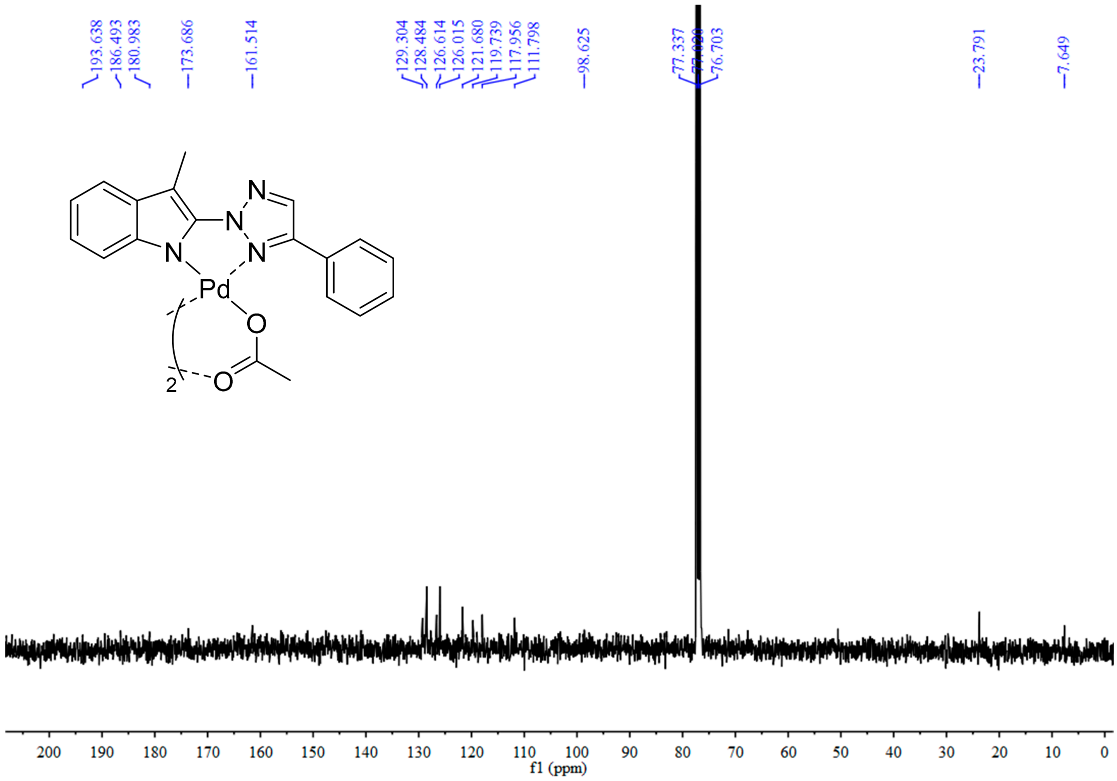
193.638
186.493
180.983
173.686
161.514
129.304
128.484
126.614
126.015
121.680
119.739
117.956
111.798
98.625
77.337
76.703
23.791
7.649
N
N
N
N
Pd
O
O
2
200
190
180
170
160
150
140
130
120
110
100
90
80
70
60
50
40
30
20
10
0
f1 (ppm)
